# Projecting tuberculosis control progress in metropolitan and non-metropolitan areas of Brazil, 2001–2035: a Bayesian age-period-cohort analysis

**DOI:** 10.1186/s40249-025-01400-x

**Published:** 2025-12-22

**Authors:** José Mário Nunes da Silva, Fredi Alexander Diaz-Quijano, Mauro Niskier Sanchez, Walter Massa Ramalho

**Affiliations:** 1 Center for Tropical Medicine, School of Medicine, University of Brasília, Brasília, DF Brazil; 2 Departament of Epidemiology – Laboratório de Inferência Causal em Epidemiologia (LINCE-USP), School of Public Health, University of São Paulo, São Paulo, SP Brazil; 3 School of Health Sciences, University of Brasília, Brasília, DF Brazil; 4 School of Health Sciences and Technology, University of Brasília, Brasília, DF Brazil

**Keywords:** Pulmonary tuberculosis, Bayesian age-period-cohort, Incidence, Projections, Trend, Urban population

## Abstract

**Background:**

Despite advancements in tuberculosis (TB) control policies in Brazil, the disease remains a significant public health concern. This study aimed to analyze long-term trends and projections of pulmonary tuberculosis (PTB) incidence rates in metropolitan and non-metropolitan areas of Brazil from 2001 to 2035, as well as to quantify the contributions of demographic and epidemiological changes to these patterns.

**Methods:**

This ecological study used national PTB case notification data reported to Brazil’s Notifiable Diseases Information System from 2001 to 2020. Joinpoint regression was applied to identify changes in temporal trends. Age-period-cohort models were employed to examine the effects of age, period, and birth cohort on disease risk. A decomposition analysis was then conducted to assess the contributions of population aging, demographic growth, and epidemiological changes. Finally, Bayesian age-period-cohort models were used to project the TB burden through 2035, stratified by sex and area.

**Results:**

Between 2001 and 2020, PTB incidence declined by an average of − 2.67% (95% *CI* − 3.43, − 2.08) per year in metropolitan areas and − 2.54% (95% *CI* − 2.92, − 2.16) in non-metropolitan areas of Brazil. However, decomposition analysis showed that the absolute number of PTB cases in metropolitan areas increased, primarily driven by population growth (+ 21,610 cases in men; + 10,545 in women), with a smaller contribution from population aging (+ 2649 and + 521 cases, respectively). In non-metropolitan areas, reductions were mainly explained by epidemiological improvements (− 8314 cases in men; − 6663 in women) and population decline (− 4972 and − 2380 cases, respectively), outweighing the effects of aging. Looking ahead, projections indicate that PTB incidence will rise in metropolitan areas, from 52.6 in 2015 to 62.4 [95% credible interval (CrI): 37.1–87.8] per 100,000 by 2035, while stabilizing at relatively high levels in non-metropolitan areas, increasing from 28.4 to 33.8 per 100,000 (95% CrI: 19.3–48.3) among men.

**Conclusion:**

Metropolitan areas are projected to experience substantial increases in PTB incidence, while non-metropolitan regions are expected to stabilize at persistently high levels, particularly among men. The findings indicate that current TB control efforts in Brazil need to be strengthened for the country to meet the 2035 targets, especially in metropolitan areas.

**Graphical Abstract:**

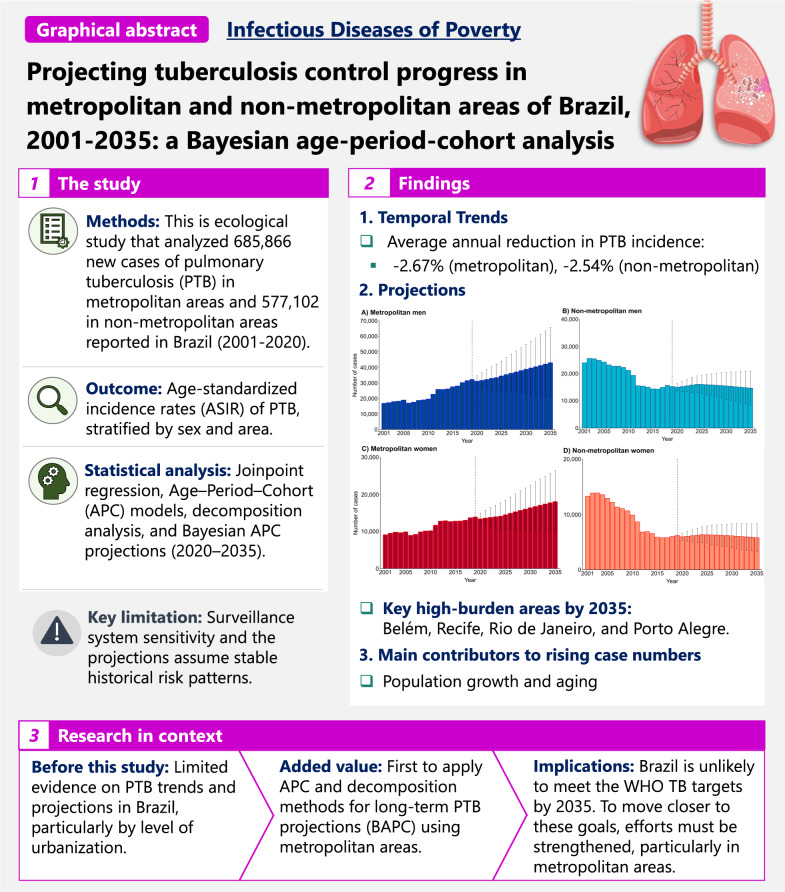

**Supplementary Information:**

The online version contains supplementary material available at 10.1186/s40249-025-01400-x.

## Background

Tuberculosis (TB) remains one of the leading challenges in global public health [1]. In Brazil, more than 84,000 new cases were reported in 2024, with an incidence rate of 39.7 per 100,000 population, ranking the country among the 30 high TB burden nations prioritized by the World Health Organization (WHO) [2]. In alignment with global efforts, Brazil has committed to the WHO’s End TB Strategy, which aims to reduce TB incidence by 90% and TB-related mortality by 95% by 2035 [1, 2]. Nevertheless, recent studies show that the country remains far from these targets, mainly due to persistent structural barriers that hinder timely diagnosis and effective treatment [3, 4].

Within the national context, the contrast between metropolitan and non-metropolitan areas reveals important differences in factors influencing TB transmission, including urbanization, healthcare access, population density, housing conditions, and poverty [5, 6]. Although metropolitan areas concentrate greater infrastructure, they also contain vast pockets of social vulnerability, often with limited access to care [7, 8]. In contrast, non-metropolitan areas face challenges related to the coverage, quality, and continuity of primary healthcare [9, 10]. Despite these distinct dynamics, comprehensive national studies analyzing pulmonary tuberculosis (PTB) rates by level of urbanization remain scarce. In particular, few studies compare current trends and future projections while exploring the demographic and epidemiological determinants underlying these disparities.

In countries like Brazil, marked by regional inequalities, disaggregating analyses by geographic area is essential to guide more equitable and effective interventions, strengthening TB surveillance, prevention, and treatment strategies [9, 11]. In this context, age-period-cohort models offer a robust analytical framework, allowing the decomposition of effects related to age, period, and birth cohort on disease incidence [12–14]. When applied within a Bayesian approach, these models yield more robust projections by incorporating smoothing and uncertainty [15–17]. Decomposition analysis complements this strategy by quantifying the contributions of demographic and epidemiological factors to the disease burden. Thus, this study aimed to analyze long-term trends and projections of PTB incidence rates in metropolitan and non-metropolitan areas of Brazil from 2001 to 2035, as well as to quantify the contributions of population aging, demographic growth, and epidemiological changes to these patterns.

## Methods

### Study design

This ecological study used metropolitan and non-metropolitan areas of Brazil as units of analysis, covering all 5570 municipalities. The study is reported in accordance with the RECORD guidelines [18].

### Setting

**Fig. 1:**
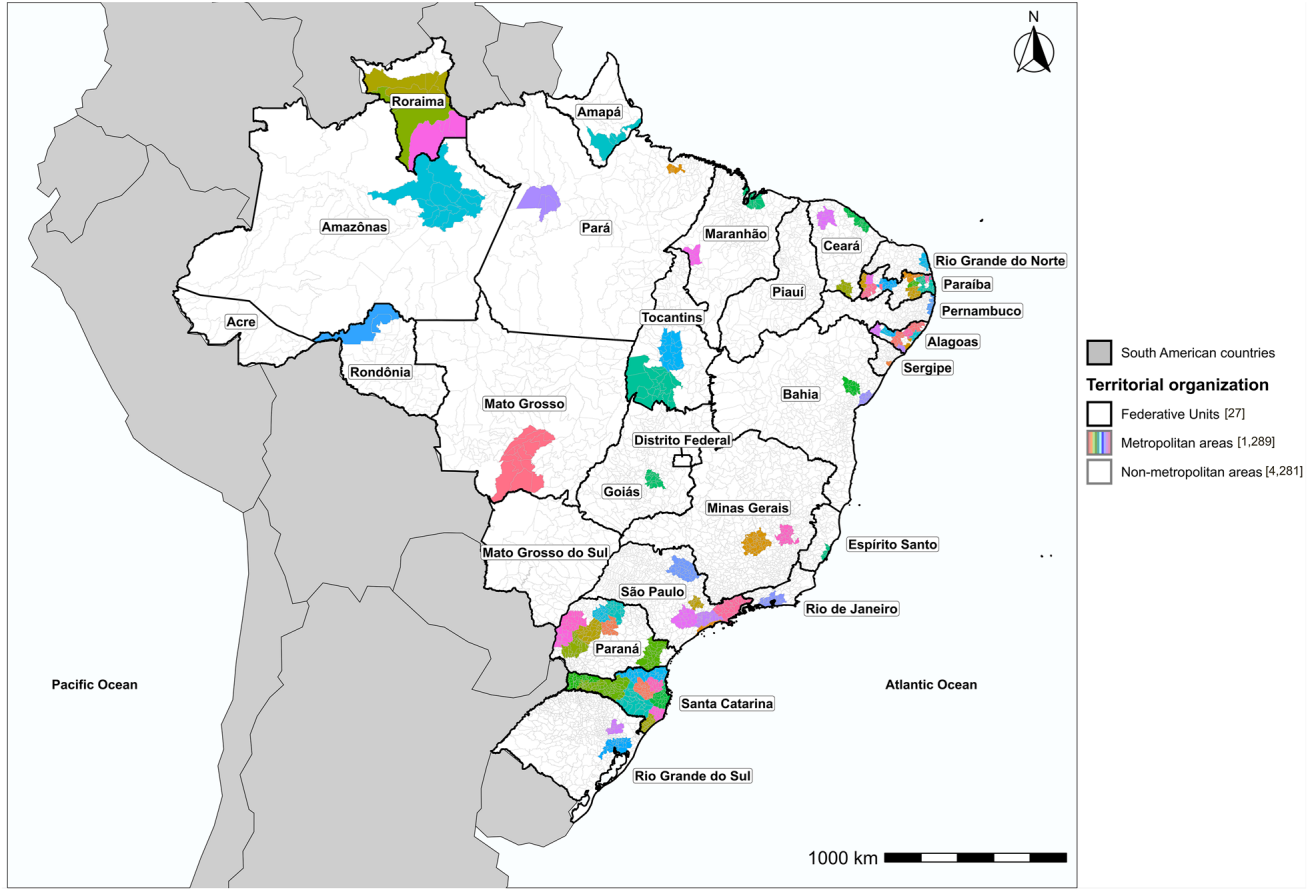
Map of Brazil showing its territorial organization into 27 federative units, 74 metropolitan areas, and 5570 municipalities, 2020. Values in brackets indicate the total number of federative units, and for areas, the total number of municipalities

Brazil, located in South America, had an estimated population of 209.2 million in 2020. The country is divided into five major geographic regions; North, Northeast, Central-West, Southeast, and South; which together comprise 26 states and the Federal District [19]. As of December 2020, Brazil had 74 metropolitan areas, defined as groupings of neighboring municipalities established by state complementary law (Article 25, §3 of the Federal Constitution), with the purpose of integrating the organization, planning, and execution of public functions of common interest (Fig. 1) [20]. These areas accounted for approximately 53% (110.9 million) of the Brazilian population and included about 23% (1289) of the country’s municipalities. The state of Paraíba had the highest number of metropolitan areas (12), followed by Santa Catarina (11), Alagoas (9), and Paraná (8) (Table S1).

### Study population and data source

The study included all new cases of PTB, clinically diagnosed or laboratory confirmed, reported between 2001 and 2020 in the Notifiable Diseases Information System (SINAN), managed by the Department of Informatics of the Unified Health System (DATASUS). SINAN is the national Brazilian system responsible for collecting, recording, processing, and disseminating data on notifiable diseases across the entire country, including TB, and plays a key role in public health surveillance [21]. The dataset used in this study is open access, publicly available through the DATASUS platform (http://www2.datasus.gov.br), provided in anonymized format without individual identifiers. Thus, the study did not require an authorization certificate, informed consent, or review by an Ethics Committee.

We obtained census data, as well as intercensal population estimates and projections, produced by the Brazilian Institute of Geography and Statistics (IBGE), disaggregated by age group, sex, and year, via DATASUS (https://www.gov.br/saude/pt-br/composicao/seidigi/demas/dados-populacionais). It is important to note that the composition of metropolitan areas changed over the study period. To account for this dynamic structure in our analysis, we included only the municipalities officially incorporated into each metropolitan area from the year in which they were formally established by state complementary law [20].

### Variables

The crude and age-specific incidence rates of PTB were calculated by dividing the total number of reported cases during the study period by the resident population over the same interval, and expressed per 100,000 population. To ensure comparability of the indicators, we applied age-specific weights to the crude rates based on the age distribution of the WHO World Standard Population [22], and calculated the age-standardized incidence rate (ASIR) using the direct method, as follows:

1 $$ASIR_{i} = \frac{{\mathop \sum \nolimits_{j = 1}^{N} \alpha_{ij} P_{j} }}{{\mathop \sum \nolimits_{j = 1}^{N} P_{j} }} \times 100,000$$

where *N* represents the total number of 5-year age groups, *i* denotes the *i*-th age group, $\alpha_{i}$ is the age-specific incidence rate for that group, and $P_{i}$ refers to the number of individuals in the standard population for the corresponding age group [23]. The ASIR was computed at the national level and separately for metropolitan and non-metropolitan regions, stratified by sex and by 5-year age groups.

We also included sociodemographic and clinical information. Records with missing data on sex (*n* = 240; 0.02%), age (*n* = 454; 0.04%), or municipality of residence (*n* = 3874; 0.31%) were proportionally redistributed according to the distribution of cases with complete information.

### Statistical analysis

We applied a set of complementary statistical approaches designed to characterize, quantify, and project the observed temporal trends. The following subsections present these methods.

### Joinpoint regression analysis

We used the Joinpoint Regression Program (version 5.4.0, National Cancer Institute, Bethesda, MD, USA) [24] to assess temporal trends in PTB incidence rates, stratified by area type, sex, age group, and period. The dependent variable was the natural logarithm of the incidence rate, and the independent variable was the year of diagnosis. The Joinpoint regression model fits a series of linear segments separated by *k* joinpoints (*τ₁*,…,*τₖ*), according to the following equation [25]:

2 $${\text{ln}}\left( {y_{t} } \right) = \beta_{0} + \beta_{1} t + \mathop \sum \limits_{j = 1}^{k} \delta_{j} \left( {t - \tau_{j} } \right)^{ + }$$

where *y*_*t*_ is the incidence rate in year *t*, defined either as the ASIR or as the age-specific incidence rate when stratified by 5-year age group. The *β*_*0*_ is the intercept, *β*_*1*_ is the slope of the first segment, and *δ*_*j*_ represents the change in slope after the joinpoint *τ*_*j*_. The function (*t* − *τ*_*j*_)^+^ = max(0, *t* − *τ*_*j*_) ensures that each slope change occurs only after its respective joinpoint.

Based on the estimated coefficients, we calculated the annual percent change (APC) and its 95% confidence interval (95% CI) using the formula: {*e*^*β*^ – 1} × 100%, where *β* is the regression coefficient for each segment. The average annual percent change (AAPC) was then computed as the weighted average of the APCs, using the length of each segment (*w*_*i*_, in years) as the weight: {$e^{{\left( {\sum w_{i} \beta_{i} /\sum w_{i} } \right)}}$– 1} × 100% [25].

This model allows the trend for each indicator to be classified as increasing, decreasing, or stable, and identifies the years in which significant changes in trend occurred. For all significance tests, we used the Monte Carlo permutation method (4499 permutations), accounting for first-order autocorrelated errors, and adopted a 5% significance level.

### Age-period-cohort model

We used the age-period-cohort model to examine the effects of age, period, and birth cohort on the incidence rate of PTB, stratified by sex and area. To meet the requirement of equal intervals across each dimension, the variables were grouped into 5-year intervals, resulting in 17 age groups (0–4; 5–9;…; 75–79; ≥ 80 years), four time periods (2001–2005; 2006–2010; 2011–2015; 2016–2020), and 20 birth cohorts (1921–1925; 1926–1930;…; 2011–2015; 2016–2020). Assuming that the observed number of PTB cases follows a Poisson distribution, and that the effects of age, period, and cohort act multiplicatively on the expected number of cases, the model was specified as follows [12]:

3 $$\begin{aligned} y_{ij} & \sim Poisson\left( {\lambda_{ij} P_{ij} } \right) \\ \ln \left( {\lambda_{ij} } \right) & = \mu + \alpha_{i} + \beta_{j} + \gamma_{k} , \\ \end{aligned}$$

where $y_{ij}$ denotes the observed number of PTB cases in age group *i* = 1,…, *I*, period *j* = 1,…, *J*; $P_{ij}$ represents the corresponding population at risk in the same stratum; and $\lambda_{ij}$ is the expected incidence rate. The natural logarithm is denoted by *ln*. The parameter *μ* is the overall intercept; *α*_*i*_ denotes the age effect, reflecting biological changes associated with aging; *β*_*j*_ represents the period effect, capturing contextual influences such as changes in diagnosis, treatment, socioeconomic conditions, or health policies; and *γ*_*k*_ refers to the cohort effect, associated with exposures shared by individuals born in the same time interval, such as the introduction of Bacillus Calmette-Guérin (BCG) vaccination. The cohort index *k* is deterministically defined from age and period as *k* = *I* – *i* + *j*, where *I* and *J* denote the total number of age and period categories, respectively [13].

To address the identification problem [period (*j*) – age (*i*) = cohort (*k*)], we constrained the sum of period effects to zero and used 2011–2015 as the reference period and the 1971–1975 cohort as the reference group. The modeling was employed using the *Epi* package [26], testing different parameterizations for each term and selecting the best-fitting model based on the lowest residual deviance [14].

Age effects were presented as age-specific incidence rates (per 100,000 population), adjusted for the reference period and cohort, while period and cohort effects were expressed as relative risks (RRs) compared to their respective reference groups [14]. Effects were considered statistically significant when the 95% confidence interval (*CI*) did not include the null value.

### Decomposition analysis

To investigate the factors contributing to differences in PTB incidence, we applied decomposition analysis using the *DasGuptaR* package [27]. This method quantifies the absolute and relative contributions of three key components to changes in disease burden: aging (shifts in the population’s age distribution), population growth (changes in total population size), and epidemiological changes (variations in age-specific incidence rates independent of demographic changes). In addition to estimating the main effects of each component, the method also calculates two- and three-way interaction effects, which capture how these factors act together rather than independently [28].

The decomposition analysis was performed using 2001 as the reference year and comparing it with each subsequent year from 2002 to 2020, based on three components. Absolute contributions were defined as the change in the number of PTB cases attributable to each component relative to 2001. Relative contributions were calculated as the proportion of cases attributable to each component divided by the total change in cases, expressed as a percentage. Positive values indicate a contribution to an increase in cases, whereas negative values indicate a reduction attributable to that factor [28].

### Bayesian age-period-cohort model

We employed a Bayesian age-period-cohort (BAPC) analysis using the Integrated Nested Laplace Approximation (INLA) method to estimate PTB incidence rates in Brazil from 2020 to 2035 [15, 29]. We based our projections on prospective population data by age group, sex, and municipality, provided by IBGE through 2024, and complemented them with projections adjusted using ARIMA models for the subsequent years using the *forecast* package [30].

Unlike classical methods, the Bayesian approach does not rely on rigid parametric assumptions and is currently one of the few strategies capable of producing realistic and non-arbitrary projections [15]. In the BAPC models, we applied smoothing priors to the age, period, and cohort effects, typically defined as Gaussian Markov random fields (GMRF) with first-order (RW1) or second-order (RW2) random walk structures, reflecting the assumption that adjacent time levels vary gradually. Mathematically, the prior for the vector of effects *α* is defined as [31]:

4 $$f\left( {\alpha {|}k_{{\varvec{\alpha}}} } \right) \propto k_{\alpha }^{{\frac{{\left( {I - r} \right)}}{2}}} exp^{{\left( { - \frac{1}{2}{\varvec{\alpha}}^{T} Q_{r} {\varvec{\alpha}}} \right)}}$$

where *k*_*α*_ is the precision parameter, *I* is the number of categories (e.g., age groups), and *Q*_*r*_ is the precision matrix associated with the finite difference operator of order *r*. In the case of RW1 (*r* = 1), corresponds to the sum of squared first-order differences: $\mathop \sum \limits_{i = 2}^{I} \left( {\alpha_{i} - \alpha_{i - 1} } \right)^{2}$, whereas for RW2 (*r* = 2), we use the second-order difference operator: $\mathop \sum \limits_{i = 3}^{I} \left( {\alpha_{i} - 2\alpha_{i - 1} + \alpha_{i - 2} } \right)^{2}$. To ensure identifiability, given the null space introduced by *Q*_*r*_, we typically impose sum-to-zero constraints or apply anchoring strategies to eliminate non-identifiable constant or linear components.

We also considered an extension of the BAPC model to account for additional unstructured heterogeneity, which cannot be explained by the modeled effects but may reflect unknown or unobserved covariates [15]:

5 $$\ln \left( {\lambda_{ij} } \right) = \mu + \alpha_{i} + \beta_{j} + \gamma_{k} + z_{ij} ,$$

where $z_{ij}$ is a random heterogeneity term added to the linear predictor, assumed to follow a normal distribution z*ᵢⱼ* ∼ *N*(0, $\tau_{z}^{ - 1}$). The hyperprior of a Gamma distribution, $\tau \sim Gamma\left( {a,b} \right)$, was assumed for all precision parameters, with shape and rate parameters defined as follows: age effect a = 1, b = 0.5; period, cohort, and overdispersion effects a = 1, b = 0.0005. The intercept (μ) was assigned a non-informative Normal prior with large variance, allowing the overall incidence level to be primarily determined by the data: μ ∼ *N*(0, 10^6^).

In this context, to select the most appropriate model structure for each combination of sex and area, we conducted retrospective validation using 4-year projections. We split the dataset into training data (80%, 2001–2015) and testing data (20%, 2016–2019). We then fitted BAPC models combining RW1 and RW2 smoothing priors, with and without overdispersion, stratified by sex and area (Table S2).

Next, we assessed the predictive accuracy of the models using the mean absolute percentage error (MAPE), defined as MAPE = $\frac{1}{n}\mathop \sum \limits_{i = 1}^{n} \left| {\frac{{\hat{y}_{i} - y_{i} }}{{y_{i} }}} \right| \times 100\%$, where *y*_*i*_ is the observed value and $\hat{y}_{i}$ is the predicted value; and the root mean squared error (RMSE), calculated as RMSE = $\sqrt {\frac{1}{n}\mathop \sum \limits_{i = 1}^{n} \left( {y_{i} - \hat{y}_{i} } \right)^{2} }$ [16, 17]. We selected the model structure with the lowest MAPE and RMSE values (Table S2).

Finally, we refit the selected model to the full dataset from 2001 to 2019 and used it for projection purposes. Predicted values were compared with observed data over the same period, stratified by sex and area type, as part of model validation (Fig. S1). We report median values with 95% credible intervals (CrI) (i.e., 2.5th and 97.5th percentiles) obtained from the posterior distributions of the BAPC models. It is important to note that, due to potential underreporting during the COVID-19 pandemic, observed data for the year 2020 were excluded from the validation stage and subsequently projected [11, 32].

Summary estimates of all variance parameters from the BAPC models are presented in Tables S3 and S4. These analysis were performed using the *BAPC* [33] and *INLA* packages [29].

All statistical analyses for epidemiological modeling and decomposition were implemented in R (version 4.4.2; R Foundation for Statistical Computing, Vienna, Austria) [34], and all data visualizations and graphical representations were created using the *ggplot2* package [35].

## Results

### Descriptive analysis

Between 2001 and 2020, a total of 1,262,968 new cases of PTB were reported in Brazil, with 685,866 (54.3%) occurring in metropolitan areas and 577,102 (45.7%) in non-metropolitan areas. In metropolitan areas, 460,946 cases (67.2%) were reported in men and 224,920 (32.8%) in women, with most cases occurring after 2011 (59.4%) and the highest concentration in the Southeast region (49.0%). The median age was 36.0 years [interquartile range (IQR): 25.0–50.0]. In non-metropolitan areas, 389,998 cases (67.6%) were reported in men and 187,104 (32.4%) in women, with most cases occurring before 2011 (62.3%) and predominance in the North and Northeast regions (42.6%). The median age was 38.0 years (IQR: 27.0–52.0). Additional information is available in Table S5.

### Temporal trend analysis

**Fig. 2:**
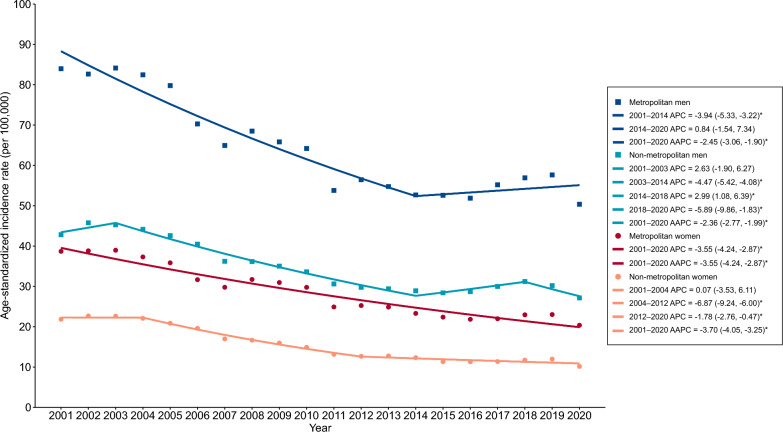
Temporal trends in age-standardized incidence rates of pulmonary tuberculosis by sex and areas in Brazil, 2001–2020. Values in parentheses represent 95% *CI*s. APC, annual percentage change; AAPC, average annual percent change; *CI*, confidence interval. ^*^ Statistically significant (*P*-value < 0.05)

Figure 2 and Table S6 present the results of the temporal trend analysis of the ASIR of PTB in Brazil by sex, including estimates for metropolitan and non-metropolitan areas, as well as age-specific rates. Between 2001 and 2020, the national ASIR was 32.8 cases per 100,000 population, declining from 39.4 in 2001 to 27.1 in 2020, with a downward trend over the period (AAPC = − 1.98; 95% *CI* − 2.56, − 1.39). In metropolitan areas, the ASIR was 45.4 per 100,000 population, higher than the national average, and declined from 59.5 in 2001 to 34.7 in 2020 (AAPC = − 2.67; 95% *CI* − 3.43, − 2.08). This decreasing trend was more pronounced among women (AAPC = − 3.55; 95% *CI* − 4.24, − 2.87) than among men (AAPC = − 2.45; 95% *CI* − 3.06, − 1.90). Across age groups, we observed reductions in all categories except for the 20–24 age group, which remained stable throughout most of the period, but showed an increasing trend from 2013 to 2020 (APC = 3.98; 95% *CI* 0.73, 14.12).

The five metropolitan areas with the highest ASIRs among men were Baixada Santista (118.5), Belém (103.2), Manaus (100.3), Rio de Janeiro (99.3), and Recife (99.1). An increasing trend was observed only in Belém (AAPC = 0.97; 95% *CI* 0.28, 1.66), while Rio de Janeiro showed a decline (AAPC = − 1.45; 95% *CI* − 1.81, − 1.10). Among women, the highest ASIRs were in Manaus (59.5), Belém (54.0), Baixada Santista (45.9), Rio de Janeiro (44.2), and Porto Velho (41.6), with decreasing trends in Baixada Santista (AAPC = − 0.85; 95% *CI* − 1.32, − 0.39), Rio de Janeiro (AAPC = − 2.66; 95% *CI* − 2.99, − 2.32), and Porto Velho (AAPC = − 4.22; 95% *CI* − 5.62, − 2.83). Additional results for other metropolitan areas are provided in Table S7.

In non-metropolitan areas, the ASIR was 25.0 per 100,000 population, decreasing from 32.0 in 2001 to 18.5 in 2020 (AAPC = − 2.54; 95% *CI* − 2.92, − 2.16). The decline was more pronounced among women (AAPC = − 3.70; 95% *CI* − 4.06, − 3.26) than among men (AAPC = − 2.36; 95% *CI* − 2.77, − 1.99), and consistent across all age groups, ranging from − 1.31% (95% *CI* − 1.92, − 0.80) in the 20–24 age group to − 5.26% (95% *CI* − 6.94, − 3.80) in the 5–9 age group (Fig. 2, Table S6).

The non-metropolitan areas of Roraima, Amazonas, Acre, and Amapá, states in the North region, had the highest ASIRs for both sexes. Among men, decreasing trends were observed only in Amapá (AAPC = − 2.66; 95% *CI* − 4.71, − 0.61) and Amazonas (AAPC = − 2.17; 95% *CI* − 3.04, − 1.30). Among women, declines were identified in Acre (AAPC = − 2.52; 95% *CI* − 3.86, − 1.17), Amapá (AAPC = − 4.77; 95% *CI* − 7.44, − 2.10), and Amazonas (AAPC = − 3.16; 95% *CI* − 4.11, − 2.22). More information for non-metropolitan areas are available in Table S7.

### Age-period-cohort analysis

Figs S2–S5 present descriptive plots of incidence rates by age, period, and birth cohort, allowing us to explore age-specific trends and suggest potential patterns related to cohort effects or temporal changes. Regarding the analysis of age, period, and birth cohort effects, for both sexes and area types, the model including all three effects simultaneously provided the best fit, as indicated by the lowest residual deviance and statistical significance (*P* < 0.001) (Table S8).

### Age effect

**Fig. 3:**
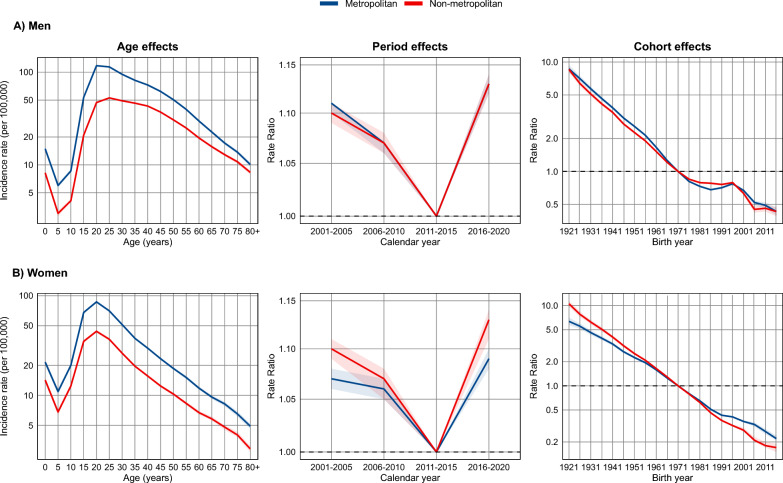
Age, period, and cohort effects on pulmonary tuberculosis incidence in Brazil, 2001–2020. **A** Men; **B** Women

Figure 3 and Table S9 show the estimated effect of age on PTB incidence rates, adjusted for period and cohort effects. We observed an initial decline up to the 5–9 age group, followed by a progressive increase peaking at 20–24 years for both sexes and area types, except among men in non-metropolitan areas, where the peak occurred at 25–29 years. From that point on, incidence rates gradually decreased with advancing age, with a sharper decline among women after age 65 in both area types. When comparing geographic contexts, we noted that incidence rates were consistently lower among residents of non-metropolitan areas across all age groups and both sexes, compared to those in metropolitan areas.

### Period effect

The period effect, presented in Fig. 3 and Table S9, was estimated after adjusting for age and cohort effects, using 2011–2015 as the reference period (RR = 1). We observed a general decline in risk up to the reference period, followed by an increase in both sexes and area types. The relative risk was similar among men (RR = 1.13; 95% *CI* 1.12–1.14) and women in non-metropolitan areas (RR = 1.13; 95% *CI* 1.11–1.14), and slightly lower among women in metropolitan areas (RR = 1.09; 95% *CI* 1.08–1.10). These findings are consistent with the overall trend identified in the Joinpoint regression analysis.

### Cohort effect

The analysis of cohort effects, adjusted for age and period, showed a progressive decline in the relative risk of PTB in more recent birth cohorts, with a consistent pattern across sexes and area types (Fig. 3, Table S9).

Among men in metropolitan areas, the highest risk was observed in the 1921–1925 cohort (RR = 8.65; 95% *CI* 7.95–9.40), followed by a continuous decline in subsequent cohorts, reaching an RR of 0.43 (95% *CI* 0.40–0.47) in the 2016–2020 cohort. A similar pattern was found among men in non-metropolitan areas, with an RR of 8.55 (95% *CI* 8.04–9.11) in the earliest cohort and 0.43 (95% *CI* 0.39–0.47) in the most recent (Fig. 3, Table S9).

Among women, the decline was also evident. In metropolitan areas, the RR dropped from 6.35 (95% *CI* 5.71–7.06) in the 1921–1925 cohort to 0.22 (95% *CI* 0.21–0.24) in 2016–2020. In non-metropolitan areas, we observed the highest RR among all groups in the 1921–1925 cohort (RR = 10.51; 95% *CI* 9.65–11.46), followed by the largest reduction, with RR reaching 0.17 (95% *CI* 0.15–0.19) in the most recent cohort (Fig. 3, Table S9).

### Decomposition analysis

**Fig. 4:**
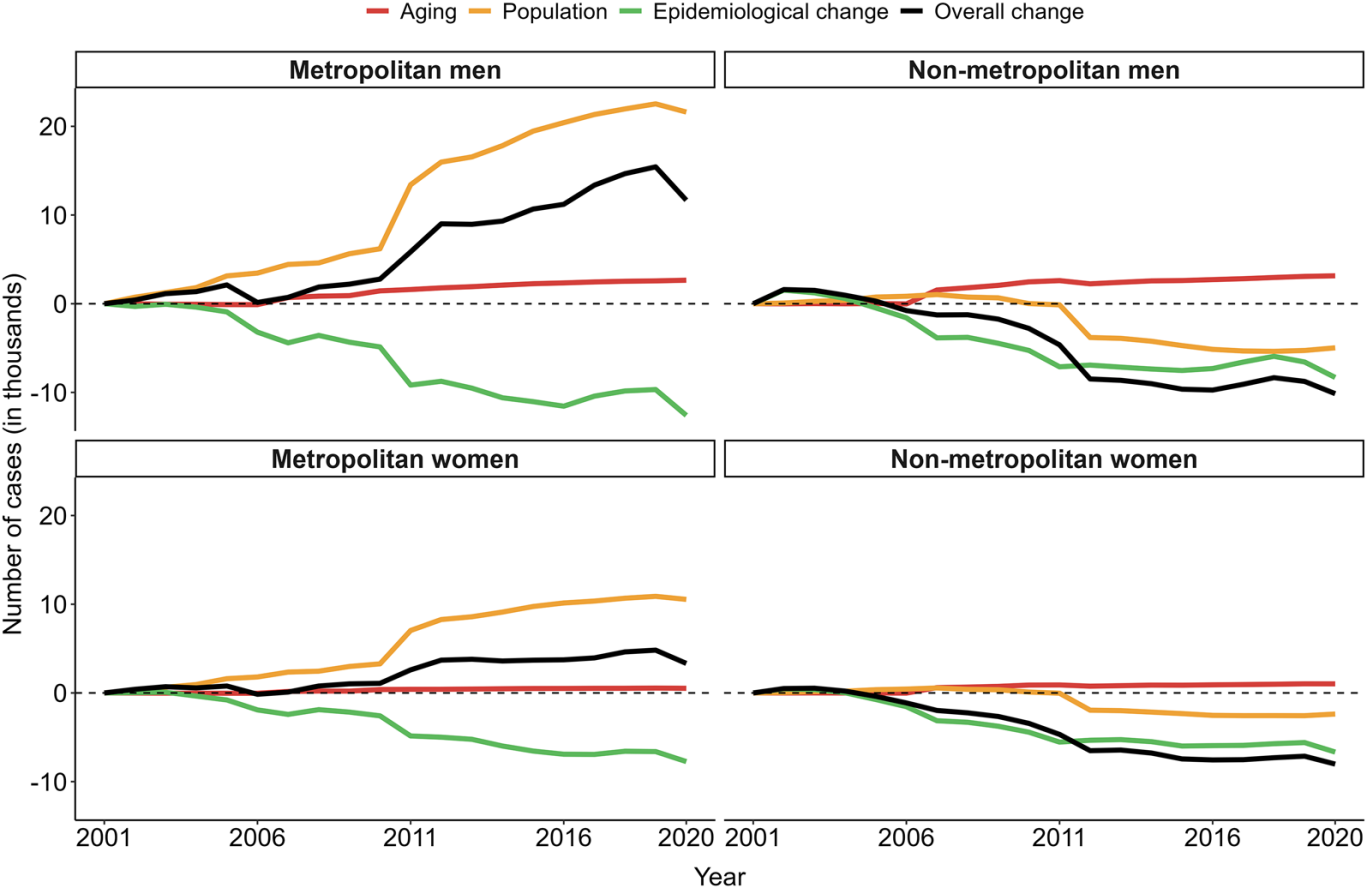
Changes in the number of pulmonary tuberculosis cases associated with population aging, population growth, and incidence rate changes from 2001 to 2020 in Brazil

Compared to 2001, the absolute number of PTB cases increased in metropolitan areas and decreased in non-metropolitan areas. To understand the factors underlying these changes, we decomposed the variation in case numbers into three components: aging, population growth, and epidemiological changes (Fig. 4).

Among men, the number of cases increased in metropolitan areas, driven primarily by population growth (21,610 cases, + 185.2%) and aging (2649 cases, + 22.7%). Although epidemiological changes contributed to a reduction of 12,588 cases (− 107.9%), this effect was not sufficient to offset the demographic impact. In contrast, non-metropolitan areas experienced a net reduction in cases, largely explained by epidemiological changes (− 8314 cases, − 82.0%) and population decline (− 4972 cases, − 49.0%). Population aging contributed positively (3147 cases) but was insufficient to reverse the overall downward trend (Fig. 4, Table S10).

Among women, a similar pattern was observed. In metropolitan areas, the total number of cases increased between 2001 and 2020, primarily due to population growth (10,545 cases, + 316.8%) and aging (521 cases, + 15.7%). Meanwhile, epidemiological changes resulted in a reduction of 7738 cases (− 232.4%). In non-metropolitan areas, the trend was downward, with a strong negative contribution from epidemiological changes (− 6663 cases, − 83.1%) and population decline (− 2380 cases, − 29.7%), which were not offset by the increase related to aging (1023 cases) (Fig. 4, Table S10).

The full decomposition results, stratified by sex and disaggregated by metropolitan and non-metropolitan areas, are presented in Table S11.

### Projection of future pulmonary tuberculosis incidence rates until 2035

**Fig. 5:**
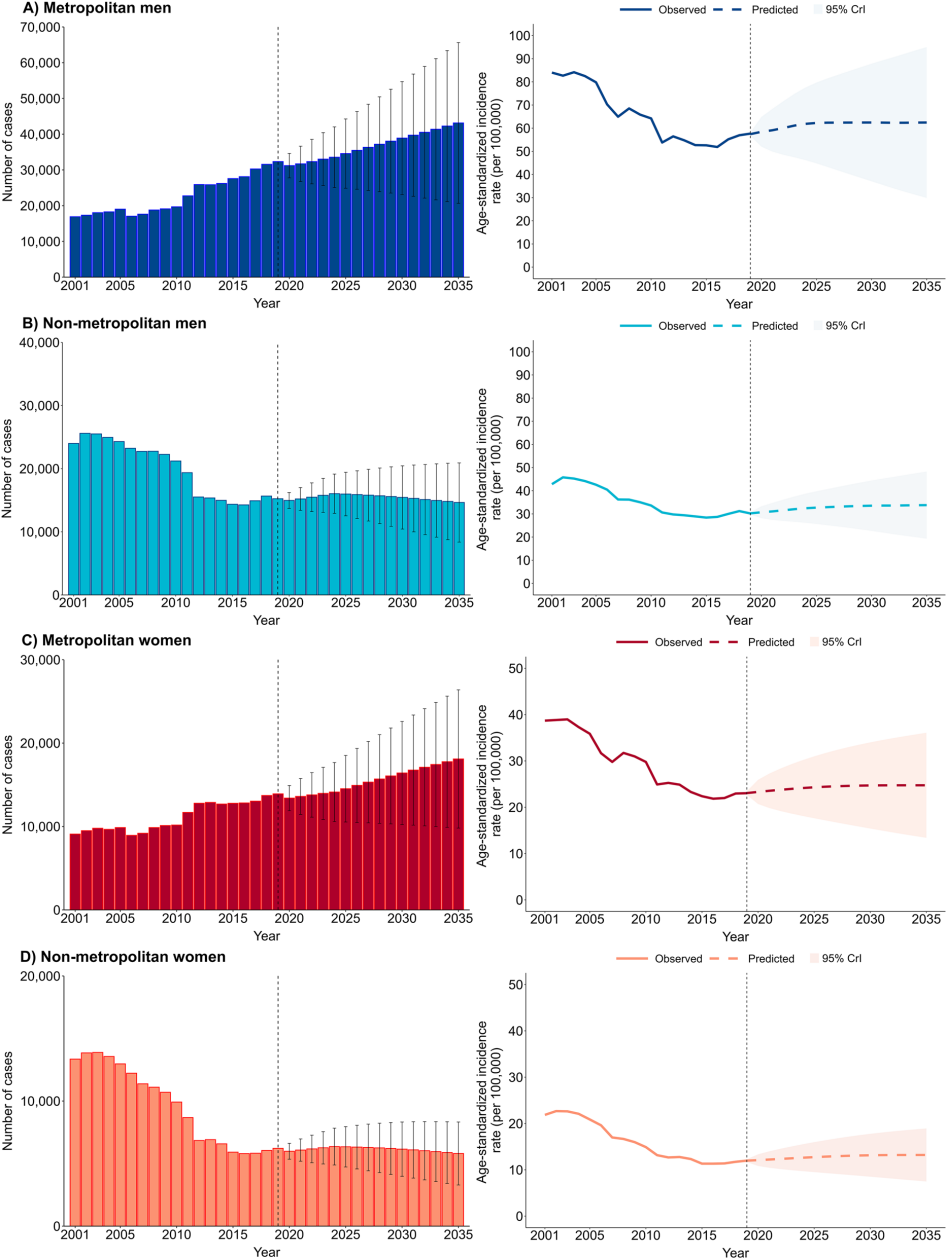
Projections of pulmonary tuberculosis cases and age-standardized incidence rates by sex and area in Brazil, 2001–2035. **A** Metropolitan men; **B** Non-metropolitan men; **C** Metropolitan women; **D** Non-metropolitan women. 95% CrI, 95% credible interval

Projections derived from the BAPC model, which combined retrospective data (2001–2019) and prospective population estimates, suggested heterogeneous trends in PTB incidence across regions, sexes, and age groups. Overall, the forecasts indicate a probable rise in PTB incidence rates in metropolitan areas, particularly among men, and a gradual decline or stabilization at relatively high levels in non-metropolitan regions. Detailed results, including projections of absolute case numbers and ASIR by area and sex, are presented in Fig. 5, with complementary stratified estimates by age group shown in Figs. S6 to S10.

### Projections in relation to elimination targets by 2035

According to the target milestones established by the WHO, the ASIR among men in metropolitan areas was 52.6 per 100,000 population in 2015. Projections indicate a gradual increase, reaching 62.3 (95% CrI: 44.8–79.7) in 2025 and remaining nearly unchanged at 62.4 (95% CrI: 29.9–95.0) through 2035, representing an overall change of + 18.6% (range: − 43.2% to + 80.6%) compared with 2015. In non-metropolitan areas, ASIR among men was 28.4 in 2015 and is projected to reach 33.8 (95% CrI: 19.3–48.3) by 2035. These results indicate relative stability with a slight upward tendency, corresponding to an estimated change of + 19.0% (range: − 32.0% to + 70.1%). Although the uncertainty intervals include the possibility of reduction, even the most favorable scenarios would remain far below the WHO’s elimination goals, which call for 80% and 90% reductions in incidence by 2030 and 2035, respectively (Table 1).

**Table 1:** Pulmonary tuberculosis cases and age-standardized incidence rates (per 100,000) observed in 2015 and projected for 2020, 2025, 2030, and 2035 with corresponding percentage changes, by sex and area in Brazil using Bayesian age-period-cohort models

Area	Men			Women		
	Cases (95% CrI)	ASIR (95% CrI)	% Change (range)^a^	Cases (95% CrI)	ASIR (95% CrI)	% Change (range)^a^
Metropolitan						
2015	27,577	52.6	–	12,782	22.4	–
2020	31,203 (27,776–34,630)	58.4 (52.0–64.8)	11 (− 1.1, 23.2)	13,404 (11,891–14,917)	23.3 (20.7–25.9)	4.0 (− 7.6, 15.6)
2025	34,571 (24,882–44,259)	62.3 (44.8–79.7)	18.4 (− 14.8, 51.5)	14,531 (10,537–18,524)	24.3 (17.6–31)	8.5 (− 21.4, 38.4)
2030	38,899 (23,091–54,706)	62.4 (37.1–87.8)	18.6 (− 29.5, 66.9)	16,417 (10,236–22,597)	24.7 (15.4–34)	10.3 (− 31.2, 51.8)
2035	43,140 (20,653–65,627)	62.4 (29.9–95.0)	18.6 (− 43.2,80.6)	18,098 (9807–26,390)	24.7 (13.4–36.1)	10.3 (− 40.2, 61.2)
Non-metropolitan						
2015	14,359	28.4	–	5913	11.3	–
2020	14,962 (13,700–16,223)	30.7 (28.2–33.2)	8.1 (− 0.7, 16.9)	5986 (5350–6622)	12.1 (10.8–13.3)	7.1 (− 4.4, 17.7)
2025	15,977 (12,529–19,426)	32.7 (25.7–39.8)	15.1 (− 9.5, 40.1)	6343 (4730–7956)	12.7 (9.5–16.0)	12.4 (− 15.9, 41.6)
2030	15,443 (10,432–20,454)	33.5 (22.7–44.4)	18.0 (− 20.1, 56.3)	6155 (3987–8324)	13.1 (8.5–17.8)	15.9 (− 24.8, 57.5)
2035	14,641 (8371–20,912)	33.8 (19.3–48.3)	19.0 (− 32.0, 70.1)	5808 (3290–8325)	13.2 (7.5–18.9)	16.8 (− 33.6, 67.3)

The End TB Strategy targets were defined as reductions in tuberculosis incidence of 20% by 2020, 50% by 2025, 80% by 2030, and 90% by 2035, relative to the 2015 baseline incidence rate. The point estimates (95% CrIs) were derived from the median values (2.5th and 97.5th percentiles) obtained from the Bayesian age–period–cohort models. The range represent estimated percentage variations, calculated from 95% CrIs of the projected values for each year relative to the 2015 baseline incidence rate

CrI, credible interval; ASIR, age-standardized incidence rate; –: not applicable

^a^Change in ASIR relative to the year 2015 = $\frac{{\left( {ASIR_{year} - ASIR_{2015} } \right)}}{{ASIR_{2015} }} \times 100\%$

Among women in metropolitan areas, ASIR is expected to remain largely stable, from 22.4 in 2015 to 24.7 (95% CrI: 13.4–36.1) in 2035, an estimated change of + 10.3% (range: − 40.2% to + 61.2%). In non-metropolitan areas, a slight increase is projected, from 11.3 in 2015 to 13.2 (95% CrI: 7.5–18.9) in 2035, representing an overall change of + 16.8% (range: − 33.6% to + 67.3%). Even considering the lower bounds suggesting possible declines, such reductions would remain far from sufficient to meet the global TB elimination targets (Table 1).

Among men, the highest projected ASIRs for 2035 in metropolitan areas are expected, particularly in Belém (177.8; 95% CrI: 64.1–291.6), Recife (103.5; 95% CrI: 39.6–167.4), Rio de Janeiro (102.9; 95% CrI: 52.1–153.6), and Porto Alegre (98.8; 95% CrI: 48.7–149.9). In non-metropolitan areas, the highest projected ASIRs are anticipated in the states of Acre (85.7; 95% CrI: 25.6–145.8), Rio de Janeiro (72.3; 95% CrI: 15.9–128.6), Roraima (60.2; 95% CrI: 29.7–90.8), Rio Grande do Sul (58.9; 95% CrI: 21.6–96.5), and Amazonas (54.2; 95% CrI: 17.7–90.7). Although the CrIs are relatively wide, reflecting uncertainty in long-term projections, the central estimates and even most lower bounds remain high. Complete results, stratified by sex and area type, are available in Table S12.

## Discussion

Our findings, obtained through a distinct methodological approach, confirm previous evidence that Brazil is unlikely to achieve the TB control targets [3, 4, 11]. Beyond reinforcing this scenario, our study advances understanding by examining PTB incidence disaggregated by metropolitan and non-metropolitan areas, thereby revealing regional disparities often masked in national-level analyses. Using data from 2001 to 2019, we applied BAPC models to project case counts and age-standardized incidence rates through 2035. Furthermore, decomposition analysis added depth to these trends by quantifying the relative contributions of population aging, demographic growth, and changes in age-specific rates to the variation in case numbers over time.

During the study period, we observed an average annual decrease of approximately 2% (95% *CI* − 2.56, − 1.39) in the ASIR in Brazil [36–38], with a more pronounced decline from 2001 to 2014, an era marked by economic expansion, the strengthening of social policies, the expansion of primary care, and increased investment in the Unified Health System (SUS) [39]. However, from 2014 onward, this downward trend reversed, with rising incidence among young adults (20–24 years) in metropolitan areas and among men in non-metropolitan areas. This resurgence may be associated with the austerity policies implemented in 2015, which resulted in rising unemployment, cuts to social programs, and reduced SUS funding. These measures disproportionately affected vulnerable populations and further undermined the system’s capacity to respond effectively to TB control [40, 41].

Our projections indicated that, by 2035, PTB incidence in Brazil will remain above the WHO’s elimination target (< 10 cases per 100,000 population) [1], particularly in metropolitan areas, where the largest increases are projected. In contrast, non-metropolitan areas are expected to stabilize, although at levels that remain above the targets set by the WHO. Decomposition analysis reinforces these findings by demonstrating that, although population aging contributed to the absolute increase in cases, especially among men, population growth emerged as the primary driver of rising trends, particularly in vulnerable urban settings.

The projections also reveal marked disparities by sex. Among men, a significant increase in incidence is expected nationwide, reflecting structural inequalities previously reported, such as longer infectious periods, lower adherence to diagnosis and treatment, and higher exposure to risk factors [42]. Among women, a more moderate increase is expected, with non-metropolitan residents more likely to reach incidence rates below 20 per 100,000 by 2030 [1].

Special attention should be directed to areas with high projected rates. Among men, the highest ASIRs by 2035 are expected in the metropolitan areas of Belém, Recife, and Rio de Janeiro, which may each exceed 100 per 100,000. In non-metropolitan areas, high rates are also projected in the states of Acre, Rio de Janeiro, Roraima, Rio Grande do Sul, and Amazonas. Although uncertainty in long-term projections, the overall trajectory remains concerning, indicating that TB incidence is unlikely to decline substantially under current conditions. These findings highlight the need for targeted regional strategies, particularly those focused on male populations and high-risk territories [7, 43].

These disparities are consistent with existing literature, which identifies men as the primary transmitters of TB, impacting women and children [44]. Social and behavioral barriers to accessing health care worsen this scenario, particularly in large urban centers [6, 7, 9]. In this context, TB control policies must systematically strengthen the incorporation of gender considerations and territorial specificities into national recommendations, establishing them as a reference for defining the most appropriate control strategies for each territory [4, 45]. Strategies specifically targeting men, especially in metropolitan areas, will be essential to reducing transmission, and moving Brazil closer to global elimination goals.

Previous national studies support the projections presented in this analysis. One study integrating three national datasets using a Bayesian Structural Time Series model predicted that, even under current TB control policies, Brazil’s incidence will remain above WHO targets by 2030 (42.1 per 100,000) [3]. This persistence has been attributed to ongoing challenges such as unequal access to health services, the prolonged effects of the COVID-19 pandemic, and chronic underfunding of TB control efforts [3]. Another recent study, applying SARIMA models to forecast monthly incidence through 2030, estimated that more than 124,000 cases may occur that year, reaching levels similar to those observed in the early 2000s [11]. Furthermore, an APC-based study using 2002–2019 data projected rising incidence through 2034, especially among men, and a slight reduction in mortality among women, linked to lower risk in this group [4]. Together, these findings strengthen the robustness of our projections and underscore the need for more effective, sustained, and equity-oriented policies.

The analysis of age effects showed the lowest incidence rates among children aged 5–9 years, likely due to neonatal BCG vaccination introduced into the National Immunization Program (PNI) in 1977 [46]. Peak incidence occurred among young adults (20–29 years), likely reflecting increased social mobility and exposure in high-risk environments, as well as waning BCG-induced immunity over time [46, 47]. After age 30, a decline in rates was observed, differing from the pattern seen in countries such as China, the United States, and India [48], a trend that may reflect improvements in social determinants of health and expanded access to primary care in Brazil [49].

Incidence rates were consistently higher among men across all age groups, attributable to greater occupational exposure, tobacco and alcohol use, and lower engagement with care [50]. In addition, men show higher prevalence of latent TB infection (LTBI), which increases the likelihood of progression to active disease [51] and may partially explain the stronger age effect observed. Among men, these factors were more pronounced in metropolitan areas than in non-metropolitan areas, where structural vulnerabilities such as overcrowding, social inequality, and limited access to health services may further amplify both transmission and reactivation risks [7, 8, 45].

The period effect was consistent with Joinpoint trend analysis, indicating declining rates in the early years, stabilization from 2006 to 2010, and increases from 2016 onward, possibly linked to setbacks in social policy, the economic crisis of 2015, and the effects of events such as the COVID-19 pandemic, which also caused a decrease in detection [32, 41]. These findings emphasize the importance of sustained and integrated public health policies and the challenges health managers face in maintaining progress achieved in earlier decades.

Cohort effect analysis revealed a progressive decline in the relative risk of PTB among more recent generations. From the reference cohort (1971–1975) onward, risk estimates remained consistently below 1.0, suggesting intergenerational gains linked to improvements in living conditions, access to health services, and public policies [52]. The 2016–2020 cohort showed the lowest risk levels, particularly among women. However, in older cohorts, women living in non-metropolitan areas retained higher risk levels compared with those in metropolitan areas, illustrating how long-standing social inequalities in access to health care, including timely diagnosis and treatment of TB, shaped vulnerability, particularly before the expansion of universal primary health care in Brazil [52]. Among men, TB incidence remained elevated across successive birth cohorts in both metropolitan and non-metropolitan contexts, indicating a long-standing pattern of increased disease burden that, although declining in more recent cohorts, has spanned generations [4].

Finally, although cohort effects point to accumulated progress, age and period effects have a more immediate impact on the dynamics of PTB. Our findings highlight the importance of combining long-term policy planning with effective strategies for prevention, screening, and treatment, sensitive to regional and sex-based differences, to curb the spread of the disease and achieve TB elimination targets in Brazil.

## Limitations and strengths

This study has several limitations. First, we relied on notification data from official surveillance systems, which are subject to underreporting and registration delays, especially in remote areas with limited surveillance capacity [53]. Nonetheless, the quality of the SINAN-TB system has improved substantially over the past decades, increasing its reliability [21]. We excluded the year 2020 from validation and projection analyses due to the exceptional impact of the COVID-19 pandemic on surveillance systems and potential data distortions [11, 32].

Second, the effects of PTB were assessed using aggregated data, which may not accurately reflect individual-level associations and are subject to ecological fallacy. Third, we adopted the legally defined classification of metropolitan and non-metropolitan areas in Brazil. Although this definition may not fully capture recent socio-spatial changes, such as urban sprawl and intermunicipal flows, it remains a normative and widely used basis for territorial planning and public health policy management at the state and municipal levels.

Fourth, official municipal population projections were unavailable through 2035 at the required disaggregated level, necessitating the use of time-series modeling to estimate the population by municipality, sex, and age group, an approach subject to additional uncertainty. Finally, projections assume the continuation of historical risk patterns, which may not hold in the face of major structural, technological, or policy changes. These assumptions become progressively less realistic over longer projection horizons, particularly for the approximately 15-year period from 2020 to 2035, as unforeseen contextual shifts may alter future dynamics. Although BAPC models are robust, they require further refinement to improve accuracy in rapidly changing settings.

Despite these limitations, to our knowledge, this is the first study to use officially defined metropolitan areas as the analytical unit for PTB incidence projections in Brazil. This approach revealed regional disparities often masked in nationally aggregated analyses. Furthermore, we employed robust and complementary statistical methods to ensure a coherent interpretation of temporal patterns. Joinpoint regression identified significant inflection points in incidence trends, while the classical age-period-cohort model disentangled age, period, and cohort effects underlying these changes. Decomposition analysis quantified the relative contributions of demographic and epidemiological factors. Finally, the BAPC model extended this framework to project future trends while incorporating statistical uncertainty. Together, these methods enhance the precision and interpretability of our estimates and strengthen the study’s relevance for designing more targeted and effective public health policies.

## Recommendations and public health implications

Reducing the burden of TB in Brazil over the coming decades will require a comprehensive set of strategies tailored to the country’s epidemiological and territorial contexts. The projected increase in disease rates, especially in major urban centers and socially vulnerable areas, may be linked to the reactivation of latent infections and ongoing transmission of *Mycobacterium* tuberculosis [6, 51]. In this scenario, it is essential to expand the use of rapid molecular diagnostic tools, such as Xpert MTB/RIF Ultra [54], to strengthen early detection through active case finding, such as contact investigation, and latent infection screening, while also scaling up access to preventive treatment [55]. Coordinated implementation of these actions, with a focus on priority territories, can halt latent TB progression by expanding LTBI screening and treatment, while also preventing the spread of resistant strains through improved diagnosis, effective treatment, and infection control. Together, these measures contribute to reduced incidence and improved treatment outcomes [56]. These measures align with the WHO End TB Strategy target of an 80% reduction in TB incidence by 2030 through early diagnosis and preventive therapy [1].

Second, it is crucial to restore and stabilize TB prevention and control services following the disruptions caused by the COVID-19 pandemic [57]. Continuity of diagnosis and treatment efforts must be ensured, particularly in metropolitan areas with high TB burdens such as Belém, Rio de Janeiro, Recife, and Porto Alegre. Integrating TB surveillance into national pandemic preparedness and response systems, as recommended by the WHO [1], is a key strategy to strengthen health system resilience and mitigate the impact of future health emergencies.

Third, public health policies should be designed based on the regional and demographic specificities of TB. In metropolitan areas, efforts should prioritize younger populations and people experiencing homelessness, with a focus on reducing risk factors such as smoking, harmful alcohol use, illicit drug use, and comorbidities like diabetes mellitus [58]. In non-metropolitan areas, addressing structural and social barriers to timely healthcare access, particularly among Indigenous communities and migrant populations in the North region, is essential for improving diagnosis and treatment continuity [59].

Finally, in light of the persistent gap between stated goals and the epidemiological reality, it is urgent to increase investment in cost-effective TB prevention and treatment strategies, especially in regions with limited infrastructure [57]. These measures should enhance local capacity for surveillance, diagnosis, and care, promoting greater equity in TB control.

## Conclusions

Using a different methodological approach, our findings corroborate previous analyses indicating that, even in the most favorable scenarios, Brazil is unlikely to achieve national and international TB elimination targets by 2035, as outlined by the WHO. The epidemiological outlook suggests two likely trajectories: a projected increase in PTB incidence in metropolitan areas and a stabilization at high levels in non-metropolitan areas. Importantly, by decomposing the trends, our analysis demonstrates that these apparently similar outcomes arise from distinct underlying drivers. In metropolitan regions, population growth and aging explain the rising number of cases, while in non-metropolitan areas, epidemiological improvements and, to a lesser extent, population decline contributed to the observed reduction.

These results underscore the urgent need to strengthen TB control policies, ensuring they are more effective, equitable, and tailored to local contexts. Addressing the heterogeneous demographic and epidemiological dynamics across regions is crucial for developing targeted strategies for vulnerable populations, particularly men living in large urban centers. Furthermore, future research should refine our projections by incorporating determinants such as the proportion of multi-drug-resistant TB and HIV coinfection, which may significantly influence future incidence patterns and the effectiveness of control interventions.

## Supplementary Information

Additional file 1.

## Data Availability

The data used in this study are open-access datasets, and their references are provided within the manuscript. Additional information is available from the corresponding author upon reasonable request.

## Abbreviations

AAPC: Average annual percent change
APC: Annual percent change
ARIMA: Autoregressive Integrated Moving Average
ASIR: Age-standardized incidence rate
BAPC: Bayesian age-period-cohort
BCG: Bacillus Calmette-Guérin
*CI*: Confidence interval
CrI: Credible interval
COVID-19: Coronavirus disease 2019
DATASUS: Department of Informatics of the Unified Health System
GMRF: Gaussian Markov random fields
IBGE: Brazilian Institute of Geography and Statistics
INLA: Integrated Nested Laplace Approximation
IQR: Interquartile range
LTBI: Latent tuberculosis infection
MAPE: Mean absolute percentage error
PNI: National Immunization Program
PTB: Pulmonary tuberculosis
RMSE: Root mean squared error
RR: Relative risk
RW: Random walk
SARIMA: Seasonal Autoregressive Integrated Moving Average
SINAN: Notifiable Diseases Information System
SUS: Unified Health System
TB: Tuberculosis
WHO: World Health Organization
