# Supplementary material for: Projecting tuberculosis control progress in metropolitan and non-metropolitan areas of Brazil, 2001–2035: a Bayesian age-period-cohort analysis
Source: Infect Dis Poverty. 2025 Dec 22;14:125. doi: 10.1186/s40249-025-01400-x (PMC12720469; doi:10.1186/s40249-025-01400-x)
Supplement: Supplementary file 1 — Additional file 1. [file 40249_2025_1400_MOESM1_ESM.docx]

**Supplemental Material**

**Projecting tuberculosis control progress in metropolitan and non-metropolitan areas of Brazil, 2001–2035: a Bayesian age–period–cohort analysis**

**Table of Contents**

**Table S1**. Number of municipalities, and estimates of population size and aging rate for individuals by sex and area in Brazil, 2020…...………………………………………………..3

**Table S2.** Predictive performance of BAPC models by sex and area in Brazil………………...7

**Fig S1.** Validation of the Bayesian age-period-cohort model based on the comparison between observed and predicted pulmonary tuberculosis incidence rates, by sex and area, in Brazil, 2001–2019……………………………………………………………………………………...8

**Table S3.** The summary estimates of all variance parameters in the BAPC models by sex and area in Brazil, 2001-2019.……………………………………………………………………...9

**S4 Table.** Hyperparameter estimates (95% CrI) in the BAPC models by sex and each area in Brazil, 2001–2019…………………………………………………………………………….10

**Table S5.** Epidemiological characteristics of pulmonary tuberculosis cases by area in Brazil, 2001–2020…………………………………………………………………………………….15

**Table S6.** Temporal trends and age-standardized incidence rates of pulmonary tuberculosis in Brazil, 2001–2020…………………………………………………………………………….18

**Table S7.** Temporal trends and age-standardized incidence rates of pulmonary tuberculosis by sex and area described by state in Brazil, 2001–2020…………………………………………23

**Fig S2.** Pulmonary tuberculosis incidence rates by age group, connected within each period, stratified by sex and area in Brazil, 2001–2020………..………………………………………29

**Fig S3.** Pulmonary tuberculosis incidence rates by age group, connected within each birth cohort, stratified by sex and area in Brazil, 2001–2020…..……………………………………29

**Fig S4.** Pulmonary tuberculosis incidence rates by period, connected within age groups, stratified by sex and area in Brazil, 2001–2020………………………………………………..30

**Fig S5.** Pulmonary tuberculosis incidence rates by birth cohort, connected within age group, stratified by sex and area in Brazil, 2001–2020………………………………………………..30

**Table S8.** Model estimates for the age-period-cohort effect on pulmonary tuberculosis incidence, by sex and area in Brazil, 2001-2020……………………………………………....31

**Table S9.** Estimates of the age–period–cohort model adjusted for pulmonary tuberculosis rates by age group, including relative risks for period and birth cohort, by sex and area, Brazil, 2001–2020…………………………………………………………………………………………...32

**Table S10.** Changes in the number of pulmonary tuberculosis cases associated with aging, population growth, and epidemiological changes from 2001 to each year, by sex and area, using a decomposition method…………………………………………………………………..…..34

**Table S11.** Changes in the number of pulmonary tuberculosis cases attributed to aging, population growth, and epidemiological changes from 2001 to each year, by sex and area described by state, using a decomposition method…………………………………………….36

**Fig S6.** Age-specific predicted incidence rates of pulmonary tuberculosis among metropolitan men in Brazil, 2001–2035……………………………………………………………………..42

**Fig S7.** Age-specific predicted incidence rates of pulmonary tuberculosis among non-metropolitan men in Brazil, 2001–2035……………………………………………………....43

**Fig S8.** Age-specific predicted incidence rates of pulmonary tuberculosis among metropolitan women in Brazil, 2001–2035………………………………………………………………….44

**Fig S10.** Age-specific predicted incidence rates of pulmonary tuberculosis among non-metropolitan women in Brazil, 2001–2035…………………………………………………....45

**Table S12**. Pulmonary tuberculosis cases and age-standardized incidence rates (per 100,000) observed in 2015 and projected for 2020, 2025, 2030, and 2035, by sex and area described by state, using Bayesian age-period-cohort models……………………………………………....46

| **Table S1**. Number of municipalities, and estimates of population size and aging rate for individuals by sex and area in Brazil, 2020. | | | | | |
| --- | --- | --- | --- | --- | --- |
| **Region/State** |  | **Men** | | **Women** | |
|  | **Number of municipalities** | **Population size^a^** | **Aging rate (%)^b^** | **Population size^a^** | **Aging rate (%)^b^** |
| **Total** | **5570** | **107,023,935** | **13.0** | **102,140,954** | **15.6** |
| **North** | | | | | |
| Acre | | | | | |
| Non-metropolitan | 22 | 433,188 | 8.2 | 430,469 | 8.7 |
| Amapá | | | | | |
| Macapá | 3 | 304,324 | 7.0 | 309,236 | 8.0 |
| Non-metropolitan | 13 | 89,253 | 7.4 | 82,720 | 6.2 |
| Amazonas | | | | | |
| Manaus | 13 | 1,316,497 | 8.0 | 1,348,395 | 9.4 |
| Non-metropolitan | 49 | 756,388 | 7.3 | 699,946 | 7.0 |
| Pará | | | | | |
| Belém | 7 | 1,152,681 | 10.9 | 1,252,730 | 13.8 |
| Santarém | 3 | 188,388 | 9.5 | 190,697 | 10.3 |
| Non-metropolitan | 134 | 2,913,494 | 8.8 | 2,767,202 | 8.5 |
| Rondônia | | | | | |
| Porto Velho | 2 | 261,254 | 8.7 | 261,093 | 9.4 |
| Non-metropolitan | 50 | 608,041 | 11.5 | 592,365 | 11.4 |
| Roraima | | | | | |
| Capital | 5 | 245,842 | 7.2 | 243,873 | 7.5 |
| Central | 2 | 16,804 | 10.2 | 15,046 | 7.9 |
| Sul do Estado | 4 | 31,937 | 9.1 | 28,523 | 7.5 |
| Non-metropolitan | 4 | 30,612 | 5.6 | 28,338 | 5.1 |
| Tocantins | | | | | |
| Gurupi | 18 | 98,592 | 13.1 | 96,074 | 12.9 |
| Palmas | 16 | 245,094 | 9.2 | 248,013 | 9.3 |
| Non-metropolitan | 105 | 433,099 | 12.4 | 417,014 | 12.3 |
| **Northeast** | | | | | |
| Alagoas | | | | | |
| Agreste | 18 | 281,478 | 11.8 | 297,737 | 13.6 |
| Caetés | 2 | 51,604 | 10.2 | 55,212 | 11.3 |
| Maceió | 11 | 592,797 | 10.3 | 660,767 | 13.1 |
| Médio Sertão | 9 | 72,039 | 11.1 | 74,193 | 12.7 |
| Palmeira dos Índios | 3 | 16,657 | 13.2 | 17,258 | 14.7 |
| Sertão | 7 | 75,112 | 11.5 | 78,463 | 13.0 |
| São Francisco | 5 | 60,957 | 11.8 | 63,715 | 13.5 |
| Vale do Paraíba | 10 | 76,334 | 12.1 | 79,951 | 13.5 |
| Zona da Mata | 16 | 149,021 | 10.9 | 154,302 | 11.6 |
| Non-metropolitan | 21 | 176,162 | 11.0 | 181,064 | 11.9 |
| Bahia | | | | | |
| Feira de Santana | 16 | 450,354 | 11.1 | 497,632 | 14.1 |
| Salvador | 13 | 1,703,628 | 11.8 | 1,950,447 | 15.1 |
| Non-metropolitan | 388 | 5,032,097 | 13.2 | 5,114,393 | 14.8 |
| Ceará | | | | | |
| Cariri | 9 | 308,918 | 11.1 | 334,371 | 14.1 |
| Fortaleza | 19 | 1,935,665 | 10.8 | 2,129,288 | 14.0 |
| Sobral | 18 | 244,536 | 11.6 | 251,508 | 13.8 |
| Non-metropolitan | 138 | 1,928,618 | 13.8 | 1,956,025 | 15.4 |
| Maranhão | | | | | |
| Grande São Luís | 13 | 794,320 | 9.5 | 876,243 | 11.6 |
| Sudoeste Maranhense | 8 | 186,076 | 10.3 | 194,117 | 11.6 |
| Non-metropolitan | 196 | 2,464,235 | 11.0 | 2,455,997 | 11.8 |
| Paraíba | | | | | |
| Araruna | 6 | 30,672 | 14.4 | 31,306 | 16.8 |
| Barra de Santa Rosa | 8 | 38,579 | 15.6 | 39,682 | 18.0 |
| Cajazeiras | 15 | 87,307 | 14.6 | 90,108 | 17.5 |
| Campina Grande | 19 | 320,319 | 12.2 | 344,258 | 15.8 |
| Esperança | 9 | 68,753 | 13.3 | 71,738 | 16.4 |
| Guarabira | 20 | 122,657 | 13.8 | 127,571 | 16.7 |
| Itabaiana | 12 | 67,137 | 13.8 | 69,544 | 16.3 |
| João Pessoa | 12 | 628,554 | 11.0 | 696,995 | 14.4 |
| Patos | 23 | 111,072 | 13.0 | 118,446 | 16.0 |
| Sousa | 9 | 57,291 | 15.0 | 59,534 | 18.0 |
| Vale do Mamanguape | 9 | 61,072 | 12.0 | 62,119 | 13.9 |
| Vale do Piancó | 17 | 69,907 | 14.3 | 70,878 | 16.8 |
| Non-metropolitan | 64 | 305,410 | 14.3 | 314,026 | 16.7 |
| Pernambuco | | | | | |
| Recife | 15 | 1,888,771 | 12.0 | 2,127,414 | 16.3 |
| Non-metropolitan | 170 | 2,647,544 | 11.6 | 2,775,233 | 13.8 |
| Piauí | | | | | |
| Non-metropolitan | 224 | 1,634,070 | 12.8 | 1,701,793 | 14.8 |
| Rio Grande do Norte | | | | | |
| Natal | 15 | 752,738 | 11.3 | 818,304 | 14.7 |
| Non-metropolitan | 152 | 907,897 | 13.0 | 928,679 | 15.2 |
| Sergipe | | | | | |
| Aracaju | 4 | 445,115 | 10.0 | 502,066 | 13.1 |
| Non-metropolitan | 71 | 642,629 | 11.5 | 664,408 | 13.0 |
| **Southeast** | | | | | |
| Espírito Santo | | | | | |
| Grande Vitória | 7 | 949,883 | 12.6 | 1,014,361 | 15.6 |
| Non-metropolitan | 71 | 1,013,806 | 14.3 | 1,023,417 | 15.9 |
| Minas Gerais | | | | | |
| Belo Horizonte | 50 | 2,840,180 | 13.6 | 3,066,255 | 17.1 |
| Vale do Aço | 28 | 362,324 | 15.6 | 380,043 | 17.5 |
| Non-metropolitan | 775 | 7,124,383 | 15.5 | 7,258,824 | 17.7 |
| Rio de Janeiro | | | | | |
| Rio de Janeiro | 22 | 6,165,543 | 14.7 | 6,865,266 | 19.3 |
| Non-metropolitan | 70 | 2,029,803 | 15.3 | 2,161,693 | 17.8 |
| São Paulo | | | | | |
| Baixada Santista | 9 | 879,366 | 15.1 | 960,447 | 19.1 |
| Campinas | 20 | 1,571,889 | 13.5 | 1,648,236 | 16.4 |
| Ribeirão Preto | 34 | 820,469 | 14.0 | 857,695 | 17.2 |
| Sorocaba | 27 | 1,079,423 | 13.3 | 1,100,914 | 15.8 |
| São Paulo | 39 | 10,280,482 | 12.6 | 11,206,616 | 16.3 |
| Vale do Paraíba e Litoral Norte | 39 | 1,245,845 | 14.0 | 1,299,123 | 16.5 |
| Non-metropolitan | 477 | 6,203,439 | 15.3 | 6,369,247 | 18.2 |
| **South** | | | | | |
| Paraná | | | | | |
| Apucarana | 23 | 148,522 | 16.9 | 153,824 | 18.8 |
| Campo Mourão | 25 | 170,755 | 16.2 | 176,625 | 18.0 |
| Cascavel | 24 | 273,842 | 13.3 | 283,404 | 15.2 |
| Curitiba | 29 | 1,756,134 | 12.3 | 1,862,185 | 15.4 |
| Londrina | 25 | 533,432 | 15.3 | 569,929 | 18.2 |
| Maringá | 26 | 410,850 | 14.7 | 434,326 | 17.5 |
| Toledo | 18 | 204,349 | 14.5 | 211,517 | 16.6 |
| Umuarama | 24 | 161,254 | 16.3 | 165,611 | 18.4 |
| Non-metropolitan | 205 | 2,004,691 | 14.1 | 2,039,737 | 15.9 |
| Rio Grande do Sul | | | | | |
| Porto Alegre | 34 | 2,011,315 | 14.9 | 2,189,554 | 19.2 |
| Serra Gaúcha | 14 | 397,926 | 15.1 | 415,193 | 18.5 |
| Non-metropolitan | 449 | 3,042,443 | 18.0 | 3,163,855 | 21.0 |
| Santa Catarina | | | | | |
| Alto Vale do Itajaí | 28 | 154,841 | 14.1 | 153,552 | 16.2 |
| Carbonífera | 26 | 317,757 | 14.0 | 325,563 | 16.4 |
| Chapecó | 32 | 264,520 | 12.8 | 263,818 | 15.0 |
| Contestado | 45 | 271,415 | 14.5 | 272,005 | 16.7 |
| Extremo Oeste | 49 | 177,697 | 17.0 | 175,586 | 18.9 |
| Florianópolis | 22 | 647,600 | 12.6 | 680,556 | 15.6 |
| Foz do Rio Itajaí | 9 | 368,608 | 11.3 | 382,700 | 14.0 |
| Lages | 23 | 183,962 | 14.3 | 185,542 | 16.4 |
| Norte/Nordeste Catarinense | 26 | 734,056 | 12.1 | 740,264 | 14.5 |
| Tubarão | 19 | 200,463 | 15.5 | 205,596 | 18.2 |
| Vale do Itajaí | 16 | 413,285 | 12.2 | 422,589 | 15.1 |
| **Central-West** | | | | | |
| Distrito Federal | | | | | |
| Non-metropolitan | 1 | 1,398,532 | 10.0 | 1,522,885 | 12.7 |
| Goiás | | | | | |
| Goiânia | 21 | 1,266,220 | 11.0 | 1,342,246 | 13.6 |
| Non-metropolitan | 225 | 2,227,415 | 12.4 | 2,224,409 | 13.2 |
| Mato Grosso | | | | | |
| Vale do Rio Cuiabá | 13 | 538,061 | 11.3 | 552,116 | 12.8 |
| Non-metropolitan | 128 | 1,288,423 | 10.5 | 1,233,387 | 10.3 |
| Mato Grosso do Sul | | | | | |
| Non-metropolitan | 79 | 1,392,161 | 12.2 | 1,414,705 | 13.7 |

^a^ Population size was based on the population estimates provided by the Instituto Brasileiro de Geografia e Estatística (IBGE), disaggregated by sex, age group, and municipality.

^b^ Ageing rates were defined as the proportion of individuals aged 60 years and older relative to the total population aged 0 years and older.

**Table S2.** Predictive performance of BAPC models by sex and area in Brazil.

|  | **Effects** |  |  | **Metropolitan area** | | | | **Non-metropolitan area** | | | |
| --- | --- | --- | --- | --- | --- | --- | --- | --- | --- | --- | --- |
| **Age** | **Period** | **Cohort** | **OD** | **Men** | | **Women** | | **Men** | | **Women** | |
| **Prior** | **Prior** | **Prior** |  | **RMSE** | **MAPE** | **RMSE** | **MAPE** | **RMSE** | **MAPE** | **RMSE** | **MAPE** |
| RW1 | RW1 | RW1 | No | 2.49 | 4.14 | 0.57 | 2.30 | 1.04 | 2.73 | 0.29 | 1.90 |
| RW1 | RW1 | RW1 | Yes | 2.82 | 4.60 | 0.60 | 2.25 | 1.62 | 4.61 | 0.27 | 2.02 |
| RW1 | RW1 | RW2 | No | 6.87 | 10.15 | 2.33 | 7.94 | 2.97 | 8.76 | 1.34 | 10.02 |
| RW1 | RW1 | RW2 | Yes | 7.31 | 10.92 | 2.14 | 7.36 | 3.79 | 11.48 | 1.37 | 10.12 |
| RW1 | RW2 | RW1 | No | 6.04 | 8.89 | 2.99 | 10.95 | 3.37 | 9.99 | 2.39 | 18.30 |
| RW1 | RW2 | RW1 | Yes | 5.45 | 8.05 | 2.74 | 9.90 | 3.48 | 10.48 | 1.96 | 14.68 |
| RW1 | RW2 | RW2 | No | 6.23 | 9.08 | 3.18 | 11.57 | 3.30 | 9.78 | 2.28 | 17.48 |
| RW1 | RW2 | RW2 | Yes | 5.45 | 8.07 | 2.77 | 9.88 | 3.37 | 10.15 | 1.86 | 13.98 |
| RW2 | RW1 | RW1 | No | **2.45** | **4.08** | **0.57** | **2.28** | **1.03** | **2.68** | **0.29** | **1.89** |
| RW2 | RW1 | RW1 | Yes | 2.79 | 4.56 | 0.61 | 2.23 | 1.60 | 4.57 | 0.27 | 2.01 |
| RW2 | RW1 | RW2 | No | 6.68 | 9.84 | 2.28 | 7.76 | 2.90 | 8.54 | 1.31 | 9.79 |
| RW2 | RW1 | RW2 | Yes | 7.17 | 10.68 | 2.10 | 7.22 | 3.73 | 11.31 | 1.34 | 9.90 |
| RW2 | RW2 | RW1 | No | 6.04 | 8.89 | 2.99 | 10.95 | 3.37 | 9.99 | 2.39 | 18.31 |
| RW2 | RW2 | RW1 | Yes | 5.45 | 8.05 | 2.74 | 9.90 | 3.48 | 10.48 | 1.96 | 14.68 |
| RW2 | RW2 | RW2 | No | 6.22 | 9.07 | 3.18 | 11.55 | 3.30 | 9.77 | 2.28 | 17.46 |
| RW2 | RW2 | RW2 | Yes | 5.44 | 8.07 | 2.76 | 9.87 | 3.36 | 10.13 | 1.86 | 13.94 |

*Note:* The lowest value is shown in bold.

*Abbreviations:* OD, overdispersion; BAPC, Bayesian age-period-cohort; RW, random walk; RMSE, root mean square errors; MAPE, mean absolute percentage error.


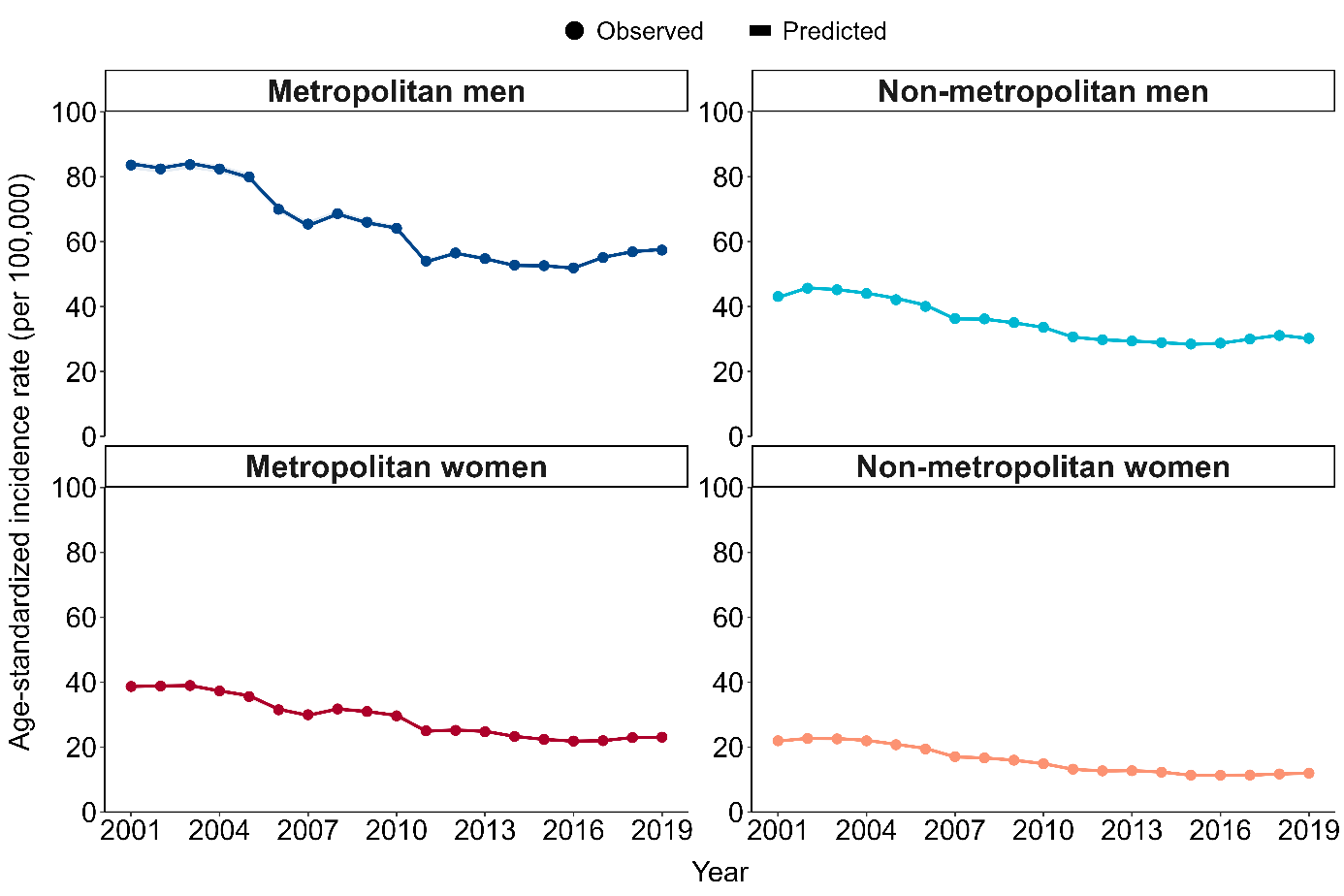


**Fig. S1.** Validation of the Bayesian age-period-cohort model based on the comparison between observed and predicted pulmonary tuberculosis incidence rates, by sex and area, in Brazil, 2001–2019.

**Table S3.** The summary estimates of all variance parameters in the BAPC models by sex and area in Brazil, 2001-2019.

| **Variance** | **Mean** | **SD** | **2.5%Q** | **Median** | **97.5%Q** |
| --- | --- | --- | --- | --- | --- |
| Metropolitan men | | | | | |
| Age | 2.66 | 0.92 | 1.24 | 2.52 | 4.83 |
| Period | 367.05 | 125.06 | 175.75 | 349.05 | 661.91 |
| Cohort | 385.28 | 106.58 | 214.15 | 372.44 | 630.29 |
| Non-metropolitan men | | | | | |
| Age | 3.21 | 1.12 | 1.50 | 3.05 | 5.84 |
| Period | 680.16 | 240.00 | 319.28 | 643.71 | 1251.47 |
| Cohort | 411.28 | 134.87 | 206.32 | 391.43 | 731.17 |
| Metropolitan women | | | | | |
| Age | 4.28 | 1.49 | 2.00 | 4.07 | 7.79 |
| Period | 361.18 | 124.57 | 174.85 | 341.95 | 659.00 |
| Cohort | 2652.15 | 915.45 | 1286.99 | 2509.53 | 4844.93 |
| Non-metropolitan women | | | | | |
| Age | 4.64 | 1.62 | 2.16 | 4.40 | 8.44 |
| Period | 440.91 | 155.60 | 207.80 | 417.01 | 812.18 |
| Cohort | 1404.77 | 564.88 | 603.76 | 1304.68 | 2788.62 |

*Note:* Larger values allow baseline effects to vary only slightly, while small values allow more heavy variation; thus, the age effect explained most of the variation in all models.

*Abbreviations:* BAPC, Bayesian age-period-cohort; SD, standard deviation; Q, quantile.

| **Table S4.** Hyperparameter estimates (95% CrI) in the BAPC models by sex and each area in Brazil, 2001–2019. | | | | | | |
| --- | --- | --- | --- | --- | --- | --- |
| **Region/State** | **Men** | | | **Women** | | |
|  | **Age** | **Period** | **Cohort** | **Age** | **Period** | **Cohort** |
| **North** |  |  |  |  |  |  |
| Acre |  |  |  |  |  |  |
| Non-metropolitan | 3.2 (1.4-6.4) | 2033.6 (70.6-9920.7) | 106.2 (40.5-229.1) | 5 (2-10.3) | 1552.4 (28.6-7486.2) | 194.5 (55.8-508.6) |
| Amapá |  |  |  |  |  |  |
| Macapá | 5.1 (2-10.3) | 47.8 (11.7-142.5) | 207 (56.3-560.9) | 2.8 (1-6.4) | 245 (17.8-1143.8) | 1618.7 (28.9-8045.3) |
| Non-metropolitan | 3.5 (1.1-8.5) | 112.5 (21.5-368.1) | 2264.5 (163.7-8729.5) | 6.6 (2.2-14.6) | 133.9 (22.2-449.3) | 2022.6 (109.2-8133) |
| Amazonas |  |  |  |  |  |  |
| Manaus | 2.9 (1.3-5.3) | 17.6 (8.4-31.8) | 567.5 (217.4-1207.3) | 5.7 (2.6-10.6) | 20.1 (9.4-37.1) | 768.4 (156.3-2573.7) |
| Non-metropolitan | 3.9 (1.7-7.3) | 207.8 (57.4-560.6) | 2890.2 (378.8-9898.4) | 5.6 (2.4-10.9) | 353.3 (70.4-1109) | 2473.9 (279.9-9029.8) |
| Pará |  |  |  |  |  |  |
| Belém | 2.2 (1-4) | 451.7 (163.2-1011.9) | 158.2 (74.7-292.5) | 4.8 (2.2-9) | 436.3 (127.1-1139) | 1599.1 (281-5320.6) |
| Santarém | 2.4 (0.9-5) | 173.2 (32.1-571.4) | 1524.1 (20.8-7793.3) | 4.9 (1.7-10.9) | 97.7 (27.6-251.6) | 2312.6 (172.6-8944.7) |
| Non-metropolitan | 3.5 (1.6-6.6) | 400.9 (148.8-877.5) | 721.6 (284.4-1519.8) | 3.8 (1.7-7.1) | 307.5 (103.3-708.6) | 839.3 (139.9-3030.1) |
| Rondônia |  |  |  |  |  |  |
| Porto Velho | 3.2 (1.3-6.5) | 17.9 (7.3-36.5) | 114.3 (38.9-264.1) | 5.3 (2-11) | 319.1 (24.4-1551.9) | 237.4 (17.8-1008.5) |
| Non-metropolitan | 3.5 (1.4-7.2) | 2034.2 (109.8-8214.3) | 217.5 (58.7-585.9) | 6.3 (2.5-12.9) | 65.9 (19.2-168.4) | 2497.6 (228.9-9312.1) |
| Roraima |  |  |  |  |  |  |
| Capital | 3.8 (1.5-8.1) | 178 (36.2-569.9) | 66.9 (24.4-148.7) | 5.6 (2-12.3) | 132 (13.9-568) | 143.6 (16.2-559.1) |
| Central | 5.2 (1.3-13.3) | 1632.6 (46.5-7142.9) | 1940.9 (88.5-8149) | 4.7 (1.1-12.4) | 2628.1 (210.9-10641.2) | 2504.6 (183.5-10,148.3) |
| Sul do Estado | 5.5 (1.6-13.2) | 2270.3 (152.9-9066.8) | 2150.1 (126.2-8715.9) | 3 (0.6-8.5) | 2127 (126.8-8509.5) | 2196.1 (144-8672.1) |
| Non-metropolitan | 7 (2.3-15.8) | 2127.8 (110.2-9098.7) | 1703.7 (45.6-7771.8) | 4 (1.1-10.3) | 11623.6 (51.2-76703.5) | 2224.1 (160.6-8699.8) |
| Tocantins |  |  |  |  |  |  |
| Gurupi | 6.2 (1.9-14.5) | 67.2 (5.2-316.9) | 182.6 (17.1-816.1) | 4.4 (1.1-11.4) | 28.5 (3.4-111.7) | 2282.9 (174.4-8698.6) |
| Palmas | 7.8 (2.9-16.6) | 100.5 (24.4-284.9) | 1336.9 (15.1-6696) | 4.6 (1.3-11.4) | 31.5 (8.5-81.4) | 2229.4 (153-8684.4) |
| Non-metropolitan | 5.6 (2-12.2) | 1867.3 (67.4-8417.4) | 223 (47.8-686.5) | 5.5 (1.9-12.4) | 1627.9 (47.2-7060.7) | 150.9 (25.8-508.5) |
| **Northeast** |  |  |  |  |  |  |
| Alagoas |  |  |  |  |  |  |
| Agreste | 4.7 (1.5-10.9) | 1973.8 (2.3-13477.2) | 745.5 (70.9-3570) | 5.1 (1.8-11.3) | 1943 (99.6-7785.4) | 177.4 (36.8-547.7) |
| Caetés | 4.8 (1.4-12) | 123.7 (17.6-465.8) | 1734.2 (54.1-7673.7) | 6.4 (2.1-14.4) | 89.4 (12.9-330.6) | 2184.8 (142.1-8589.9) |
| Maceió | 2.1 (0.9-4.1) | 2542.8 (233.7-9434.3) | 740.8 (163.1-2187.1) | 3.5 (1.4-7.1) | 1889.4 (101.1-8578) | 1654.2 (118.9-8098.8) |
| Médio Sertão | 4.3 (1.3-10.3) | 223 (24.6-917) | 1896 (86.8-7807) | 5 (1.4-12.2) | 2716.4 (221.2-10991.3) | 99.2 (9.2-423.5) |
| Palmeira dos Índios | 3.7 (0.8-10.5) | 33.4 (2.3-172.8) | 2249.6 (159.7-8706.6) | 4.3 (1.1-11.2) | 127.9 (4.1-789.6) | 2220.7 (153.8-8626.7) |
| Sertão | 5.6 (1.8-13.1) | 1990 (100.6-8154.6) | 156.8 (27.5-533.8) | 5.3 (1.5-12.9) | 113.1 (10.9-480.3) | 2225.7 (139.4-8985.8) |
| São Francisco | 5.6 (1.7-13) | 1757.7 (69.4-7244.7) | 252.7 (36.6-960.3) | 5.1 (1.6-11.8) | 1797.7 (58.6-8020.7) | 2208.9 (130.5-9203.9) |
| Vale do Paraíba | 5.3 (1.7-12.1) | 2477.9 (200.5-9659.9) | 187.1 (20.7-789.5) | 5.9 (2.1-12.8) | 1996.9 (102.3-8136.2) | 80.2 (20.2-219.1) |
| Zona da Mata | 3.5 (1.2-7.9) | 346.2 (59.5-1146.8) | 2271.3 (165.7-8734.4) | 6.1 (2.1-13.6) | 108.5 (20.7-339.7) | 2181.8 (139.6-8650.5) |
| Non-metropolitan | 3.1 (1-7.3) | 1757.7 (55.9-7816.1) | 756.5 (73.3-3511.5) | 5 (1.7-11.1) | 222.2 (25.5-944.5) | 1939.2 (2.2-13285.7) |
| Bahia |  |  |  |  |  |  |
| Feira de Santana | 4.3 (1.7-8.9) | 85.2 (22.2-236.8) | 172.1 (42.6-493) | 5 (2-10.5) | 104.9 (23.3-303.4) | 2085.5 (94.3-9554.2) |
| Salvador | 2.5 (1.2-4.7) | 240.1 (96-498.7) | 800.8 (261.2-1877.4) | 4.2 (1.9-7.9) | 373 (114.3-937.5) | 846 (185-2373.4) |
| Non-metropolitan | 3.5 (1.6-6.5) | 261.5 (109.8-523.3) | 759.5 (269.8-1719.1) | 3.9 (1.8-7.3) | 130.4 (54.5-261.2) | 992.7 (215.9-3144.5) |
| Ceará |  |  |  |  |  |  |
| Cariri | 3.9 (1.3-8.8) | 189.5 (35.3-636.1) | 2100.4 (81-12270.5) | 4.2 (1.3-9.8) | 2035.8 (108.3-8340.7) | 109.5 (30.9-279.7) |
| Fortaleza | 3.1 (1.4-5.7) | 333.2 (131.8-699.1) | 288.2 (131.6-544.7) | 5.5 (2.5-10.2) | 201.9 (72.4-452.8) | 739.8 (225.4-1840.7) |
| Sobral | 2.9 (1.2-6) | 276.8 (50.3-911.4) | 3046.5 (430-10,149.5) | 5.6 (2.2-11.4) | 184.3 (48.9-488) | 2755.7 (319.2-9499.9) |
| Non-metropolitan | 2.9 (1.3-5.5) | 1912.4 (101.9-10552.5) | 502.4 (106.6-1427.1) | 4.5 (1.9-8.9) | 143.4 (49.3-327.7) | 1906.8 (207.1-7656.4) |
| Maranhão |  |  |  |  |  |  |
| Grande São Luís | 2.2 (1-4.1) | 38.6 (17-74.8) | 290.8 (97.3-687.7) | 4.8 (2-9.4) | 36.1 (14.6-74) | 2814.7 (409-9477.7) |
| Sudoeste Maranhense | 6.1 (2.3-12.8) | 76.1 (21-202) | 496 (37.3-2482.1) | 5 (1.6-11.5) | 42.1 (14.3-95.8) | 2225.1 (148.3-8892.4) |
| Non-metropolitan | 4.5 (2.1-8.5) | 212.6 (88.3-425.8) | 2242.2 (462.1-6795.1) | 4.8 (2.1-9.1) | 146.4 (59.6-296.8) | 2298.7 (305.3-8102.4) |
| Paraíba |  |  |  |  |  |  |
| Araruna | 3.2 (0.5-10.1) | 2314.6 (175.9-8851.5) | 2169.4 (138.7-8547.6) | 3.6 (0.6-11) | 703.4 (6.2-4716.3) | 2222.7 (153-8645.5) |
| Barra de Santa Rosa | 5.9 (1.7-14.2) | 4.6 (0.9-14.8) | 2201.6 (150.2-8568.9) | 3.4 (0.7-9.7) | 1589.2 (44.2-6886.9) | 1845.2 (77.5-7676.3) |
| Cajazeiras | 6 (1.8-14.3) | 2181.2 (141-8602.5) | 182.8 (28.2-662.4) | 5.5 (1.8-12.6) | 113.9 (18.2-398.3) | 1624.8 (46.1-7103) |
| Campina Grande | 4.8 (1.8-10) | 143.8 (30.9-450) | 2100.6 (116-8683.5) | 7.3 (2.7-15.4) | 121.1 (12.1-544.2) | 2510.7 (228.5-9322.8) |
| Esperança | 4 (0.9-10.7) | 1323.8 (26.6-8961.3) | 2179.4 (136.5-8695.2) | 4.7 (1.3-11.7) | 2533.6 (208.7-9953.9) | 2415.3 (180.5-9532.4) |
| Guarabira | 5.1 (1.7-11.3) | 1335.7 (13.8-6791.8) | 490.9 (54.9-2221.3) | 5.7 (1.8-13.3) | 102.1 (10.3-433.5) | 2866.1 (262.5-11,390) |
| Itabaiana | 7.2 (2.6-15.5) | 2533.9 (217.5-9694.8) | 2590 (230.6-9925.5) | 5.3 (1.5-12.7) | 1989.6 (92.9-8352.3) | 3994.6 (32.5-26,438.1) |
| João Pessoa | 2.9 (1.3-5.5) | 251.1 (70-673.3) | 198.1 (76.3-426.3) | 3.9 (1.7-7.8) | 159 (36.7-469.6) | 2566.3 (261.9-9397.7) |
| Patos | 6.9 (2.6-14.5) | 170.6 (25-631.4) | 1520.3 (27.2-7261.7) | 5.4 (1.7-12.7) | 185.2 (27.5-670.5) | 1949.1 (96.1-7965.1) |
| Sousa | 5.3 (1.6-12.6) | 43.8 (8.7-140.9) | 2459 (217.2-9129.4) | 5.7 (1.6-14) | 96.9 (13.4-360.3) | 2199.7 (150.2-8553) |
| Vale do Mamanguape | 6 (1.8-14) | 3684.5 (17.4-25502.2) | 2529.9 (134.8-11,279.2) | 4.6 (1.2-11.9) | 2179.4 (22-12816.7) | 2771.6 (64.3-15,519.5) |
| Vale do Piancó | 5 (1.4-12.7) | 2491 (125.9-11641.1) | 1671.5 (18.8-9102.1) | 4.4 (1.2-11.3) | 2166.3 (141.8-8483.5) | 23.8 (6.6-61.2) |
| Non-metropolitan | 4.5 (1.6-9.7) | 5.1 (2.4-9.4) | 36.5 (15.7-72.3) | 6.5 (2.2-14.5) | 33 (7.8-95.4) | 2050.5 (111.4-8391.1) |
| Pernambuco |  |  |  |  |  |  |
| Recife | 3 (1.4-5.5) | 271.7 (120.8-521.5) | 335 (158.5-618) | 5.2 (2.4-9.5) | 312.9 (115.4-690.2) | 1814.1 (380.9-5735.2) |
| Non-metropolitan | 3.2 (1.4-5.9) | 1680.7 (280.5-5856.7) | 168 (69.1-346.3) | 5.5 (2.4-10.5) | 164.8 (54.6-383.9) | 2625.8 (291.5-9414.8) |
| Piauí |  |  |  |  |  |  |
| Non-metropolitan | 3.7 (1.6-7.2) | 13.8 (6.7-24.7) | 251.6 (94.2-547.5) | 4.5 (1.7-9.4) | 135.5 (39.2-342.6) | 441.6 (102.8-1262.2) |
| Rio Grande do Norte |  |  |  |  |  |  |
| Natal | 2.6 (1.2-5) | 634.8 (128.2-2116.2) | 98.5 (46.9-181.1) | 3.6 (1.5-6.9) | 751.8 (82.6-3399.1) | 507.3 (75.3-1667.2) |
| Non-metropolitan | 3.6 (1.5-7) | 388.1 (52.9-1585.4) | 156.2 (57.1-350) | 4.9 (2-10) | 106.3 (31.2-268.2) | 2778.7 (330.2-9725.3) |
| Sergipe |  |  |  |  |  |  |
| Aracaju | 3 (1.2-6.2) | 663.6 (55.2-3446.9) | 48.5 (22.4-91.5) | 5.1 (2-10.5) | 2347 (179.7-9066.1) | 167.3 (35.5-535.9) |
| Non-metropolitan | 3.5 (1.3-7.6) | 101.4 (25.2-294.2) | 147.2 (48.8-351) | 4 (1.5-8.5) | 110.8 (19.4-395.8) | 1859.4 (43.3-9510.5) |
| Southeast |  |  |  |  |  |  |
| Espírito Santo |  |  |  |  |  |  |
| Grande Vitória | 2 (0.9-3.7) | 112.8 (38.5-263.6) | 202 (70.1-468.2) | 3.2 (1.4-6.3) | 136.5 (42.8-333.5) | 2516.6 (240.7-9298.9) |
| Non-metropolitan | 2.4 (1-4.6) | 503.3 (128.7-1377.8) | 1812.5 (159.2-7457.3) | 4 (1.6-8.3) | 390 (99.5-1087.8) | 2073.9 (117.5-8491.3) |
| **Southeast** |  |  |  |  |  |  |
| Minas Gerais |  |  |  |  |  |  |
| Belo Horizonte | 3.6 (1.6-7) | 98.7 (42.4-194) | 299.9 (112-663.6) | 5.4 (2.3-10.4) | 93.3 (37.2-193.5) | 1753.7 (348.7-5563.3) |
| Vale do Aço | 5.3 (1.9-11.4) | 5.4 (2.3-10.9) | 2786.9 (335.1-9664.8) | 5.8 (2-13.1) | 6.3 (2.2-14.4) | 1871.7 (9.6-11,892) |
| Non-metropolitan | 3.7 (1.7-6.8) | 1.9 (0.9-3.4) | 237.2 (110.3-447.4) | 4.9 (2.2-9.2) | 2.1 (1-3.7) | 1629.6 (155.4-7765.2) |
| Rio de Janeiro |  |  |  |  |  |  |
| Rio de Janeiro | 2.6 (1.2-4.7) | 1236.6 (473-2654.4) | 186.7 (98.9-316.1) | 4.1 (1.9-7.5) | 1190.6 (420.5-2683.9) | 1110.8 (383.4-2542.5) |
| Non-metropolitan | 2.7 (1.3-5.1) | 279.1 (90.6-687.9) | 127.2 (58.2-239.3) | 4 (1.7-7.7) | 313.9 (83.5-850.1) | 2095.9 (333.6-7247.1) |
| São Paulo |  |  |  |  |  |  |
| Baixada Santista | 2.3 (1-4.2) | 30.3 (14.5-54.9) | 146.3 (75.3-253.9) | 3.7 (1.6-7.1) | 2765.3 (385.5-9423.8) | 536.7 (170.6-1303.1) |
| Campinas | 2.8 (1.2-5.2) | 342.7 (97.6-900.2) | 82.6 (38.7-153.9) | 4.7 (2.1-9.1) | 334.2 (53.9-1259.4) | 324.2 (84.9-875.3) |
| Ribeirão Preto | 3.4 (1.4-6.5) | 51.9 (18.1-121) | 102.2 (45.1-198.8) | 5.1 (2-10.5) | 2207.2 (125.7-9239.6) | 2925.7 (39.7-19,748.8) |
| Sorocaba | 2.4 (1-4.7) | 123 (39.2-304.2) | 156 (70.2-299.4) | 5.4 (2.2-11) | 366.1 (65.5-1218.3) | 2102.4 (101-9760.6) |
| São Paulo | 2.8 (1.3-5.2) | 24.8 (12.3-43.5) | 416.1 (217.8-710.6) | 5 (2.3-9.2) | 34.7 (16.9-61.9) | 1394.3 (583.3-2794.6) |
| Vale do Paraíba e Litoral Norte |  |  |  |  |  |  |
| Non-metropolitan | 2.4 (1.1-4.5) | 134 (63.3-244.1) | 92.7 (49.8-155.4) | 4.8 (2.2-9) | 1569.4 (232-5912.2) | 710.5 (173.4-1966.4) |
| **South** |  |  |  |  |  |  |
| Paraná |  |  |  |  |  |  |
| Apucarana | 4.7 (1.6-10.8) | 1388.8 (23.5-6434.6) | 136.3 (20.3-511) | 5.9 (2-13.2) | 59.3 (9.7-192) | 2268.7 (162.8-8776.4) |
| Campo Mourão | 5.4 (1.8-12.3) | 1542 (30.6-7246.2) | 162.5 (33.7-526.6) | 4.7 (1.3-11.8) | 5543.2 (6.2-36541.5) | 1876.4 (70.2-8121.6) |
| Cascavel | 3.9 (1.4-8.3) | 2344.1 (187.7-9050.7) | 1660.7 (43.7-7621) | 6.6 (2.4-13.9) | 1860.1 (78.4-7772.7) | 15454.7 (51.1-91283.5) |
| Curitiba | 3.4 (1.5-6.6) | 290.1 (95.9-677.8) | 2197.4 (274.4-7974.8) | 4.2 (1.8-8.3) | 136.5 (43.6-331.5) | 1773.7 (95.4-8422) |
| Londrina | 2.8 (1.1-5.6) | 5216.8 (51.7-35564.5) | 99.4 (35.4-223.2) | 3.9 (1.4-8.5) | 766.1 (12.4-4650.5) | 1079.8 (14-7122.1) |
| Maringá | 3.4 (1.3-7.4) | 1828.8 (71.9-7775.4) | 332.9 (66.2-1074.3) | 5 (1.6-11.6) | 478.5 (20.9-2680) | 2128.4 (82.7-9736.9) |
| Toledo | 2.7 (0.9-6.1) | 2257.4 (162-8726.1) | 113.5 (17.9-403.8) | 4.2 (1.3-10.3) | 1931.1 (14.7-11829.7) | 2381.5 (109.9-11,004.5) |
| Umuarama | 4.6 (1.8-9.5) | 11.1 (4.2-24.5) | 2267.7 (166-8734.8) | 4.2 (1.4-9.4) | 162.5 (9.9-837.5) | 2429.6 (154.3-10,275.8) |
| Non-metropolitan | 2.9 (1.3-5.4) | 2058.9 (136.3-10055.9) | 181.1 (70.4-386.4) | 5.5 (2.4-10.5) | 163.4 (52-394.4) | 1923.9 (285.9-6904.5) |
| Rio Grande do Sul |  |  |  |  |  |  |
| Porto Alegre | 2.3 (1-4.2) | 612.3 (243.2-1277.9) | 308 (143-578.5) | 2.7 (1.2-5.2) | 559.5 (141-1637.6) | 2517.9 (518.4-7549.9) |
| Serra Gaúcha | 4.6 (1.5-10.3) | 151.5 (21.3-639.5) | 47.7 (18.1-104) | 6.8 (2.5-14.5) | 1691.1 (49.3-7559.8) | 442.9 (49.7-1950.6) |
| Non-metropolitan | 2.8 (1.3-5.3) | 539 (158.6-1401.6) | 151.1 (74.3-271.5) | 5.6 (2.4-10.7) | 1223.1 (134.3-5027) | 571 (83.9-2394.3) |
| Santa Catarina |  |  |  |  |  |  |
| Alto Vale do Itajaí | 5.2 (1.6-12.1) | 2123.4 (127.6-8467.2) | 514.1 (27.8-3042.9) | 5.1 (1.4-12.4) | 2261.7 (161.7-8737) | 2352.6 (186.3-8924.3) |
| Carbonífera | 3.7 (1.3-8.2) | 2594.4 (272.3-9512.8) | 1692 (52.9-7296.2) | 2.4 (0.7-5.9) | 2310.8 (174.9-8997.2) | 2257.7 (159.8-8745.2) |
| Chapecó | 4.5 (1.5-10.2) | 2726.6 (284.9-9965) | 2745.5 (296.7-9865.7) | 6 (1.9-13.6) | 1861 (67.1-8265.4) | 2301.7 (159.5-9185.8) |
| Contestado | 4.1 (1.3-10) | 993.1 (78.6-5202.3) | 1651.3 (60.5-6835.2) | 5.3 (1.8-11.9) | 139.1 (18.7-512.8) | 2054.4 (115.9-8239.1) |
| Extremo Oeste | 5.9 (1.7-14.3) | 2575.2 (198.9-10444.4) | 2738.5 (237-11017.4) | 5 (1.5-11.7) | 2034.6 (109.3-8240) | 2225.6 (152.8-8673.3) |
| Florianópolis | 2.9 (1.1-6) | 79.6 (22.9-213.5) | 1813.8 (188.9-7745.9) | 4.5 (1.7-9.6) | 2193.2 (144.7-8430.2) | 2875 (358.7-9761.9) |
| Foz do Rio Itajaí | 2.5 (1-4.9) | 1084.8 (72.6-5229.3) | 281.4 (60.5-852.5) | 4.3 (1.6-8.9) | 336.3 (48.5-1208.3) | 2247.1 (134.4-9785.2) |
| Lages | 6.2 (1.9-14.3) | 2235.9 (110-10217.8) | 1194.7 (6.2-6564.2) | 4.4 (1.1-11.3) | 72.3 (5.8-337) | 2321.4 (173.5-8950.8) |
| Norte/Nordeste Catarinense | 4.6 (1.9-9.3) | 2284.2 (143.5-9620.5) | 543.2 (93.2-1836.2) | 3.9 (1.5-8.1) | 206.5 (37.9-668.7) | 2306.3 (170.6-8877.3) |
| Tubarão | 4.7 (1.4-10.9) | 2343.7 (179.7-9125.2) | 2941.1 (391.1-9824.2) | 4.1 (1.1-10.1) | 2631.5 (166.3-11674.7) | 2783.2 (191-12,203.3) |
| Vale do Itajaí | 2.2 (0.7-4.9) | 185.3 (43.3-556.4) | 158.2 (43.1-428.5) | 4 (1.3-9.4) | 2693.4 (255.1-10371.3) | 2411.6 (176-10,021.7) |
| **Central-West** |  |  |  |  |  |  |
| Distrito Federal |  |  |  |  |  |  |
| Non-metropolitan | 3.8 (1.6-7.5) | 211.3 (49.2-638.5) | 484.5 (90.9-1611.2) | 6.9 (2.9-13.6) | 230 (38.7-758.8) | 1231.6 (53.4-6813) |
| Goiás |  |  |  |  |  |  |
| Goiânia | 3.2 (1.3-6.4) | 626.3 (89.4-2523.3) | 139.6 (54.3-297.4) | 2.7 (1-5.8) | 545.3 (94.3-1857.7) | 2380.8 (201.1-8760.1) |
| Non-metropolitan | 3.3 (1.3-6.5) | 2488.3 (227.5-9632.6) | 144.6 (57.3-305.2) | 4.2 (1.7-8.7) | 2923.3 (3.6-19710.2) | 1508.9 (2.7-10,465.8) |
| Mato Grosso |  |  |  |  |  |  |
| Vale do Rio Cuiabá | 3.2 (1.4-6) | 12 (5.6-22.1) | 269 (102-574.2) | 5.1 (2.1-9.9) | 11.1 (5-21.2) | 2911.3 (381.8-9821.7) |
| Non-metropolitan | 6.4 (2.9-12) | 253.1 (76.6-642.9) | 165.7 (57.3-380.2) | 6.1 (2.7-11.9) | 155.2 (39.8-441.1) | 448.7 (91.5-1443.7) |
| Mato Grosso do Sul |  |  |  |  |  |  |
| Non-metropolitan | 4 (1.8-7.4) | 165 (52.4-410.5) | 135.1 (60.1-262.5) | 6 (2.6-11.6) | 264.4 (70.1-710.7) | 780 (131.7-2681.5) |

*Note:* Larger values allow baseline effects to vary only slightly, while small values allow more heavy variation; thus, the age effect explained most of the variation in all models.

*Abbreviations:* BAPC, Bayesian age-period-cohort; CrI, credible interval.

| **Table S5.** Epidemiological characteristics of pulmonary tuberculosis cases by area in Brazil, 2001–2020. | | | |
| --- | --- | --- | --- |
| **Variables** | **Overall**  ***N* = 1,262,968** | **Area of residence** | |
|  |  | **Metropolitan  *N* = 685,866** | **Non-metropolitan  *N* = 577,102** |
|  | ***N* (%)** | ***N* (%)** | ***N* (%)** |
| **Period (years)** |  |  |  |
| 2001-2005 | 329,400 (26.1%) | 137,453 (20.0%) | 191,947 (33.3%) |
| 2006-2010 | 308,015 (24.4%) | 140,512 (20.5%) | 167,503 (29.0%) |
| 2011-2016 | 366,632 (29.0%) | 232,076 (33.8%) | 134,556 (23.3%) |
| 2016-2020 | 258,921 (20.5%) | 175,825 (25.6%) | 83,096 (14.4%) |
| **Region of residence** |  |  |  |
| North | 133,153 (10.5%) | 65,170 (9.5%) | 67,983 (11.8%) |
| Northeast | 357,642 (28.3%) | 180,030 (26.2%) | 177,612 (30.8%) |
| Southeast | 567,676 (44.9%) | 335,818 (49.0%) | 231,858 (40.2%) |
| South | 146,249 (11.6%) | 90,754 (13.2%) | 55,495 (9.6%) |
| Central-West | 58,248 (4.6%) | 14,094 (2.1%) | 44,154 (7.7%) |
| **Age, years** |  |  |  |
| Mean (SD) | 39.4 (17.3) | 38.5 (16.8) | 40.5 (17.8) |
| Median (IQR) | 37.0 (26.0-51.0) | 36.0 (25.0-50.0) | 38.0 (27.0-52.0) |
| **Age group (years)** |  |  |  |
| 0-4 | 16,227 (1.3%) | 8421 (1.2%) | 7806 (1.4%) |
| 5-9 | 8453 (0.7%) | 4470 (0.7%) | 3983 (0.7%) |
| 10-14 | 16,110 (1.3%) | 8473 (1.2%) | 7637 (1.3%) |
| 15-19 | 79,992 (6.3%) | 46,220 (6.7%) | 33,772 (5.9%) |
| 20-24 | 155,129 (12.3%) | 91,275 (13.3%) | 63,854 (11.1%) |
| 25-29 | 151,848 (12.0%) | 86,232 (12.6%) | 65,616 (11.4%) |
| 30-34 | 135,464 (10.7%) | 75,007 (10.9%) | 60,457 (10.5%) |
| 35-39 | 124,617 (9.9%) | 67,182 (9.8%) | 57,435 (10.0%) |
| 40-44 | 118,055 (9.3%) | 62,920 (9.2%) | 55,135 (9.6%) |
| 45-49 | 109,012 (8.6%) | 58,469 (8.5%) | 50,543 (8.8%) |
| 50-54 | 95,260 (7.5%) | 51,492 (7.5%) | 43,768 (7.6%) |
| 55-59 | 77,034 (6.1%) | 41,401 (6.0%) | 35,633 (6.2%) |
| 60-64 | 58,810 (4.7%) | 30,382 (4.4%) | 28,428 (4.9%) |
| 65-69 | 43,123 (3.4%) | 21,044 (3.1%) | 22,079 (3.8%) |
| 70-74 | 31,313 (2.5%) | 14,470 (2.1%) | 16,843 (2.9%) |
| 75-79 | 21,351 (1.7%) | 9440 (1.4%) | 11,911 (2.1%) |
| 80+ | 21,170 (1.7%) | 8968 (1.3%) | 12,202 (2.1%) |
| **Sex** |  |  |  |
| Male | 850,944 (67.4%) | 460,946 (67.2%) | 389,998 (67.6%) |
| Female | 412,024 (32.6%) | 224,920 (32.8%) | 187,104 (32.4%) |
| **Self-reported race** |  |  |  |
| White | 353,036 (28.1%) | 202,582 (29.7%) | 150,454 (26.1%) |
| Brown or mixed | 486,155 (38.6%) | 271,932 (39.9%) | 214,223 (37.2%) |
| Black | 142,426 (11.3%) | 85,140 (12.5%) | 57,286 (9.9%) |
| Asian | 10,577 (0.8%) | 5663 (0.8%) | 4914 (0.9%) |
| Indigenous | 13,328 (1.1%) | 2334 (0.3%) | 10,994 (1.9%) |
| Ignored | 252,900 (20.1%) | 114,692 (16.8%) | 138,208 (24.0%) |
| **Education (years)** |  |  |  |
| Illiterate | 214,185 (17.0%) | 77,458 (11.3%) | 136,727 (23.7%) |
| 1-3 | 160,338 (12.7%) | 73,497 (10.7%) | 86,841 (15.0%) |
| 4-7 | 323,279 (25.6%) | 179,411 (26.2%) | 143,868 (24.9%) |
| 8-11 | 265,943 (21.1%) | 170,095 (24.8%) | 95,848 (16.6%) |
| ≥12 | 58,272 (4.6%) | 37,371 (5.4%) | 20,901 (3.6%) |
| Ignored | 240,951 (19.1%) | 148,034 (21.6%) | 92,917 (16.1%) |
| **HIV status** |  |  |  |
| Negative | 632,275 (50.1%) | 354,075 (51.6%) | 278,200 (48.2%) |
| Positive | 98,437 (7.8%) | 61,438 (9.0%) | 36,999 (6.4%) |
| Unknown | 532,256 (42.1%) | 270,353 (39.4%) | 261,903 (45.4%) |
| **AIDS** |  |  |  |
| No | 677,821 (53.8%) | 389,987 (57.0%) | 287,834 (50.0%) |
| Yes | 86,611 (6.9%) | 53,921 (7.9%) | 32,690 (5.7%) |
| Ignored | 495,516 (39.3%) | 240,385 (35.1%) | 255,131 (44.3%) |
| **Alcohol user** |  |  |  |
| No | 666,454 (52.9%) | 390,239 (57.0%) | 276,215 (48.0%) |
| Yes | 161,141 (12.8%) | 91,974 (13.4%) | 69,167 (12.0%) |
| Ignored | 432,954 (34.3%) | 202,405 (29.6%) | 230,549 (40.0%) |
| **Smoking** |  |  |  |
| No | 332,700 (26.4%) | 221,196 (32.3%) | 111,504 (19.4%) |
| Yes | 93,998 (7.5%) | 63,009 (9.2%) | 30,989 (5.4%) |
| Ignored | 832,901 (66.1%) | 399,866 (58.5%) | 433,035 (75.2%) |
| **Drug user** |  |  |  |
| No | 365,082 (29.0%) | 240,493 (35.2%) | 124,589 (21.6%) |
| Yes | 59,611 (4.7%) | 42,592 (6.2%) | 17,019 (3.0%) |
| Ignored | 834,886 (66.3%) | 400,965 (58.6%) | 433,921 (75.4%) |
| **Diabetes mellitus** |  |  |  |
| No | 736,190 (58.4%) | 427,870 (62.5%) | 308,320 (53.5%) |
| Yes | 73,875 (5.9%) | 45,323 (6.6%) | 28,552 (5.0%) |
| Ignored | 450,403 (35.7%) | 211,418 (30.9%) | 238,985 (41.5%) |
| **Mental disorder** |  |  |  |
| No | 781,471 (62.0%) | 456,672 (66.7%) | 324,799 (56.4%) |
| Yes | 22,868 (1.8%) | 13,239 (1.9%) | 9629 (1.7%) |
| Ignored | 455,535 (36.2%) | 214,339 (31.3%) | 241,196 (41.9%) |
| **Other comorbidities** |  |  |  |
| No | 512,252 (42.7%) | 271,144 (42.5%) | 241,108 (42.9%) |
| Yes | 100,054 (8.3%) | 56,366 (8.8%) | 43,688 (7.8%) |
| Ignored | 586,844 (48.9%) | 310,103 (48.6%) | 276,741 (49.3%) |
| **Bacteriological status** |  |  |  |
| Not confirmed | 386,114 (30.6%) | 203,370 (29.7%) | 182,744 (31.7%) |
| Confirmed | 876,854 (69.4%) | 482,496 (70.3%) | 394,358 (68.3%) |

*Note:* Self-reported race, classified as Branco (White), Preto (Black), Pardo (Brown or mixed), Amarelo (Asian) or Indígena (Indigenous).

*Abbreviations:* SD, standard deviation; IQR, interquartile range; AIDS, acquired immunodeficiency syndrome; HIV, human immunodeficiency virus.

| **Table S6.** Temporal trends and age-standardized incidence rates of pulmonary tuberculosis in Brazil, 2001–2020. | | | | | | | | | | |
| --- | --- | --- | --- | --- | --- | --- | --- | --- | --- | --- |
| **Variables** | **ASIR^a^** | | | **Segmented period** | | | | **Full period** | | |
|  | **2001** | **2020** | **2001-2020** | **Period** | **APC** | **95% *CI*** | **Trend** | **AAPC** | **95% *CI*** | **Trend** |
| **Brazil** | 39.4 | 27.1 | 32.8 | 2001-2011 | -3.51^b^ | -7.47, -2.38 | ↓ | -1.98^b^ | -2.56 , -1.39 | ↓ |
|  |  |  |  | 2011-2020 | -0.26 | -1.67, 5.53 | ↔ |  |  |  |
| **Metropolitan area** | 59.5 | 34.7 | 45.4 | 2001-2014 | -3.98^b^ | -7.22, -3.20 | ↓ | -2.67^b^ | -3.43 , -2.08 | ↓ |
|  |  |  |  | 2014-2020 | 0.22 | -2.45, 8.39 | ↔ |  |  |  |
| Sex |  |  |  | - |  |  |  |  |  |  |
| Men | 84 | 50.4 | 64.4 | 2001-2014 | -3.94^b^ | -5.33, -3.22 | ↓ | -2.45^b^ | -3.06 , -1.90 | ↓ |
|  |  |  |  | 2014-2020 | 0.84 | -1.54, 7.34 | ↔ |  |  |  |
| Women | 38.7 | 20.4 | 28.7 | 2001-2020 | -3.55^b^ | -4.24, -2.87 | ↓ | -3.55^b^ | -4.24 , -2.87 | ↓ |
| Age group (years)^c^ |  |  |  |  |  |  |  |  |  |  |
| 0-4 | 11.4 | 5.3 | 7.6 | 2001-2020 | -2.73^b^ | -3.91, -1.58 | ↓ | -2.73^b^ | -3.91 , -1.58 | ↓ |
| 5-9 | 5.8 | 2.8 | 4 | 2001-2015 | -5.32^b^ | -13.49, -3.59 | ↓ | -2.99^b^ | -4.80 , -1.63 | ↓ |
|  |  |  |  | 2015-2020 | 3.86 | -3.55, 23.57 | ↔ |  |  |  |
| 10-14 | 8 | 5.4 | 7.1 | 2001-2020 | -1.95^b^ | -2.94, -0.95 | ↓ | -1.95^b^ | -2.94 , -0.95 | ↓ |
| 15-19 | 43.6 | 29.6 | 35.7 | 2001-2020 | -1.11^b^ | -1.72, -0.51 | ↓ | -1.11^b^ | -1.72 , -0.51 | ↓ |
| 20-24 | 76.2 | 67.2 | 68.3 | 2001-2013 | -2.65^b^ | -6.47, -1.33 | ↓ | -0.26 | -1.25 , 0.62 | ↔ |
|  |  |  |  | 2013-2020 | 3.98^b^ | 0.73, 14.12 | ↑ |  |  |  |
| 25-29 | 77.8 | 60.7 | 66.5 | 2001-2014 | -3.20^b^ | -6.06, -2.24 | ↓ | -1.29^b^ | -2.20, -0.57 | ↓ |
|  |  |  |  | 2014-2020 | 2.97 | -0.51, 13.23 | ↔ |  |  |  |
| 30-34 | 81.7 | 49.7 | 60.3 | 2001-2015 | -3.80^b^ | -4.44, -3.31 | ↓ | -2.44^b^ | -2.89, -2.02 | ↓ |
|  |  |  |  | 2015-2020 | 1.49 | -0.73, 6.37 | ↔ |  |  |  |
| 35-39 | 82.5 | 42.7 | 58.9 | 2001-2015 | -4.42^b^ | -5.77, -3.83 | ↓ | -3.22^b^ | -3.84, -2.73 | ↓ |
|  |  |  |  | 2015-2020 | 0.21 | -2.62, 7.44 | ↔ |  |  |  |
| 40-44 | 95.4 | 40.3 | 63.2 | 2001-2020 | -4.59^b^ | -5.18, -4.00 | ↓ | -4.59^b^ | -5.18, -4.00 | ↓ |
| 45-49 | 93.4 | 43.1 | 66.5 | 2001-2003 | 2.86 | -4.12, 8.70 | ↔ | -3.75^b^ | -4.29, -3.19 | ↓ |
|  |  |  |  | 2003-2015 | -5.97^b^ | -8.63, -5.48 | ↓ |  |  |  |
|  |  |  |  | 2015-2020 | -0.89 | -3.27, 4.27 | ↔ |  |  |  |
| 50-54 | 88.3 | 43.4 | 66.8 | 2001-2004 | 2.32 | -1.95, 10.59 | ↔ | -3.33^b^ | -3.79, -2.74 | ↓ |
|  |  |  |  | 2004-2014 | -5.81^b^ | -8.39, -5.08 | ↓ |  |  |  |
|  |  |  |  | 2014-2020 | -1.88 | -3.60, 2.46 | ↔ |  |  |  |
| 55-59 | 87.8 | 42 | 64.8 | 2001-2020 | -3.83^b^ | -4.42, -3.24 | ↓ | -3.83^b^ | -4.42, -3.24 | ↓ |
| 60-64 | 76.6 | 41.6 | 58.8 | 2001-2013 | -4.34^b^ | -7.55, -3.54 | ↓ | -3.19^b^ | -3.83, -2.62 | ↓ |
|  |  |  |  | 2013-2020 | -1.18 | -3.03, 5.46 | ↔ |  |  |  |
| 65-69 | 73.2 | 39.1 | 54.8 | 2001-2014 | -4.51^b^ | -8.59, -3.63 | ↓ | -3.38^b^ | -4.16, -2.75 | ↓ |
|  |  |  |  | 2014-2020 | -0.88 | -3.57, 7.14 | ↔ |  |  |  |
| 70-74 | 74 | 33.6 | 51.5 | 2001-2015 | -4.37^b^ | -5.59, -1.71 | ↓ | -3.89^b^ | -4.50, -3.18 | ↓ |
|  |  |  |  | 2015-2018 | 2.04 | -6.77, 4.20 | ↔ |  |  |  |
|  |  |  |  | 2018-2020 | -9.02^b^ | -14.96, -0.94 | ↓ |  |  |  |
| 75-79 | 63.8 | 33.9 | 49 | 2001-2020 | -3.56^b^ | -4.42, -2.72 | ↓ | -3.56^b^ | -4.42, -2.72 | ↓ |
| 80+ | 52.9 | 26 | 42 | 2001-2003 | 5.88 | -3.09, 13.73 | ↔ | -3.45^b^ | -4.31, -2.68 | ↓ |
|  |  |  |  | 2003-2014 | -5.29^b^ | -9.48, -4.63 | ↓ |  |  |  |
|  |  |  |  | 2014-2018 | 2.40 | -1.90, 7.73 | ↔ |  |  |  |
|  |  |  |  | 2018-2020 | -13.03^b^ | -20.39, -5.00 | ↓ |  |  |  |
| **Non-metropolitan area** | 32 | 18.5 | 25 | 2001-2003 | 3.31 | -2.26, 7.38 | ↔ | -2.54^b^ | -2.92, -2.16 | ↓ |
|  |  |  |  | 2003-2012 | -5.38^b^ | -7.13, -4.72 | ↓ |  |  |  |
|  |  |  |  | 2012-2020 | -0.69 | -1.65, 0.57 | ↔ |  |  |  |
| Sex |  |  |  | - |  |  |  |  |  |  |
| Men | 42.8 | 27.1 | 34.8 | 2001-2003 | 2.63 | -1.90, 6.27 | ↔ | -2.36^b^ | -2.77, -1.99 | ↓ |
|  |  |  |  | 2003-2014 | -4.47^b^ | -5.42, -4.08 | ↓ |  |  |  |
|  |  |  |  | 2014-2018 | 2.99^b^ | 1.08, 6.39 | ↑ |  |  |  |
|  |  |  |  | 2018-2020 | -5.89^b^ | -9.86, -1.83 | ↓ |  |  |  |
| Women | 21.9 | 10.2 | 15.6 | 2001-2004 | -0.07 | -3.53, 6.11 | ↔ | -3.70^b^ | -4.06, -3.26 | ↓ |
|  |  |  |  | 2004-2012 | -6.87^b^ | -9.24, -6.00 | ↓ |  |  |  |
|  |  |  |  | 2012-2020 | -1.78^b^ | -2.77, -0.47 | ↓ |  |  |  |
| Age group (years)^c^ |  |  |  | - |  |  |  |  |  |  |
| 0-4 | 5.6 | 2.41 | 3.8 | 2001-2007 | -9.40^b^ | -18.83, -5.42 | ↓ | -3.29^b^ | -4.27, -2.22 | ↓ |
|  |  |  |  | 2007-2020 | -0.33 | -1.73, 2.09 | ↔ |  |  |  |
| 5-9 | 2.9 | 1 | 1.8 | 2001-2015 | -6.79^b^ | -9.02, -4.85 | ↓ | -5.26^b^ | -6.94, -3.80 | ↓ |
|  |  |  |  | 2015-2018 | 16.48 | -10.05, 24.13 | ↔ |  |  |  |
|  |  |  |  | 2018-2020 | -22.15 | -36.25, 1.56 | ↔ |  |  |  |
| 10-14 | 4.6 | 2.7 | 3.4 | 2001-2020 | -2.85^b^ | -3.90, -1.83 | ↓ | -2.85^b^ | -3.90, -1.83 | ↓ |
| 15-19 | 20 | 11.3 | 14.9 | 2001-2014 | -3.58^b^ | -4.60, -2.80 | ↓ | -2.74^b^ | -3.34, -2.22 | ↓ |
|  |  |  |  | 2014-2018 | 3.92 | -2.51, 8.68 | ↔ |  |  |  |
|  |  |  |  | 2018-2020 | -9.85^b^ | -15.81, -1.09 | ↓ |  |  |  |
| 20-24 | 34.3 | 28.5 | 30.2 | 2001-2014 | -3.35^b^ | -4.30, -2.65 | ↓ | -1.31^b^ | -1.92, -0.80 | ↓ |
|  |  |  |  | 2014-2018 | 7.54 | -3.80, 13.12 | ↔ |  |  |  |
|  |  |  |  | 2018-2020 | -4.87 | -11.46, 3.87 | ↔ |  |  |  |
| 25-29 | 39.6 | 30.6 | 33.5 | 2001-2013 | -3.45^b^ | -4.85, -2.63 | ↓ | -1.59^b^ | -2.15, -1.05 | ↓ |
|  |  |  |  | 2013-2020 | 1.67 | -0.21, 6.48 | ↔ |  |  |  |
| 30-34 | 42.8 | 25 | 32.6 | 2001-2013 | -3.65^b^ | -4.96, -3.00 | ↓ | -2.45^b^ | -2.91, -2.03 | ↓ |
|  |  |  |  | 2013-2020 | -0.35 | -1.79, 3.66 | ↔ |  |  |  |
| 35-39 | 43.1 | 23.4 | 33.5 | 2001-2003 | 2.22 | -3.66, 7.81 | ↔ | -3.23^b^ | -3.84, -2.67 | ↓ |
|  |  |  |  | 2003-2014 | -5.12^b^ | -8.62, -4.59 | ↓ |  |  |  |
|  |  |  |  | 2014-2018 | 2.47 | -0.95, 6.76 | ↔ |  |  |  |
|  |  |  |  | 2018-2020 | -8.94^b^ | -14.53, -2.98 | ↓ |  |  |  |
| 40-44 | 47.4 | 26.1 | 36.1 | 2001-2003 | 3.59 | -2.99, 8.97 | ↔ | -2.91^b^ | -3.39, -2.37 | ↓ |
|  |  |  |  | 2003-2015 | -5.51^b^ | -6.86, -5.02 | ↓ |  |  |  |
|  |  |  |  | 2015-2020 | 0.98 | -1.23, 5.32 | ↔ |  |  |  |
| 45-49 | 49.6 | 25.1 | 37.8 | 2001-2003 | 7.19 | -0.59, 14.30 | ↔ | -3.42^b^ | -3.93, -2.82 | ↓ |
|  |  |  |  | 2003-2012 | -6.53^b^ | -10.21, -5.51 | ↓ |  |  |  |
|  |  |  |  | 2012-2020 | -2.38 | -3.77, 1.32 | ↔ |  |  |  |
| 50-54 | 48.7 | 25 | 38.4 | 2001-2004 | 3.06 | -0.34, 10.12 | ↔ | -3.24^b^ | -3.57, -2.82 | ↓ |
|  |  |  |  | 2004-2012 | -6.45^b^ | -8.02, -5.57 | ↓ |  |  |  |
|  |  |  |  | 2012-2020 | -2.25^b^ | -3.12, -0.99 | ↓ |  |  |  |
| 55-59 | 49.6 | 25.4 | 38.2 | 2001-2005 | 0.57 | -1.94, 6.93 | ↔ | -3.43^b^ | -3.77, -2.83 | ↓ |
|  |  |  |  | 2005-2008 | -10.45^b^ | -12.38, -5.93 | ↓ |  |  |  |
|  |  |  |  | 2008-2020 | -2.92^b^ | -3.52, -1.70 | ↓ |  |  |  |
| 60-64 | 49.5 | 25.9 | 37.3 | 2001-2003 | 4.73 | -2.15, 9.85 | ↔ | -3.00^b^ | -3.44, -2.57 | ↓ |
|  |  |  |  | 2003-2011 | -6.53^b^ | -8.96, -5.68 | ↓ |  |  |  |
|  |  |  |  | 2011-2020 | -1.45^b^ | -2.36, -0.29 | ↓ |  |  |  |
| 65-69 | 49.6 | 23.8 | 37.5 | 2001-2004 | 2.80 | -1.35, 11.32 | ↔ | -3.31^b^ | -3.71, -2.80 | ↓ |
|  |  |  |  | 2004-2011 | -7.67^b^ | -10.37, -6.42 | ↓ |  |  |  |
|  |  |  |  | 2011-2020 | -1.80^b^ | -2.71, -0.60 | ↓ |  |  |  |
| 70-74 | 49.2 | 24.3 | 37.6 | 2001-2005 | 0.67 | -4.43, 9.50 | ↔ | -3.44^b^ | -4.06, -2.57 | ↓ |
|  |  |  |  | 2005-2008 | -10.53^b^ | -13.28, -3.37 | ↓ |  |  |  |
|  |  |  |  | 2008-2020 | -2.93 | -4.19, 0.87 | ↔ |  |  |  |
| 75-79 | 48.7 | 26.9 | 38.8 | 2001-2005 | 1.65 | -1.32, 6.47 | ↔ | -3.23^b^ | -3.60, -2.61 | ↓ |
|  |  |  |  | 2005-2009 | -11.89^b^ | -16.05, -8.64 | ↓ |  |  |  |
|  |  |  |  | 2009-2020 | -1.66^b^ | -2.39, -0.58 | ↓ |  |  |  |
| 80+ | 49 | 22.6 | 36.1 | 2001-2005 | 0.81 | -2.51, 7.31 | ↔ | -3.85^b^ | -4.32, -3.16 | ↓ |
|  |  |  |  | 2005-2009 | -12.47^b^ | -16.94, -8.72 | ↓ |  |  |  |
|  |  |  |  | 2009-2020 | -2.21^b^ | -3.11, -0.89 | ↓ |  |  |  |

*Note:* ↑ Increasing trend; ↓ Decreasing trend; ↔ Stationary trend.

*Abbreviations:* ASIR, age-standardized incidence rate; APC, annual percentage change; AAPC, average annual percent change; *CI*, confidence interval.

^a^ Incidence rate per 100,000 persons-year.

^b^ Statistically significant (*P*-value < 0.05).

^c^ The values presented correspond to age-specific incidence rates for each five-year age group.

| **Table S7.** Temporal trends and age-standardized incidence rates of pulmonary tuberculosis by sex and area described by state in Brazil, 2001–2020. | | | | | | | | | | | | |
| --- | --- | --- | --- | --- | --- | --- | --- | --- | --- | --- | --- | --- |
| **Region/State** | **Men** | | | | | | **Women** | | | | | |
|  | **ASIR^a^** | | | **AAPC** | **95% *CI*** | **Trend** | **ASIR^a^** | | | **AAPC** | **95% *CI*** | **Trend** |
|  | **2001** | **2020** | **2001-2020** |  |  |  | **2001** | **2020** | **2001-2020** |  |  |  |
| **North** | | | | | | | | | | | | |
| Acre | | | | | | | | | | | | |
| Non-metropolitan | 77.1 | 80.7 | 62.9 | -0.16 | -1.15, 0.84 | ↔ | 55.2 | 32.4 | 36.5 | -2.52 ^b^ | -3.86, -1.17 | ↓ |
| Amapá | | | | | | | | | | | | |
| Macapá | 56.2 | 42.6 | 45.6 | -2.18 ^b^ | -3.93, -0.43 | ↓ | 36.5 | 20.4 | 25.1 | -2.76 ^b^ | -4.41, -1.11 | ↓ |
| Non-metropolitan | 63.7 | 44.2 | 58.6 | -2.66 ^b^ | -4.71, -0.61 | ↓ | 47.4 | 17.5 | 34.7 | -4.77 ^b^ | -7.44, -2.10 | ↓ |
| Amazonas | | | | | | | | | | | | |
| Manaus | 111.7 | 98.2 | 100.3 | 0.02 | -1.54, 1.58 | ↔ | 86.4 | 59.8 | 59.5 | -0.86 | -2.27, 0.56 | ↔ |
| Non-metropolitan | 83.8 | 45.7 | 60.0 | -2.17 ^b^ | -3.04, -1.30 | ↓ | 59.8 | 32.7 | 46.1 | -3.16 ^b^ | -4.11, -2.22 | ↓ |
| Pará | | | | | | | | | | | | |
| Belém | 101.5 | 111.5 | 103.2 | 0.97 ^b^ | 0.28, 1.66 | ↑ | 61.5 | 53.2 | 54.0 | -0.51 | -1.24, 0.23 | ↔ |
| Santarém | 57.0 | 52.1 | 51.9 | -1.95 ^b^ | -3.38, -0.52 | ↓ | 43.8 | 36.3 | 33.4 | -2.64 ^b^ | -3.87, -1.42 | ↓ |
| Non-metropolitan | 46.0 | 38.9 | 42.6 | -1.74 ^b^ | -2.48, -1.00 | ↓ | 31.5 | 18.4 | 26.1 | -3.12 ^b^ | -4.01, -2.23 | ↓ |
| Rondônia | | | | | | | | | | | | |
| Porto Velho | 76.2 | 66.6 | 81.9 | -0.45 | -2.22, 1.32 | ↔ | 64.2 | 26.4 | 41.6 | -4.22 ^b^ | -5.62, -2.83 | ↓ |
| Non-metropolitan | 41.8 | 17.7 | 28.4 | -4.18 ^b^ | -4.97, -3.39 | ↓ | 29.0 | 6.8 | 14.6 | -6.87 ^b^ | -8.59, -5.16 | ↓ |
| Roraima | | | | | | | | | | | | |
| Capital | 57.3 | 83.8 | 52.0 | 0.86 | -0.48, 2.21 | ↔ | 31.6 | 18.6 | 26.3 | -2.45 | -4.98, 0.08 | ↔ |
| Central | 129.5 | 13.0 | 37.5 | -7.77 ^b^ | -14.89, -0.65 | ↓ | 0.0 | 7.2 | 18.4 | - | - | - |
| Sul do Estado | 47.4 | 23.9 | 30.1 | -3.49 | -8.88, 1.90 | ↔ | 7.6 | 15.6 | 13.3 | - | - | - |
| Non-metropolitan | 46.1 | 44.9 | 67.4 | -2.82 | -8.41, 2.78 | ↔ | 60.7 | 28.8 | 51.3 | -2.63 | -6.73, 1.47 | ↔ |
| Tocantins | | | | | | | | | | | | |
| Gurupi | 33.2 | 20.2 | 23.1 | -2.99 | -6.47, 0.50 | ↔ | 17.9 | 7.1 | 11.5 | -5.16 ^b^ | -10.04, -0.28 | ↓ |
| Palmas | 45.8 | 13.5 | 20.3 | -7.39 ^b^ | -9.18, -5.59 | ↓ | 28.8 | 3.1 | 11.0 | -9.13 ^b^ | -11.98, -6.29 | ↓ |
| Non-metropolitan | 27.8 | 12.6 | 18.2 | -3.60 ^b^ | -4.55, -2.65 | ↓ | 10.3 | 7.8 | 9.5 | -3.46 ^b^ | -5.67, -1.25 | ↓ |
| **Northeast** | | | | | | | | | | | | |
| Alagoas | | | | | | | | | | | | |
| Agreste | 35.4 | 19.1 | 30.0 | -2.90 ^b^ | -4.51, -1.29 | ↓ | 23.2 | 7.7 | 14.8 | -5.66 ^b^ | -7.7, -3.62 | ↓ |
| Caetés | 47.0 | 23.5 | 58.8 | -1.91 | -4.69, 0.86 | ↔ | 21.9 | 12.1 | 28.0 | -2.74 | -6, 0.52 | ↔ |
| Maceió | 69.6 | 39.1 | 62.5 | -3.59 ^b^ | -4.29, -2.88 | ↓ | 39.2 | 21.0 | 30.6 | -2.56 ^b^ | -3.14, -1.98 | ↓ |
| Médio Sertão | 37.5 | 12.9 | 26.1 | -4.67 ^b^ | -7.72, -1.63 | ↓ | 21.2 | 2.7 | 14.6 | -11.34 ^b^ | -14.79, -7.88 | ↓ |
| Palmeira dos Índios | 10.1 | 11.2 | 28.7 | - | - | - | 28.2 | 5.3 | 15.3 | - | - | - |
| Sertão | 38.5 | 12.1 | 29.4 | -6.56 ^b^ | -9.55, -3.57 | ↓ | 43.3 | 5.2 | 16.3 | -8.62 ^b^ | -13.02, -4.21 | ↓ |
| São Francisco | 57.7 | 45.5 | 39.0 | -2.42 ^b^ | -4.6, -0.25 | ↓ | 23.9 | 22.5 | 18.6 | -2.44 | -5.6, 0.73 | ↔ |
| Vale do Paraíba | 47.6 | 28.7 | 40.6 | -3.66 ^b^ | -4.99, -2.32 | ↓ | 43.2 | 8.6 | 24.9 | -8.74 ^b^ | -11.07, -6.42 | ↓ |
| Zona da Mata | 47.9 | 28.6 | 43.0 | -2.52 ^b^ | -4.47, -0.57 | ↓ | 33.6 | 11.7 | 26.0 | -4.41 ^b^ | -6.56, -2.27 | ↓ |
| Non-metropolitan | 41.7 | 20.1 | 31.2 | -2.72 ^b^ | -4.13, -1.3 | ↓ | 14.4 | 6.6 | 17.5 | -2.29 ^b^ | -4.2, -0.38 | ↓ |
| Bahia | | | | | | | | | | | | |
| Feira de Santana | 69.6 | 20.3 | 43.4 | -5.50 ^b^ | -6.88, -4.13 | ↓ | 29.4 | 9.6 | 18.0 | -5.27 ^b^ | -6.63, -3.9 | ↓ |
| Salvador | 130.6 | 48.6 | 80.7 | -5.12 ^b^ | -5.63, -4.62 | ↓ | 63.9 | 20.5 | 38.9 | -5.33 ^b^ | -5.84, -4.82 | ↓ |
| Non-metropolitan | 53.8 | 22.9 | 36.9 | -4.10 ^b^ | -4.78, -3.41 | ↓ | 31.4 | 10.4 | 18.8 | -5.47 ^b^ | -6.31, -4.62 | ↓ |
| Ceará | | | | | | | | | | | | |
| Cariri | 57.9 | 31.6 | 39.3 | -2.64 ^b^ | -3.8, -1.48 | ↓ | 33.3 | 9.8 | 19.2 | -5.96 ^b^ | -7.82, -4.11 | ↓ |
| Fortaleza | 84.0 | 61.3 | 75.9 | -1.60 ^b^ | -2.3, -0.89 | ↓ | 44.7 | 22.1 | 36.1 | -3.37 ^b^ | -4.29, -2.45 | ↓ |
| Sobral | 79.4 | 48.3 | 72.2 | -2.26 ^b^ | -3.47, -1.05 | ↓ | 66.6 | 19.8 | 38.5 | -5.67 ^b^ | -7, -4.34 | ↓ |
| Non-metropolitan | 40.1 | 18.8 | 30.1 | -3.86 ^b^ | -4.57, -3.15 | ↓ | 25.3 | 9.5 | 17.1 | -5.39 ^b^ | -6.33, -4.44 | ↓ |
| Maranhão | | | | | | | | | | | | |
| Grande São Luís | 91.9 | 68.7 | 71.1 | -1.33 ^b^ | -2.51, -0.16 | ↓ | 48.2 | 28.9 | 34.8 | -3.14 ^b^ | -4.59, -1.68 | ↓ |
| Sudoeste Maranhense | 67.5 | 31.9 | 40.8 | -4.27 ^b^ | -5.57, -2.96 | ↓ | 51.4 | 17.0 | 28.3 | -6.58 ^b^ | -8.14, -5.03 | ↓ |
| Non-metropolitan | 58.9 | 26.6 | 40.3 | -4.05 ^b^ | -4.83, -3.26 | ↓ | 39.8 | 14.6 | 25.5 | -5.99 ^b^ | -6.76, -5.22 | ↓ |
| Paraíba | | | | | | | | | | | | |
| Araruna | 19.1 | 9.9 | 14.1 | -2.47 | -8, 3.06 | ↔ | 21.2 | 9.7 | 7.3 | - | - | - |
| Barra de Santa Rosa | 44.9 | 13.0 | 14.3 | -12.01 ^b^ | -20.74, -3.28 | ↓ | 3.0 | 12.9 | 5.0 | - | - | - |
| Cajazeiras | 45.9 | 27.0 | 29.6 | -2.55 ^b^ | -3.83, -1.27 | ↓ | 23.8 | 15.5 | 15.9 | -3.88 ^b^ | -6.15, -1.61 | ↓ |
| Campina Grande | 26.2 | 32.5 | 30.6 | -0.74 | -2.2, 0.72 | ↔ | 14.9 | 9.2 | 12.7 | -2.77 ^b^ | -4.89, -0.66 | ↓ |
| Esperança | 33.3 | 13.0 | 19.9 | -1.52 | -4.9, 1.86 | ↔ | 10.3 | 7.4 | 9.8 | -1.37 | -3.49, 0.75 | ↔ |
| Guarabira | 33.5 | 35.4 | 30.4 | 0.94 | -0.92, 2.81 | ↔ | 14.3 | 9.7 | 12.3 | -2.17 | -5.15, 0.82 | ↔ |
| Itabaiana | 24.8 | 18.0 | 29.2 | -2.83 ^b^ | -5.36, -0.31 | ↓ | 13.8 | 9.4 | 13.2 | -2.44 | -5.93, 1.04 | ↔ |
| João Pessoa | 68.3 | 41.2 | 57.4 | -2.74 ^b^ | -3.82, -1.65 | ↓ | 35.9 | 14.6 | 24.1 | -4.40 ^b^ | -5.88, -2.93 | ↓ |
| Patos | 42.8 | 26.0 | 33.5 | -2.69 ^b^ | -4.72, -0.67 | ↓ | 15.0 | 12.6 | 13.2 | -2.66 | -6.01, 0.68 | ↔ |
| Sousa | 69.0 | 29.2 | 40.3 | -5.13 ^b^ | -7.86, -2.4 | ↓ | 26.5 | 13.7 | 16.3 | -4.08 ^b^ | -7.45, -0.71 | ↓ |
| Vale do Mamanguape | 30.1 | 21.5 | 30.7 | -3.56 ^b^ | -5.99, -1.13 | ↓ | 10.9 | 12.4 | 12.6 | -2.46 | -7.09, 2.16 | ↔ |
| Vale do Piancó | 22.7 | 31.4 | 22.4 | -1.15 | -5, 2.69 | ↔ | 20.5 | 12.9 | 15.2 | -5.28 ^b^ | -9.87, -0.7 | ↓ |
| Non-metropolitan | 222.7 | 21.4 | 33.2 | -13.25 ^b^ | -15.5, -10.99 | ↓ | 32.2 | 6.3 | 12.2 | -6.97 ^b^ | -8.84, -5.1 | ↓ |
| Pernambuco | | | | | | | | | | | | |
| Recife | 98.0 | 85.7 | 99.1 | -0.37 | -0.98, 0.23 | ↔ | 38.6 | 30.6 | 39.2 | -1.03 ^b^ | -1.72, -0.33 | ↓ |
| Non-metropolitan | 42.8 | 33.6 | 37.1 | -0.54 ^b^ | -1.07, -0.01 | ↓ | 23.0 | 11.6 | 16.4 | -3.29 ^b^ | -4.11, -2.47 | ↓ |
| Piauí | | | | | | | | | | | | |
| Non-metropolitan | 57.5 | 22.0 | 38.3 | -6.06 ^b^ | -7.83, -4.29 | ↓ | 29.1 | 9.8 | 17.8 | -6.05 ^b^ | -7.02, -5.09 | ↓ |
| Rio Grande do Norte | | | | | | | | | | | | |
| Natal | 67.8 | 83.9 | 61.1 | 0.63 ^b^ | 0.02, 1.23 | ↑ | 28.8 | 20.1 | 23.8 | -3.13 ^b^ | -4.01, -2.25 | ↓ |
| Non-metropolitan | 43.9 | 30.8 | 31.0 | -2.91 ^b^ | -3.8, -2.01 | ↓ | 22.0 | 9.8 | 13.8 | -4.15 ^b^ | -5.45, -2.85 | ↓ |
| Sergipe | | | | | | | | | | | | |
| Aracaju | 46.1 | 68.2 | 50.0 | 1.57 ^b^ | 0.4, 2.73 | ↑ | 20.0 | 15.7 | 17.6 | -0.88 | -2.07, 0.32 | ↔ |
| Non-metropolitan | 25.3 | 25.8 | 31.5 | 0.16 | -1.23, 1.56 | ↔ | 10.4 | 9.4 | 13.3 | -0.24 | -2.03, 1.54 | ↔ |
| **Southeast** | | | | | | | | | | | | |
| Espírito Santo | | | | | | | | | | | | |
| Grande Vitória | 65.1 | 50.9 | 53.3 | -1.18 ^b^ | -2.02, -0.35 | ↓ | 36.2 | 16.3 | 22.3 | -4.24 ^b^ | -5.12, -3.36 | ↓ |
| Non-metropolitan | 37.7 | 22.6 | 30.3 | -3.05 ^b^ | -3.61, -2.48 | ↓ | 18.1 | 9.3 | 13.6 | -3.71 ^b^ | -4.37, -3.04 | ↓ |
| Minas Gerais | | | | | | | | | | | | |
| Belo Horizonte | 24.3 | 17.1 | 27.9 | -1.77 ^b^ | -2.56, -0.99 | ↓ | 10.7 | 7.0 | 12.0 | -2.31 ^b^ | -2.9, -1.72 | ↓ |
| Vale do Aço | 5.4 | 18.9 | 26.5 | 5.94 ^b^ | 3.99, 7.89 | ↑ | 1.6 | 9.6 | 12.5 | 7.82 ^b^ | 5.89, 9.76 | ↑ |
| Non-metropolitan | 1.1 | 17.0 | 21.0 | 13.90 ^b^ | 12.77, 15.04 | ↑ | 0.5 | 6.1 | 8.7 | 12.58 ^b^ | 11.45, 13.72 | ↑ |
| Rio de Janeiro | | | | | | | | | | | | |
| Rio de Janeiro | 129.3 | 91.3 | 99.8 | -1.45 ^b^ | -1.81, -1.1 | ↓ | 57.6 | 34.2 | 44.2 | -2.66 ^b^ | -2.99, -2.32 | ↓ |
| Non-metropolitan | 53.8 | 48.3 | 48.5 | -0.65 ^b^ | -1.25, -0.06 | ↓ | 20.4 | 16.2 | 18.7 | -1.83 ^b^ | -2.61, -1.05 | ↓ |
| São Paulo | | | | | | | | | | | | |
| Baixada Santista | 121.6 | 119.6 | 118.5 | 0.08 | -1.14, 1.31 | ↔ | 53.4 | 46.7 | 45.9 | -0.85 ^b^ | -1.32, -0.39 | ↓ |
| Campinas | 44.5 | 25.3 | 32.4 | -2.14 ^b^ | -2.88, -1.39 | ↓ | 19.4 | 9.1 | 12.2 | -3.02 ^b^ | -4.1, -1.93 | ↓ |
| Ribeirão Preto | 34.6 | 28.0 | 30.2 | -0.80 | -2.05, 0.45 | ↔ | 11.2 | 8.2 | 9.7 | -0.62 | -2.02, 0.78 | ↔ |
| Sorocaba | 39.2 | 27.2 | 34.9 | -1.40 ^b^ | -2.72, -0.08 | ↓ | 16.1 | 9.4 | 12.1 | -2.52 ^b^ | -3.34, -1.71 | ↓ |
| São Paulo | 61.4 | 46.7 | 51.7 | -0.83 | -2.05, 0.38 | ↔ | 28.2 | 19.9 | 21.9 | -1.51 ^b^ | -2.51, -0.51 | ↓ |
| Vale do Paraíba e Litoral Norte | 50.4 | 37.8 | 42.5 | -0.82 ^b^ | -1.5, -0.14 | ↓ | 18.9 | 12.6 | 14.5 | -1.97 ^b^ | -2.55, -1.4 | ↓ |
| Non-metropolitan | 31.8 | 31.6 | 34.8 | 0.42 | -0.2, 1.05 | ↔ | 12.4 | 7.1 | 9.8 | -2.24 ^b^ | -2.83, -1.64 | ↓ |
| **South** | | | | | | | | | | | | |
| Paraná | | | | | | | | | | | | |
| Apucarana | 25.1 | 15.3 | 21.8 | -2.69 ^b^ | -4.49, -0.9 | ↓ | 19.5 | 2.6 | 8.9 | -7.65 ^b^ | -10.97, -4.33 | ↓ |
| Campo Mourão | 18.2 | 26.8 | 20.2 | 1.41 | -0.33, 3.15 | ↔ | 13.9 | 6.7 | 8.7 | -2.30 | -4.91, 0.3 | ↔ |
| Cascavel | 18.3 | 18.2 | 21.2 | 0.00 | -1.71, 1.7 | ↔ | 14.2 | 7.0 | 9.7 | -2.67 ^b^ | -4.9, -0.43 | ↓ |
| Curitiba | 36.8 | 19.3 | 26.8 | -3.77 ^b^ | -4.4, -3.14 | ↓ | 15.7 | 8.3 | 12.6 | -4.24 ^b^ | -5.19, -3.3 | ↓ |
| Londrina | 32.3 | 26.7 | 28.4 | -0.34 | -1.44, 0.76 | ↔ | 13.7 | 12.2 | 10.1 | -1.76 ^b^ | -3.25, -0.27 | ↓ |
| Maringá | 24.3 | 18.9 | 21.2 | -1.37 | -3.12, 0.37 | ↔ | 13.1 | 7.4 | 7.9 | -4.23 ^b^ | -7.48, -0.98 | ↓ |
| Toledo | 34.3 | 17.8 | 20.7 | -1.54 | -3.7, 0.62 | ↔ | 9.6 | 6.9 | 7.8 | -1.80 | -6.59, 2.99 | ↔ |
| Umuarama | 20.9 | 23.7 | 29.6 | 0.68 | -2.42, 3.77 | ↔ | 14.4 | 9.8 | 15.6 | -0.79 | -3.88, 2.31 | ↔ |
| Non-metropolitan | 37.6 | 25.8 | 28.9 | -2.28 ^b^ | -2.78, -1.78 | ↓ | 18.0 | 7.8 | 13.2 | -4.19 ^b^ | -5.17, -3.21 | ↓ |
| Rio Grande do Sul | | | | | | | | | | | | |
| Porto Alegre | 81.3 | 64.0 | 80.2 | -0.90 ^b^ | -1.44, -0.35 | ↓ | 33.8 | 26.4 | 34.9 | -1.16 ^b^ | -1.83, -0.5 | ↓ |
| Serra Gaúcha | 21.0 | 32.7 | 25.5 | 2.34 ^b^ | 0.93, 3.75 | ↑ | 12.1 | 11.1 | 10.8 | 0.81 | -1.16, 2.77 | ↔ |
| Non-metropolitan | 28.4 | 33.1 | 30.1 | 1.24 ^b^ | 0.67, 1.81 | ↑ | 13.7 | 12.6 | 13.1 | -0.44 | -1.06, 0.19 | ↔ |
| Santa Catarina | | | | | | | | | | | | |
| Alto Vale do Itajaí | 14.3 | 8.8 | 9.2 | -1.94 | -6.3, 2.41 | ↔ | 5.7 | 5.0 | 4.6 | 1.05 | -5.14, 7.24 | ↔ |
| Carbonífera | 25.4 | 18.2 | 30.2 | -2.33 ^b^ | -3.24, -1.42 | ↓ | 6.4 | 10.0 | 12.4 | 3.49 ^b^ | 1.53, 5.45 | ↑ |
| Chapecó | 12.4 | 10.6 | 11.1 | -0.85 | -3.81, 2.11 | ↔ | 4.4 | 5.6 | 5.3 | -0.47 | -3.45, 2.52 | ↔ |
| Contestado | 13.5 | 7.8 | 12.7 | -3.88 ^b^ | -5.32, -2.44 | ↓ | 12.3 | 5.4 | 7.9 | -4.42 ^b^ | -7.36, -1.47 | ↓ |
| Extremo Oeste | 10.4 | 4.8 | 8.0 | -3.35 ^b^ | -6.28, -0.42 | ↓ | 4.8 | 3.7 | 3.7 | -2.29 | -5.77, 1.2 | ↔ |
| Florianópolis | 36.2 | 23.9 | 37.6 | -0.80 | -2.03, 0.42 | ↔ | 13.6 | 11.9 | 16.7 | -1.40 | -2.85, 0.06 | ↔ |
| Foz do Rio Itajaí | 90.0 | 38.2 | 67.8 | -3.88 ^b^ | -4.74, -3.03 | ↓ | 44.0 | 17.1 | 34.6 | -5.02 ^b^ | -6.74, -3.3 | ↓ |
| Lages | 22.8 | 11.9 | 15.6 | -2.62 ^b^ | -4.47, -0.76 | ↓ | 16.0 | 4.6 | 8.5 | -6.24 ^b^ | -9.94, -2.54 | ↓ |
| Norte/Nordeste Catarinense | 32.3 | 22.1 | 31.3 | -1.46 ^b^ | -2.48, -0.44 | ↓ | 13.5 | 13.3 | 15.0 | -1.31 | -2.83, 0.22 | ↔ |
| Tubarão | 21.7 | 19.2 | 26.3 | -0.98 | -2.14, 0.17 | ↔ | 6.5 | 6.2 | 10.0 | -0.46 | -2.90, 1.97 | ↔ |
| Vale do Itajaí | 20.4 | 21.7 | 26.4 | -0.20 | -1.22, 0.82 | ↔ | 14.7 | 9.3 | 12.0 | -1.81 ^b^ | -2.98, -0.64 | ↓ |
| **Central-West** | | | | | | | | | | | | |
| Distrito Federal | | | | | | | | | | | | |
| Non-metropolitan | 18.7 | 10.2 | 15.6 | -3.09 ^b^ | -4.27, -1.90 | ↓ | 9.1 | 5.0 | 7.0 | -4.33 ^b^ | -5.50, -3.17 | ↓ |
| Goiás | | | | | | | | | | | | |
| Goiânia | 25.0 | 21.7 | 24.0 | 0.01 | -0.93, 0.94 | ↔ | 12.2 | 6.5 | 9.3 | -3.12 ^b^ | -4.08, -2.17 | ↓ |
| Non-metropolitan | 26.2 | 13.4 | 17.0 | -3.76 ^b^ | -4.23, -3.29 | ↓ | 12.8 | 4.7 | 7.5 | -5.75 ^b^ | -6.44, -5.06 | ↓ |
| Mato Grosso | | | | | | | | | | | | |
| Vale do Rio Cuiabá | 91.4 | 55.4 | 74.9 | -0.59 | -2.90, 1.72 | ↔ | 46.9 | 28.2 | 37.2 | -2.04 | -4.57, 0.48 | ↔ |
| Non-metropolitan | 55.5 | 26.6 | 37.4 | -3.46 ^b^ | -4.25, -2.68 | ↓ | 30.4 | 12.2 | 18.9 | -3.53 ^b^ | -4.71, -2.34 | ↓ |
| Mato Grosso do Sul | | | | | | | | | | | | |
| Non-metropolitan | 51.0 | 50.0 | 48.0 | 0.29 | -0.48, 1.07 | ↔ | 26.5 | 15.2 | 18.6 | -2.87 ^b^ | -3.65, -2.09 | ↓ |

*Note:* ↑ Increasing trend; ↓ Decreasing trend; ↔ Stationary trend. - Value unavailable due to the presence of ASIR equal to zero in one or more years.

*Abbreviations:* ASIR, age-standardized incidence rate; APC, annual percentage change; AAPC, average annual percent change; *CI*, confidence interval.

^a^ Incidence rate per 100,000 persons-year.

^b^ Statistically significant (*P*-value < 0.05).


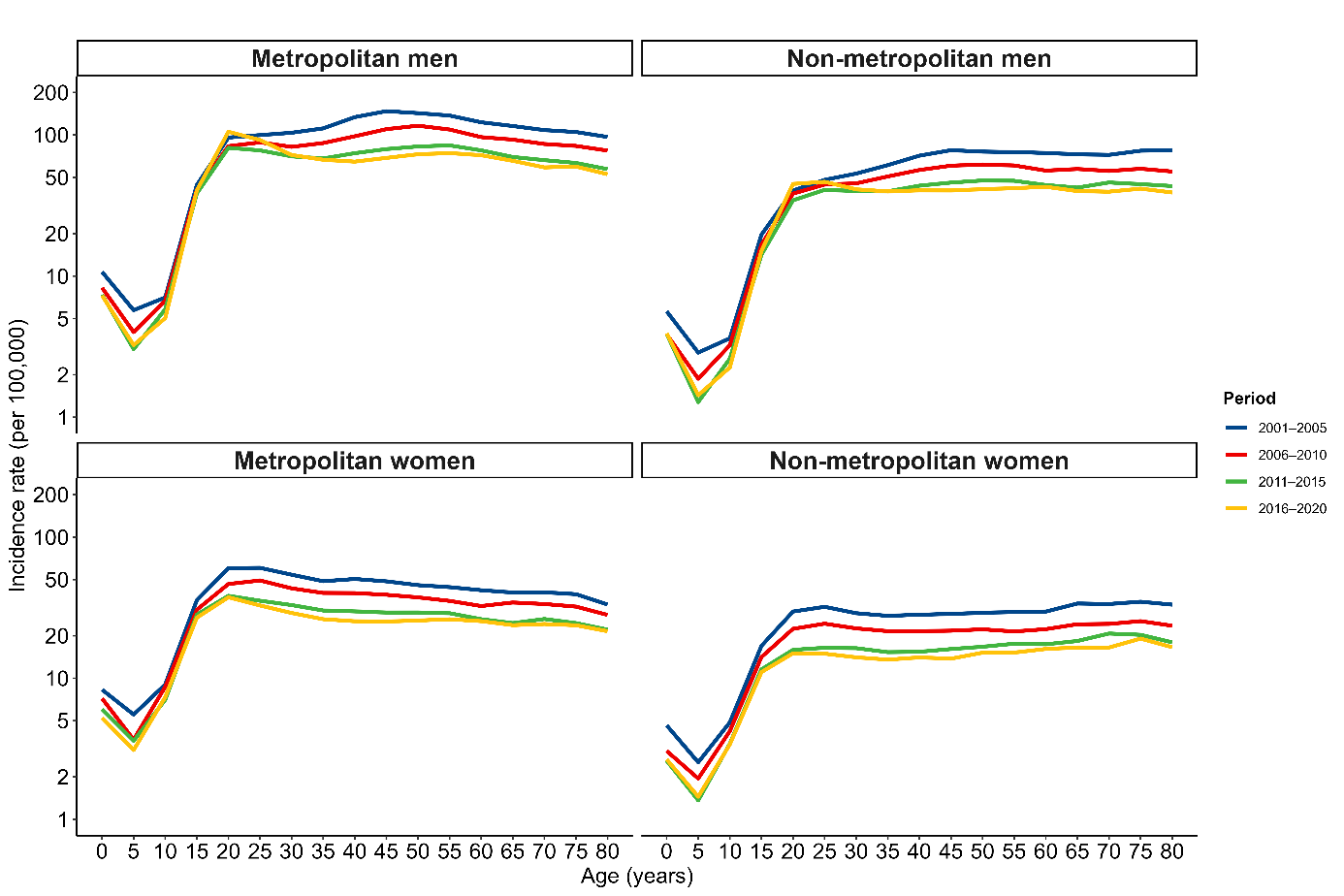


**S2 Fig.** Pulmonary tuberculosis incidence rates by age group, connected within each period, stratified by sex and area in Brazil, 2001–2020.


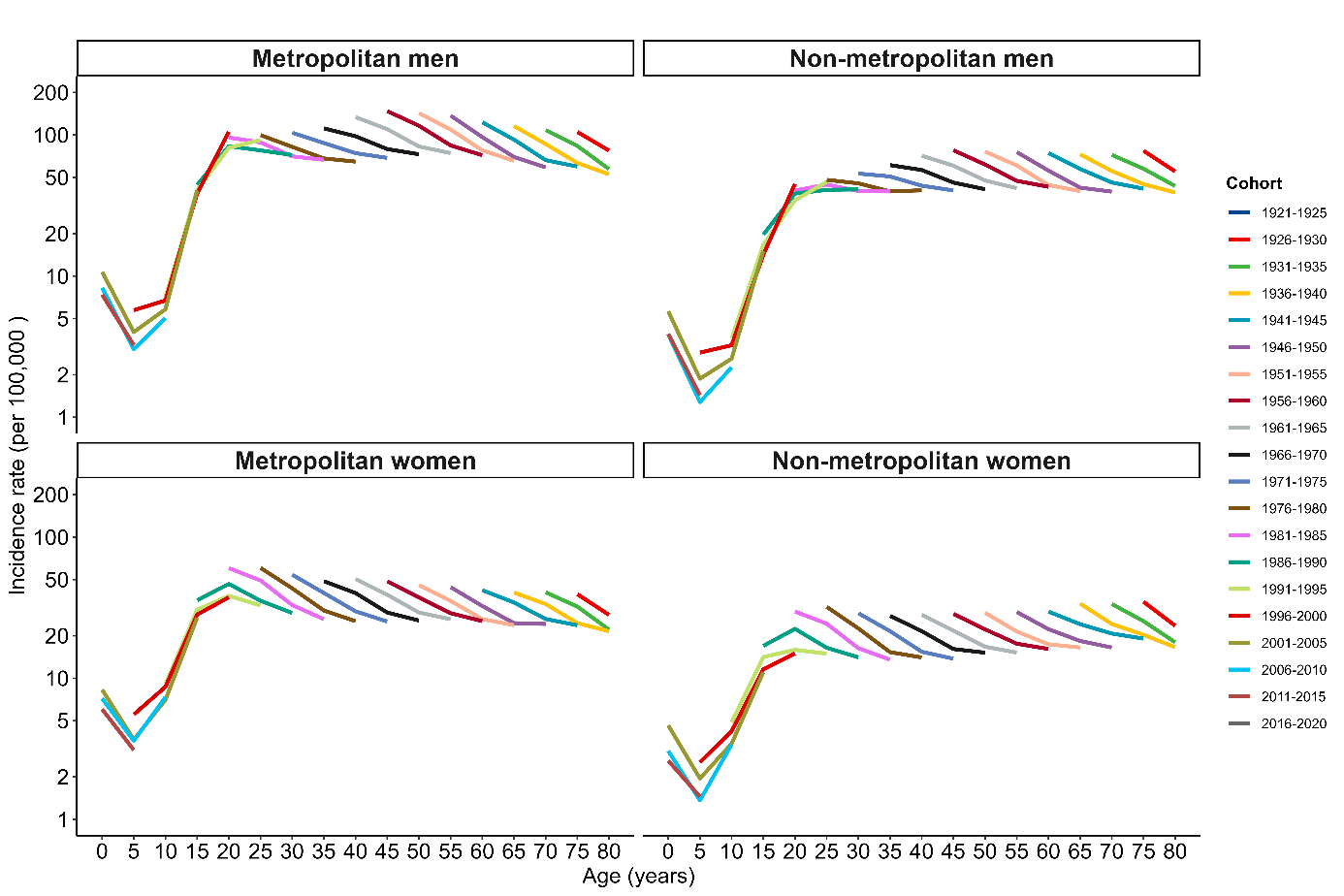


**Fig S3.** Pulmonary tuberculosis incidence rates by age group, connected within each birth cohort, stratified by sex and area in Brazil, 2001–2020.


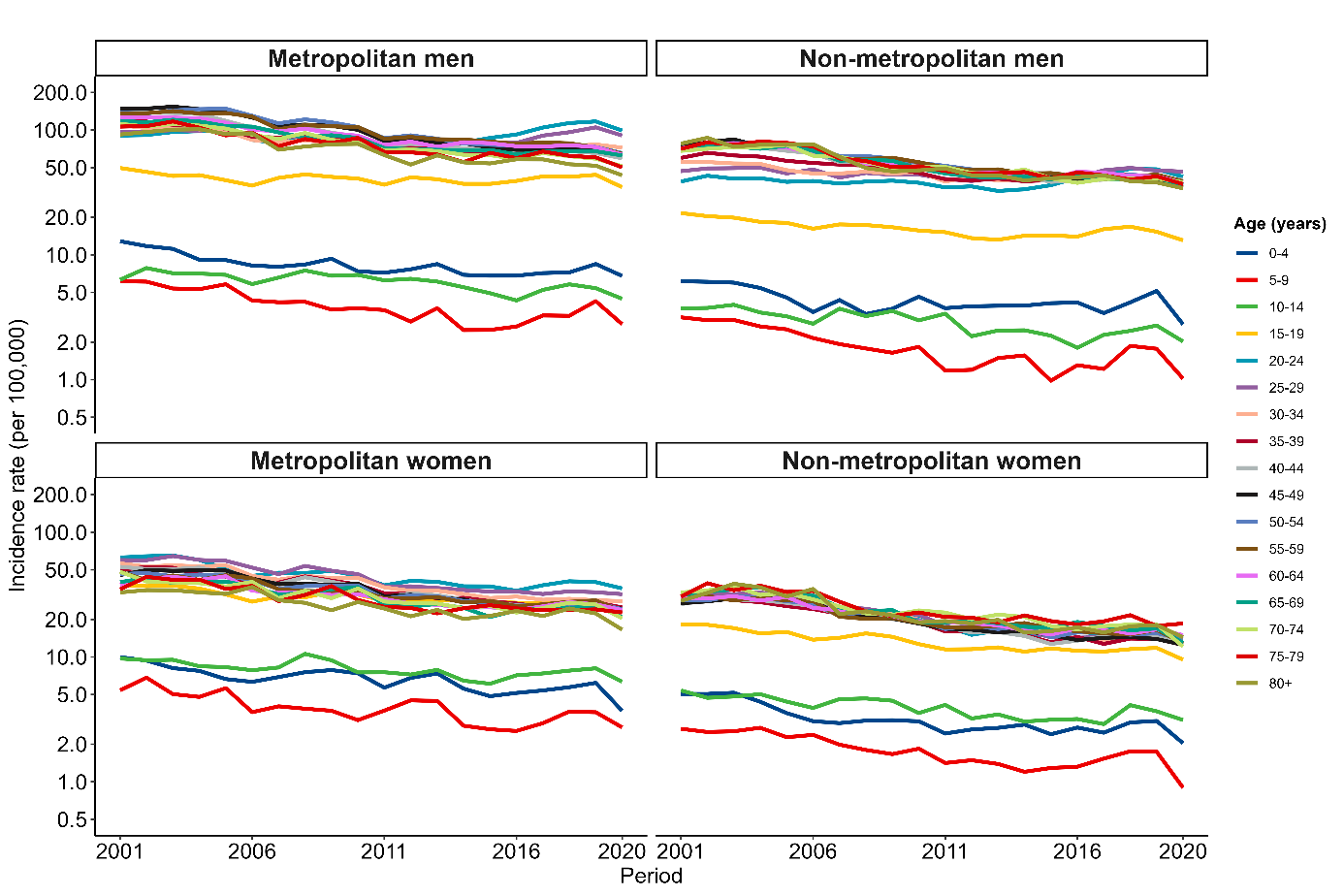


**S4 Fig.** Pulmonary tuberculosis incidence rates by period, connected within age groups, stratified by sex and area in Brazil, 2001–2020.


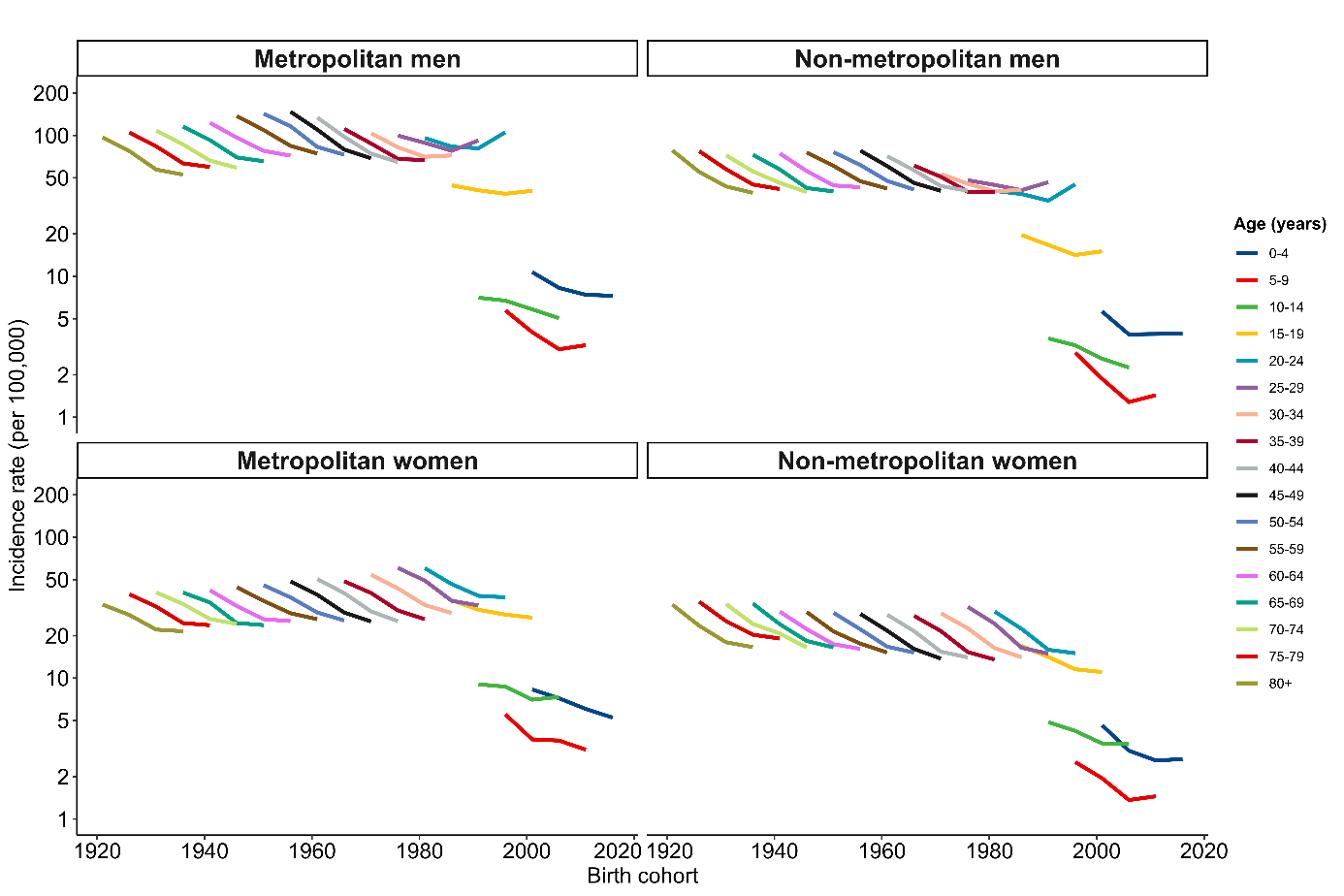


**Fig S5.** Pulmonary tuberculosis incidence rates by birth cohort, connected within age group, stratified by sex and area in Brazil, 2001–2020.

**Table S8.** Model estimates for the age-period-cohort effect on pulmonary tuberculosis incidence, by sex and area in Brazil, 2001-2020.

| **Model^a^** | **Metropolitan area** | | | | | **Non-metropolitan area** | | | | |
| --- | --- | --- | --- | --- | --- | --- | --- | --- | --- | --- |
|  | **Deviance** | **df** | **Deviance^b^** | **df^b^** | **p-value^c^** | **Deviance** | **df** | **Deviance^b^** | **df^b^** | **p-value^c^** |
| Men |  |  |  |  |  |  |  |  |  |  |
| Age | 17,183.5 | 51 |  |  |  | 14,032.9 | 51 |  |  |  |
| Age-*drift^*^* | 7749.4 | 50 | 9434.1 | 1 | <0,001 | 4342.1 | 50 | 9696.9 | 1 | <0,001 |
| Age-cohort | 1212.2 | 32 | 6537.2 | 18 | <0,001 | 979.8 | 32 | 3362.2 | 18 | <0,001 |
| Age-period-cohort | **185.3** | **30** | **1926.9** | **2** | **<0,001** | **351.2** | **30** | **628.7** | **2** | **<0,001** |
| Age-period | 6173.4 | 48 | 5988.0 | 18 | <0,001 | 3487.0 | 48 | 3135.8 | 18 | <0,001 |
| Age-*drift^**^* | 7749.4 | 50 | 1578.2 | 2 | <0,001 | 4342.1 | 50 | 885.1 | 2 | <0,001 |
| Women |  |  |  |  |  |  |  |  |  |  |
| Age | 10,008.4 | 51 |  |  |  | 12,973.0 | 51 |  |  |  |
| Age-*drift^*^* | 912.0 | 50 | 9176 | 1 | <0,001 | 681,7 | 50 | 12,291.3 | 1 | <0,001 |
| Age-cohort | 309.5 | 32 | 602.5 | 18 | <0,001 | 393.2 | 32 | 288.5 | 18 | <0,001 |
| Age-period-cohort | **78.7** | **30** | **230.7** | **2** | **<0,001** | **118.4** | **30** | **274.9** | **2** | **<0,001** |
| Age-period | 606.4 | 48 | 527.7 | 18 | <0,001 | 338.4 | 48 | 220.0 | 18 | <0,001 |
| Age-*drift^**^* | 912.0 | 50 | 305.6 | 2 | <0,001 | 681.7 | 50 | 343.3 | 2 | <0,001 |

*Note:* the chosen model is highlighted in bold.

*Abbreviations:* df, degree of freedom.

^a^ The models are ordered so that adjacent rows provide tests between the models, culminating in the age-period-cohort model

^b^ Changes in residual DF and deviance between the models in the current and previous row in the table;

^c^ p-value of the likelihood ratio test comparing the models in the current and previous row in the table

^*^ The linear trend of the logarithm of age-specific rates over time equals the sum of the slopes of the period and cohort effects;

^**^ The longitudinal age trend is the sum of the age and period slopes.

| **Table S9.** Estimates of the age–period–cohort model adjusted for pulmonary tuberculosis rates by age group, including relative risks for period and birth cohort, by sex and area, Brazil, 2001–2020. | | | | |
| --- | --- | --- | --- | --- |
|  | **Men** | | **Women** | |
|  | **Metropolitan** | **Non-metropolitan** | **Metropolitan** | **Non-metropolitan** |
| Age (years) | Incidence Rate (95% *CI*) | | | |
| 0-4 | 14.9 (14.1–15.7) | 8.2 (7.7–8.6) | 21.5 (20.2–22.8) | 14.2 (13.4–15.1) |
| 5-9 | 6.0 (5.7–6.3) | 3.0 (2.8–3.2) | 10.9 (10.3–11.6) | 6.8 (6.4–7.2) |
| 10-14 | 8.6 (8.3–9) | 4.1 (4.0–4.3) | 20.0 (19.1–20.8) | 12.4 (11.9–13) |
| 15-19 | 53.5 (52.3–54.7) | 20.9 (20.4–21.4) | 67.8 (65.9–69.9) | 34.8 (33.6–36) |
| 20-24 | 117.4 (115.3–119.6) | 47.0 (46–48) | 86.6 (84.4–88.9) | 43.9 (42.5–45.2) |
| 25-29 | 114 (112.1–115.9) | 52.8 (51.8–53.7) | 70.4 (68.8–72.1) | 36.5 (35.5–37.5) |
| 30-34 | 94.8 (93.4–96.2) | 49.1 (48.4–49.8) | 51.3 (50.3–52.4) | 26.5 (25.9–27.1) |
| 35-39 | 81.7 (80.5–82.8) | 46.3 (45.6–47) | 37.3 (36.5–38) | 19.6 (19.2–20.1) |
| 40-44 | 72.7 (71.7–73.7) | 43.1 (42.4–43.7) | 29.7 (29.1–30.3) | 15.6 (15.2–16) |
| 45-49 | 62.0 (61.1–62.9) | 37.1 (36.5–37.8) | 23.3 (22.8–23.8) | 12.4 (12.1–12.8) |
| 50-54 | 50.5 (49.6–51.4) | 30.6 (29.9–31.2) | 18.6 (18.1–19.1) | 10.3 (9.9–10.6) |
| 55-59 | 39.7 (38.9–40.5) | 25.0 (24.4–25.6) | 15.1 (14.6–15.6) | 8.3 (8.0–8.6) |
| 60-64 | 29.7 (29.0–30.4) | 19.5 (19–20.1) | 11.8 (11.4–12.2) | 6.7 (6.4–7) |
| 65-69 | 22.6 (22.0–23.3) | 15.7 (15.2–16.2) | 9.6 (9.2–10) | 5.8 (5.6–6.1) |
| 70-74 | 17.2 (16.6–17.8) | 12.9 (12.4–13.3) | 8.2 (7.8–8.6) | 4.8 (4.5–5) |
| 75-79 | 13.7 (13.2–14.3) | 10.8 (10.3–11.2) | 6.5 (6.1–6.9) | 4.0 (3.8–4.3) |
| 80+ | 10.1 (9.6–10.6) | 8.3 (7.9–8.7) | 4.9 (4.6–5.2) | 2.9 (2.7–3.1) |
| Period | Relative Risk (95% *CI*) | | | |
| 2001-2005 | 1.11 (1.10–1.12) | 1.10 (1.09–1.12) | 1.07 (1.06–1.08) | 1.10 (1.09–1.11) |
| 2006-2010 | 1.07 (1.06–1.08) | 1.07 (1.06–1.08) | 1.06 (1.05–1.07) | 1.07 (1.05–1.08) |
| 2011-2015 | Reference | Reference | Reference | Reference |
| 2016-2020 | 1.13 (1.12–1.14) | 1.13 (1.12–1.14) | 1.09 (1.08–1.10) | 1.13 (1.11–1.14) |
| Cohort | Relative Risk (95% *CI*) | | | |
| 1921-1925 | 8.65 (7.95–9.4) | 8.55 (8.04–9.11) | 6.35 (5.71–7.06) | 10.51 (9.65–11.46) |
| 1926-1930 | 7.05 (6.65–7.48) | 6.37 (6.06–6.69) | 5.53 (5.11–5.98) | 7.79 (7.26–8.36) |
| 1931-1935 | 5.69 (5.43–5.95) | 5.10 (4.89–5.32) | 4.60 (4.31–4.9) | 6.22 (5.85–6.61) |
| 1936-1940 | 4.64 (4.47–4.81) | 4.15 (4.01–4.31) | 3.92 (3.71–4.14) | 5.07 (4.80–5.36) |
| 1941-1945 | 3.82 (3.7–3.94) | 3.47 (3.36–3.58) | 3.32 (3.17–3.48) | 4.05 (3.86–4.25) |
| 1946-1950 | 3.08 (3.0–3.16) | 2.72 (2.64–2.79) | 2.66 (2.55–2.77) | 3.16 (3.02–3.3) |
| 1951-1955 | 2.58 (2.53–2.64) | 2.27 (2.21–2.32) | 2.25 (2.18–2.33) | 2.52 (2.43–2.62) |
| 1956-1960 | 2.14 (2.1–2.18) | 1.9 (1.87–1.94) | 1.94 (1.88–1.99) | 2.08 (2.02–2.15) |
| 1961-1965 | 1.66 (1.63–1.68) | 1.52 (1.49–1.54) | 1.58 (1.55–1.62) | 1.64 (1.59–1.68) |
| 1966-1970 | 1.26 (1.25–1.28) | 1.21 (1.20–1.23) | 1.25 (1.23–1.28) | 1.29 (1.26–1.32) |
| 1971-1975 | Reference | Reference | Reference | Reference |
| 1976-1980 | 0.81 (0.80–0.82) | 0.85 (0.83–0.86) | 0.80 (0.78–0.82) | 0.79 (0.78–0.81) |
| 1981-1985 | 0.73 (0.72–0.74) | 0.79 (0.77–0.8) | 0.65 (0.64–0.66) | 0.62 (0.60–0.63) |
| 1986-1990 | 0.68 (0.67–0.69) | 0.78 (0.76–0.79) | 0.51 (0.49–0.52) | 0.46 (0.45–0.47) |
| 1991-1995 | 0.71 (0.69–0.72) | 0.76 (0.74–0.77) | 0.43 (0.42–0.44) | 0.37 (0.36–0.38) |
| 1996-2000 | 0.77 (0.76–0.79) | 0.79 (0.77–0.81) | 0.41 (0.40–0.42) | 0.32 (0.31–0.33) |
| 2001-2005 | 0.67 (0.65–0.69) | 0.63 (0.61–0.65) | 0.36 (0.35–0.37) | 0.28 (0.27–0.3) |
| 2006-2010 | 0.52 (0.49–0.54) | 0.45 (0.42–0.48) | 0.33 (0.31–0.35) | 0.21 (0.20–0.23) |
| 2011-2015 | 0.49 (0.46–0.52) | 0.46 (0.43–0.5) | 0.27 (0.25–0.29) | 0.18 (0.17–0.2) |
| 2016-2020 | 0.43 (0.40–0.47) | 0.43 (0.39–0.47) | 0.22 (0.21–0.24) | 0.17 (0.15–0.19) |

*Abbreviations:* 95% *CI*, 95% confidence interval.

| **Table S10.** Changes in the number of pulmonary tuberculosis cases associated with aging, population growth, and epidemiological changes from 2001 to each year, by sex and area, using a decomposition method. | | | | | | | | | | | | | | |
| --- | --- | --- | --- | --- | --- | --- | --- | --- | --- | --- | --- | --- | --- | --- |
| **Area/Year** | **Metropolitan area** | | | | | | | **Non-metropolitan area** | | | | | | |
|  | **Aging** | | **Population** | | **Epidemiological change** | | **Overall** | **Aging** | | **Population** | | **Epidemiological change** | | **Overall** |
|  | **Change** | **%** | **Change** | **%** | **Change** | **%** | **Change** | **Change** | **%** | **Change** | **%** | **Change** | **%** | **Change** |
| Men | | | | | | | |  |  |  |  |  |  |  |
| 2001 | - | - | - | - | - | - | - | - | - | - | - | - | - | - |
| 2002 | -9 | -2.1 | 733 | 173.8 | -302 | -71.7 | 422 | -10 | -0.6 | 75 | 4.7 | 1533 | 95.9 | 1598 |
| 2003 | -60 | -5.3 | 1255 | 110.7 | -61 | -5.3 | 1134 | 11 | 0.7 | 270 | 17.9 | 1225 | 81.4 | 1506 |
| 2004 | -66 | -4.8 | 1812 | 132.9 | -384 | -28.1 | 1363 | -9 | -0.9 | 379 | 39.6 | 586 | 61.3 | 957 |
| 2005 | -100 | -4.7 | 3136 | 148.1 | -919 | -43.4 | 2117 | 16 | 5.6 | 747 | 255.0 | -471 | -160.6 | 293 |
| 2006 | -105 | -72.3 | 3453 | 2381.4 | -3203 | -2209.1 | 145 | -25 | 3.3 | 842 | -109.2 | -1587 | 205.9 | -771 |
| 2007 | 693 | 97.1 | 4429 | 620.3 | -4408 | -617.4 | 714 | 1551 | -122.6 | 1031 | -81.5 | -3847 | 304.1 | -1265 |
| 2008 | 856 | 45.5 | 4598 | 244.3 | -3572 | -189.8 | 1882 | 1799 | -144.6 | 748 | -60.1 | -3791 | 304.8 | -1244 |
| 2009 | 910 | 41.3 | 5632 | 255.4 | -4337 | -196.7 | 2205 | 2069 | -119.2 | 661 | -38.1 | -4464 | 257.3 | -1735 |
| 2010 | 1449 | 52.3 | 6195 | 223.4 | -4871 | -175.7 | 2773 | 2466 | -88.6 | 15 | -0.5 | -5265 | 189.1 | -2784 |
| 2011 | 1602 | 27.4 | 13,412 | 229.5 | -9171 | -157.0 | 5843 | 2604 | -56.2 | -127 | 2.7 | -7112 | 153.4 | -4635 |
| 2012 | 1788 | 19.9 | 15,967 | 177.2 | -8747 | -97.1 | 9008 | 2245 | -26.5 | -3809 | 44.9 | -6920 | 81.6 | -8484 |
| 2013 | 1921 | 21.4 | 16,552 | 184.9 | -9520 | -106.3 | 8953 | 2415 | -28.0 | -3903 | 45.2 | -7147 | 82.8 | -8635 |
| 2014 | 2098 | 22.5 | 17,821 | 191.3 | -10,602 | -113.8 | 9317 | 2574 | -28.6 | -4228 | 46.9 | -7362 | 81.7 | -9017 |
| 2015 | 2257 | 21.1 | 19,456 | 182.3 | -11,039 | -103.4 | 10,674 | 2611 | -27.1 | -4721 | 49.0 | -7526 | 78.1 | -9636 |
| 2016 | 2347 | 20.9 | 20,413 | 182.1 | -11,553 | -103.1 | 11,207 | 2716 | -27.9 | -5144 | 52.8 | -7306 | 75.1 | -9734 |
| 2017 | 2455 | 18.4 | 21,332 | 159.6 | -10,420 | -78.0 | 13,367 | 2821 | -31.1 | -5326 | 58.7 | -6575 | 72.4 | -9080 |
| 2018 | 2533 | 17.3 | 21,964 | 149.8 | -9834 | -67.1 | 14,663 | 2959 | -35.5 | -5369 | 64.4 | -5929 | 71.1 | -8339 |
| 2019 | 2575 | 16.7 | 22,532 | 146.0 | -9678 | -62.7 | 15,429 | 3079 | -35.2 | -5270 | 60.2 | -6568 | 75.0 | -8758 |
| 2020 | 2649 | 22.7 | 21,610 | 185.2 | -12,588 | -107.9 | 11,670 | 3147 | -31.0 | -4972 | 49.0 | -8314 | 82.0 | -10,139 |
| Women | | | | | | | |  |  |  |  |  |  |  |
| 2001 | - | - | - | - | - | - | - | - | - | - | - | - | - | - |
| 2002 | -8 | -1.9 | 383 | 93.8 | 33 | 8.1 | 408 | -3 | -0.5 | 41 | 8.4 | 457 | 92.2 | 496 |
| 2003 | -24 | -3.4 | 643 | 91.9 | 81 | 11.5 | 700 | 6 | 1.1 | 149 | 28.0 | 376 | 70.9 | 531 |
| 2004 | -23 | -4.1 | 944 | 166.2 | -353 | -62.1 | 568 | -6 | -3.0 | 199 | 95.2 | 16 | 7.8 | 209 |
| 2005 | -36 | -4.6 | 1608 | 205.9 | -791 | -101.2 | 781 | -8 | 2.1 | 366 | -95.6 | -741 | 193.4 | -383 |
| 2006 | -38 | 24.9 | 1799 | -1183.4 | -1913 | 1258.5 | -152 | -16 | 1.4 | 436 | -38.6 | -1547 | 137.2 | -1128 |
| 2007 | 168 | 171.3 | 2351 | 2399.2 | -2421 | -2470.5 | 98 | 600 | -30.4 | 559 | -28.3 | -3135 | 158.7 | -1976 |
| 2008 | 213 | 27.3 | 2451 | 314.2 | -1884 | -241.6 | 780 | 656 | -29.2 | 404 | -18.0 | -3306 | 147.2 | -2246 |
| 2009 | 205 | 20.0 | 2984 | 291.1 | -2164 | -211.1 | 1025 | 741 | -27.9 | 362 | -13.6 | -3755 | 141.6 | -2653 |
| 2010 | 383 | 35.4 | 3277 | 302.9 | -2578 | -238.3 | 1082 | 887 | -25.8 | 104 | -3.0 | -4426 | 128.8 | -3435 |
| 2011 | 409 | 15.7 | 7040 | 271.0 | -4851 | -186.7 | 2598 | 900 | -19.2 | -31 | 0.7 | -5542 | 118.6 | -4673 |
| 2012 | 412 | 11.1 | 8276 | 223.8 | -4990 | -134.9 | 3698 | 764 | -11.7 | -1944 | 29.9 | -5331 | 81.9 | -6510 |
| 2013 | 436 | 11.5 | 8587 | 226.2 | -5226 | -137.7 | 3797 | 819 | -12.7 | -1996 | 31.0 | -5262 | 81.7 | -6439 |
| 2014 | 468 | 13.0 | 9118 | 253.2 | -5984 | -166.1 | 3602 | 883 | -13.0 | -2162 | 31.9 | -5495 | 81.1 | -6774 |
| 2015 | 498 | 13.5 | 9746 | 263.9 | -6551 | -177.4 | 3693 | 873 | -11.7 | -2331 | 31.3 | -5982 | 80.4 | -7440 |
| 2016 | 503 | 13.5 | 10,141 | 271.6 | -6911 | -185.1 | 3734 | 914 | -12.1 | -2529 | 33.5 | -5940 | 78.6 | -7555 |
| 2017 | 519 | 13.2 | 10,360 | 262.5 | -6932 | -175.6 | 3947 | 944 | -12.6 | -2559 | 34.0 | -5911 | 78.5 | -7526 |
| 2018 | 522 | 11.3 | 10,677 | 230.8 | -6572 | -142.0 | 4627 | 979 | -13.4 | -2556 | 35.0 | -5725 | 78.4 | -7301 |
| 2019 | 547 | 11.3 | 10,889 | 225.7 | -6610 | -137.0 | 4825 | 1027 | -14.4 | -2564 | 36.0 | -5596 | 78.5 | -7133 |
| 2020 | 521 | 15.7 | 10,545 | 316.8 | -7738 | -232.4 | 3329 | 1023 | -12.8 | -2380 | 29.7 | -6663 | 83.1 | -8020 |

| **Table S11.** Changes in the number of pulmonary tuberculosis cases attributed to ageing, population growth, and epidemiological changes from 2001 to each year, by sex and area described by state, using a decomposition method. | | | | | | | | |
| --- | --- | --- | --- | --- | --- | --- | --- | --- |
| **Region/State** | **Men** | | | | **Women** | | | |
|  | **Aging** | **Population** | **Epidemiological change** | **Overall change** | **Aging** | **Population** | **Epidemiological change** | **Overall change** |
| **North** | | | | | | | | |
| Acre | | | | | | | | |
| Non-metropolitan | 39 | 100 | 41 | 180 | 23 | 56 | -74 | 5 |
| Amapá | | | | | | | | |
| Macapá | 23 | 42 | -16 | 49 | 11 | 27 | -35 | 3 |
| Non-metropolitan | 7 | 14 | -12 | 9 | 3 | 9 | -21 | -8 |
| Amazonas | | | | | | | | |
| Manaus | 165 | 397 | -61 | 501 | 110 | 299 | -258 | 150 |
| Non-metropolitan | 64 | 84 | -174 | -26 | 44 | 54 | -108 | -10 |
| Pará | | | | | | | | |
| Belém | 172 | 188 | 170 | 529 | 85 | 117 | -100 | 102 |
| Santarém | 17 | 23 | -6 | 33 | 12 | 18 | -14 | 16 |
| Non-metropolitan | 191 | 294 | -124 | 362 | 108 | 175 | -265 | 18 |
| Rondônia | | | | | | | | |
| Porto Velho | 24 | 54 | -3 | 75 | 17 | 33 | -66 | -16 |
| Non-metropolitan | 36 | 17 | -124 | -71 | 21 | 14 | -112 | -78 |
| Roraima | | | | | | | | |
| Capital | 15 | 74 | 66 | 154 | 7 | 25 | -25 | 7 |
| Central | 3 | 3 | -11 | -5 | 0 | 0 | 1 | 1 |
| Sul do Estado | 2 | 4 | -5 | 0 | 0 | 1 | 2 | 3 |
| Non-metropolitan | 0 | 7 | -2 | 5 | 1 | 5 | -4 | 2 |
| Tocantins | | | | | | | | |
| Gurupi | 6 | 4 | -10 | -1 | 1 | 2 | -10 | -7 |
| Palmas | 12 | 22 | -51 | -18 | 6 | 14 | -49 | -29 |
| Non-metropolitan | 19 | 12 | -54 | -23 | 5 | 6 | -8 | 3 |
| **Northeast** | | | | | | | | |
| Alagoas | | | | | | | | |
| Agreste | 16 | 7 | -37 | -13 | 8 | 5 | -41 | -28 |
| Caetés | 3 | 3 | -10 | -5 | 1 | 2 | -5 | -2 |
| Maceió | 46 | 57 | -153 | -50 | 13 | 40 | -113 | -60 |
| Médio Sertão | 4 | 1 | -15 | -11 | 2 | 0 | -12 | -10 |
| Palmeira dos Índios | 1 | 0 | 0 | 1 | 1 | 0 | -4 | -3 |
| Sertão | 3 | 1 | -17 | -13 | 3 | 1 | -26 | -21 |
| São Francisco | 7 | 1 | -6 | 2 | 3 | 1 | 0 | 3 |
| Vale do Paraíba | 8 | -2 | -13 | -7 | 3 | -1 | -27 | -25 |
| Zona da Mata | 10 | 0 | -29 | -20 | 6 | 1 | -31 | -23 |
| Non-metropolitan | 12 | 1 | -30 | -17 | 3 | 1 | -12 | -8 |
| Bahia | | | | | | | | |
| Feira de Santana | 45 | 32 | -182 | -105 | 13 | 20 | -91 | -58 |
| Salvador | 290 | 165 | -1,318 | -863 | 73 | 126 | -826 | -628 |
| Non-metropolitan | 428 | 136 | -1,386 | -822 | 182 | 100 | -996 | -715 |
| Ceará | | | | | | | | |
| Cariri | 24 | 26 | -62 | -12 | 10 | 15 | -68 | -43 |
| Fortaleza | 206 | 274 | -342 | 139 | 74 | 149 | -441 | -218 |
| Sobral | 31 | 25 | -60 | -4 | 20 | 18 | -102 | -64 |
| Non-metropolitan | 120 | 62 | -355 | -173 | 66 | 42 | -277 | -169 |
| Maranhão | | | | | | | | |
| Grande São Luís | 102 | 143 | -123 | 122 | 42 | 83 | -141 | -17 |
| Sudoeste Maranhense | 18 | 11 | -47 | -18 | 12 | 9 | -56 | -36 |
| Non-metropolitan | 197 | 132 | -638 | -309 | 113 | 96 | -510 | -301 |
| Paraíba | | | | | | | | |
| Araruna | 1 | 0 | -3 | -2 | 1 | 0 | -3 | -2 |
| Barra de Santa Rosa | 2 | 1 | -12 | -9 | 0 | 0 | 3 | 4 |
| Cajazeiras | 6 | 4 | -16 | -6 | 3 | 2 | -6 | -1 |
| Campina Grande | 15 | 13 | 18 | 47 | 3 | 6 | -19 | -10 |
| Esperança | 4 | 1 | -11 | -6 | 1 | 1 | -2 | 0 |
| Guarabira | 4 | 1 | 3 | 9 | 1 | 0 | -5 | -3 |
| Itabaiana | 2 | 1 | -3 | -1 | 1 | 1 | -3 | -1 |
| João Pessoa | 46 | 80 | -141 | -14 | 16 | 45 | -132 | -71 |
| Patos | 6 | 4 | -18 | -8 | 3 | 2 | -2 | 3 |
| Sousa | 5 | 3 | -22 | -15 | 2 | 1 | -8 | -5 |
| Vale do Mamanguape | 2 | 2 | -5 | -1 | 1 | 1 | 1 | 3 |
| Vale do Piancó | 5 | 0 | 5 | 11 | 1 | 0 | -4 | -3 |
| Non-metropolitan | 44 | 32 | -554 | -478 | 6 | 6 | -73 | -62 |
| Pernambuco | | | | | | | | |
| Recife | 249 | 223 | -168 | 304 | 67 | 112 | -167 | 12 |
| Non-metropolitan | 165 | 146 | -184 | 127 | 70 | 75 | -280 | -135 |
| Piauí | | | | | | | | |
| Non-metropolitan | 146 | 80 | -501 | -276 | 62 | 45 | -302 | -194 |
| Rio Grande do Norte | | | | | | | | |
| Natal | 79 | 129 | 143 | 351 | 22 | 47 | -64 | 5 |
| Non-metropolitan | 62 | 38 | -102 | -2 | 25 | 19 | -104 | -60 |
| Sergipe | | | | | | | | |
| Aracaju | 29 | 66 | 102 | 197 | 8 | 25 | -18 | 15 |
| Non-metropolitan | 30 | 19 | 8 | 56 | 10 | -5 | -18 | 15 |
| **Southeast** | | | | | | | | |
| Espírito Santo | | | | | | | | |
| Grande Vitória | 48 | 136 | -117 | 67 | 7 | 69 | -182 | -106 |
| Non-metropolitan | 46 | 50 | -140 | -44 | 9 | 26 | -82 | -46 |
| Minas Gerais |  |  |  |  |  |  |  |  |
| Belo Horizonte | 79 | 94 | -190 | -17 | 17 | 48 | -109 | -44 |
| Vale do Aço | 7 | 4 | 49 | 60 | 3 | 3 | 29 | 35 |
| Non-metropolitan | 106 | 82 | 1,081 | 1,270 | 25 | 34 | 392 | 452 |
| Rio de Janeiro |  |  |  |  |  |  |  |  |
| Rio de Janeiro | 468 | 860 | -2,335 | -1,007 | 35 | 433 | -1,572 | -1,104 |
| Non-metropolitan | 94 | 240 | -112 | 222 | 12 | 102 | -81 | 32 |
| São Paulo |  |  |  |  |  |  |  |  |
| Baixada Santista | 47 | 183 | -21 | 210 | -10 | 94 | -64 | 20 |
| Campinas | 53 | 139 | -273 | -82 | 10 | 64 | -153 | -79 |
| Ribeirão Preto | 31 | 52 | -50 | 33 | 4 | 18 | -23 | -1 |
| Sorocaba | 37 | 86 | -112 | 11 | 7 | 36 | -66 | -23 |
| São Paulo | 499 | 841 | -1,362 | -21 | 25 | 457 | -894 | -412 |
| Vale do Paraíba e Litoral Norte | 46 | 105 | -141 | 10 | 17 | 45 | -77 | -16 |
| Non-metropolitan | 206 | 303 | -26 | 483 | 44 | 107 | -327 | -176 |
| **South** | | | | | | | | |
| Paraná | | | | | | | | |
| Apucarana | 3 | 2 | -14 | -10 | 1 | 1 | -26 | -23 |
| Campo Mourão | 4 | 0 | 15 | 19 | 5 | 1 | -13 | -8 |
| Cascavel | 7 | 9 | 0 | 16 | 4 | 6 | -19 | -9 |
| Curitiba | 58 | 95 | -280 | -127 | 13 | 48 | -130 | -69 |
| Londrina | 13 | 27 | -29 | 11 | 3 | 14 | -9 | 8 |
| Maringá | 9 | 23 | -21 | 11 | 2 | 12 | -22 | -8 |
| Toledo | 9 | 11 | -30 | -9 | 1 | 4 | -5 | 0 |
| Umuarama | 5 | 4 | 3 | 12 | 4 | 3 | -8 | -2 |
| Non-metropolitan | 104 | 66 | -217 | -47 | 30 | 33 | -198 | -135 |
| Rio Grande do Sul | | | | | | | | |
| Porto Alegre | 147 | 113 | -346 | -86 | 21 | 64 | -160 | -75 |
| Serra Gaúcha | 13 | 23 | 47 | 84 | 3 | 11 | -4 | 10 |
| Non-metropolitan | 138 | 52 | 121 | 311 | 19 | 29 | -39 | 9 |
| Santa Catarina |  |  |  |  |  |  |  |  |
| Alto Vale do Itajaí | 3 | 4 | -8 | -1 | 0 | 2 | -1 | 1 |
| Carbonífera | 7 | 16 | -22 | 1 | -1 | 7 | 10 | 16 |
| Chapecó | 7 | 8 | -3 | 11 | 1 | 4 | 3 | 8 |
| Contestado | 3 | 4 | -14 | -7 | 2 | 3 | -18 | -13 |
| Extremo Oeste | 5 | 1 | -10 | -3 | 2 | 1 | -1 | 2 |
| Florianópolis | 23 | 74 | -74 | 23 | 3 | 34 | -11 | 26 |
| Foz do Rio Itajaí | 20 | 118 | -160 | -23 | 4 | 59 | -83 | -21 |
| Lages | 8 | 2 | -18 | -9 | 5 | 1 | -21 | -15 |
| Norte/Nordeste Catarinense | 24 | 57 | -67 | 14 | 4 | 29 | 0 | 33 |
| Tubarão | 6 | 8 | -5 | 9 | 1 | 3 | 0 | 3 |
| Vale do Itajaí | 8 | 30 | 0 | 38 | 3 | 17 | -20 | 0 |
| **Central-West** | | | | | | | | |
| Distrito Federal |  |  |  |  |  |  |  |  |
| Non-metropolitan | 32 | 54 | -96 | -10 | 12 | 31 | -57 | -14 |
| Goiás |  |  |  |  |  |  |  |  |
| Goiânia | 38 | 90 | -24 | 104 | 13 | 40 | -64 | -12 |
| Non-metropolitan | 78 | 104 | -233 | -52 | 31 | 50 | -153 | -72 |
| Mato Grosso |  |  |  |  |  |  |  |  |
| Vale do Rio Cuiabá | 72 | 77 | -149 | 0 | 30 | 45 | -96 | -21 |
| Non-metropolitan | 84 | 146 | -274 | -44 | 36 | 79 | -168 | -53 |
| Mato Grosso do Sul |  |  |  |  |  |  |  |  |
| Non-metropolitan | 76 | 164 | 1 | 241 | 30 | 74 | -139 | -35 |


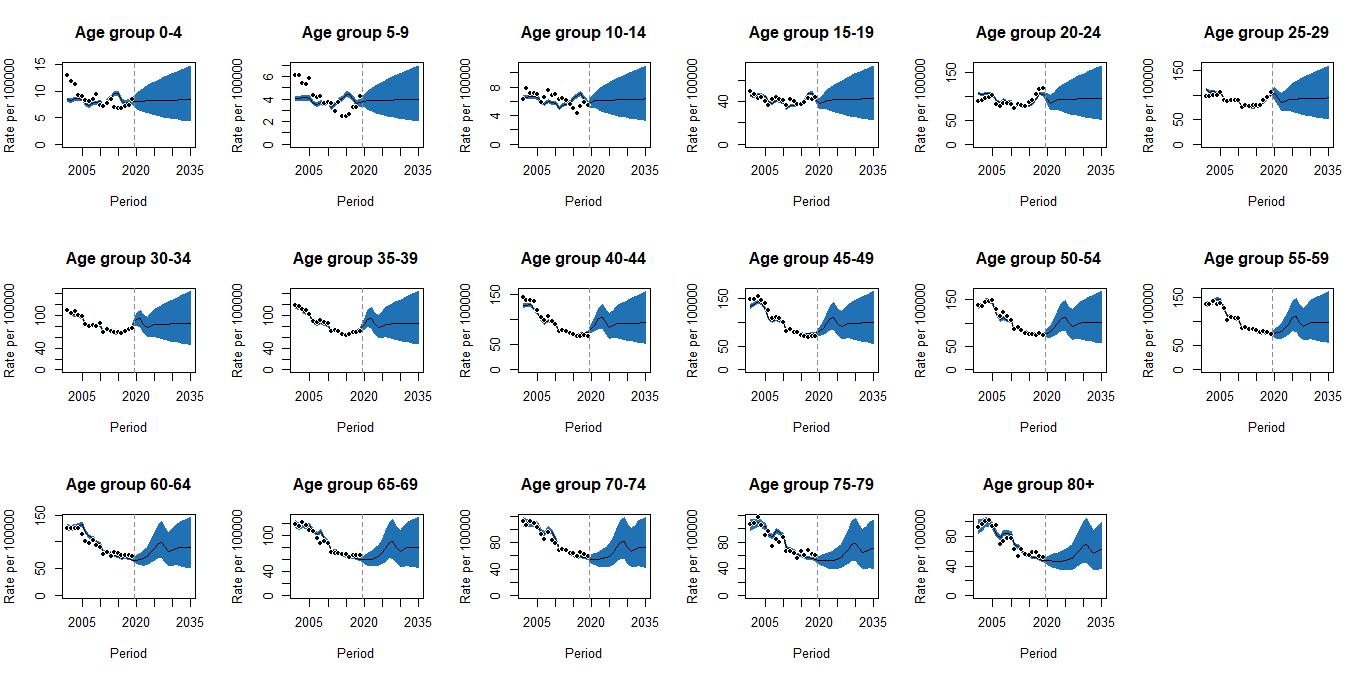


**Fig S6.** Age-specific predicted incidence rates of pulmonary tuberculosis among metropolitan men in Brazil, 2001–2035.


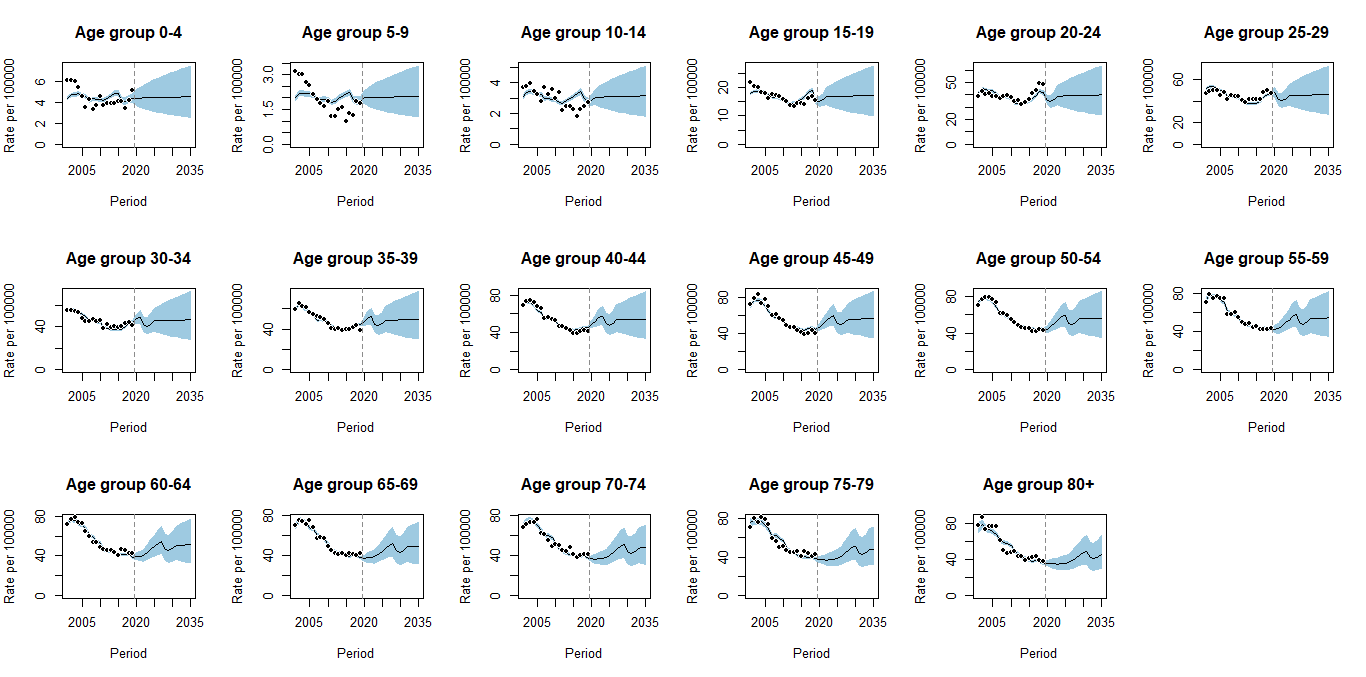


**Fig S7.** Age-specific predicted incidence rates of pulmonary tuberculosis among non-metropolitan men in Brazil, 2001–2035.


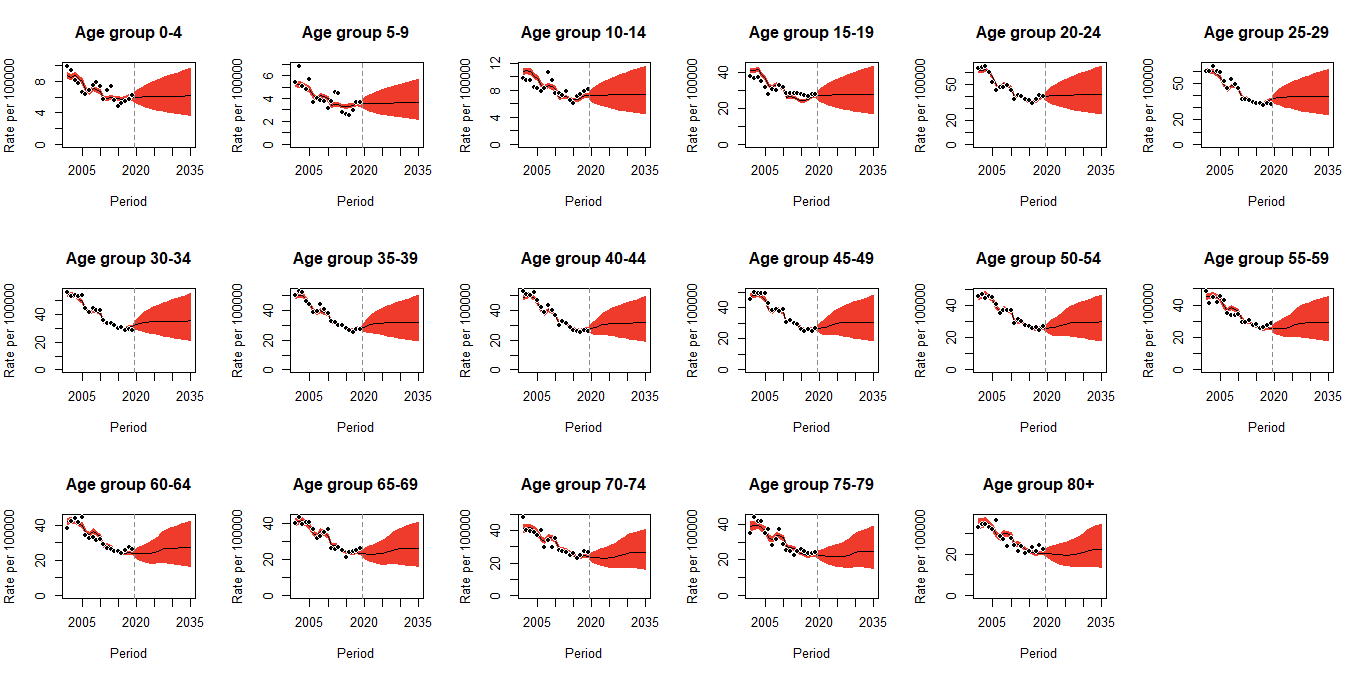


**Fig S8.** Age-specific predicted incidence rates of pulmonary tuberculosis among metropolitan women in Brazil, 2001–2035.


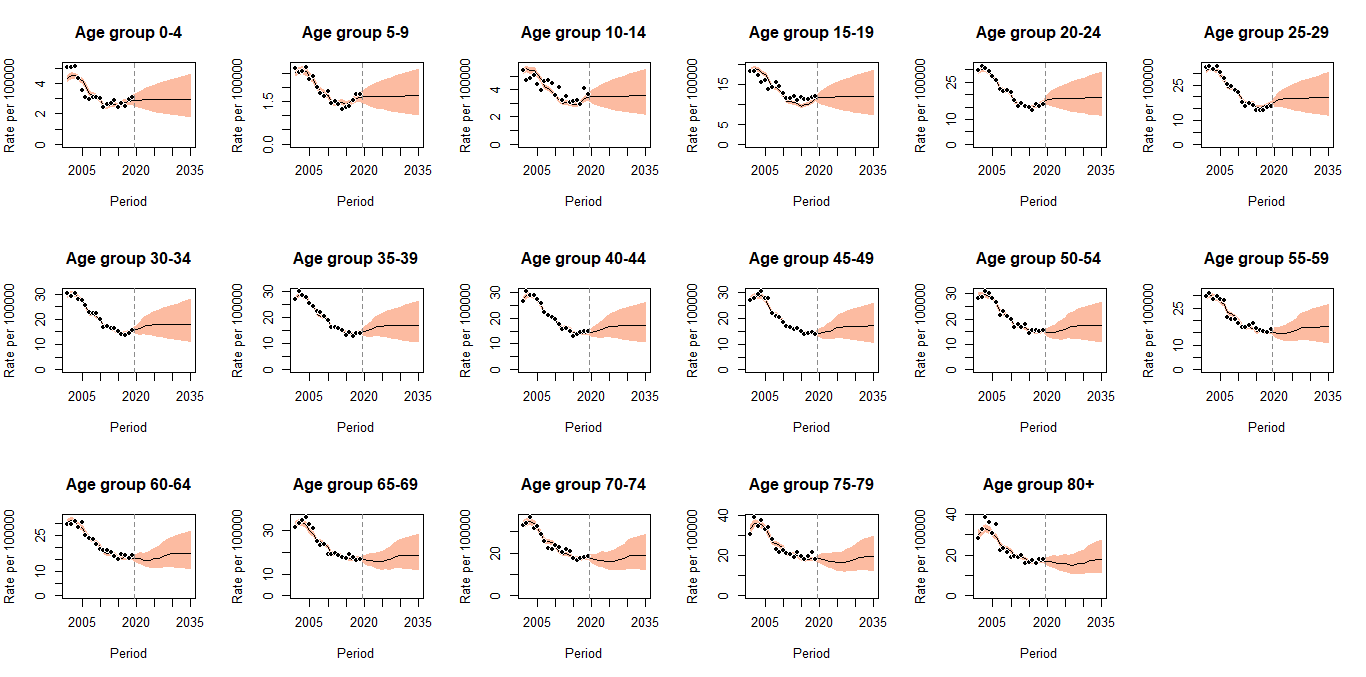


**Fig S10.** Age-specific predicted incidence rates of pulmonary tuberculosis among non-metropolitan women in Brazil, 2001–2035.

| **Table S12**. Pulmonary tuberculosis cases and age-standardized incidence rates (per 100,000) observed in 2015 and projected for 2020, 2025, 2030, and 2035, by sex and area described by state, using Bayesian age-period-cohort models. | | | | | | | | | | | |
| --- | --- | --- | --- | --- | --- | --- | --- | --- | --- | --- | --- |
|  |  | **Men** | | | | | **Women** | | | | |
|  |  | **2015** | **2020** | **2025** | **2030** | **2035** | **2015** | **2020** | **2025** | **2030** | **2035** |
| **North** | | | | | | | | | | | |
| Acre | | | | | | | | | | | |
| Non-metropolitan | Cases^*^ | 184 | 343 (289–397) | 389 (269–510) | 415 (203–627) | 434 (127–741) | 83 | 115 (88–143) | 118 (73–162) | 125 (59–191) | 132 (44–220) |
|  | ASIR^*^ | 49.4 | 79.1 (69.9–88.3) | 87 (61.5–112.6) | 87 (43.3–130.7) | 85.7 (25.6–145.8) | 21.6 | 26.8 (22.6–31) | 26.4 (17.7–35.1) | 26.1 (13.1–39.1) | 25.9 (9.2–42.6) |
| Amapá | | | | | | | | | | | |
| Macapá | Cases^*^ | 62 | 134 (76–192) | 158 (13–304) | 168 (-43–379) | 174 (-92–440) | 59 | 64 (41–88) | 67 (26–107) | 71 (16–127) | 75 (5–144) |
|  | ASIR^*^ | 25.7 | 44.1 (26.6–61.5) | 50 (4.6–95.4) | 50.4 (-12.3–113.1) | 49.8 (-25.9–125.5) | 20 | 20.8 (15–26.6) | 20.8 (9.2–32.4) | 20.8 (5.3–36.3) | 20.8 (2.1–39.4) |
| Non-metropolitan | Cases^*^ | 27 | 45 (25–65) | 45 (13–77) | 47 (5–89) | 48 (-3–99) | 11 | 20 (8–31) | 20 (5–35) | 21 (2–40) | 22 (-1–45) |
|  | ASIR^*^ | 36.1 | 50.4 (33.4–67.4) | 50.1 (17.8–82.3) | 50 (7.5–92.4) | 49.8 (-0.8–100.5) | 17.2 | 23.9 (15.2–32.7) | 23.5 (8.8–38.2) | 23.3 (4.3–42.4) | 23.3 (0.8–45.8) |
| Amazonas | | | | | | | | | | | |
| Manaus | Cases^*^ | 1215 | 1500 (755–2246) | 1714 (-360–3788) | 1836 (-1179–4851) | 1891 (-1863–5645) | 726 | 884 (466–1303) | 975 (-139–2090) | 1034 (-568–2637) | 1062 (-928–3053) |
|  | ASIR^*^ | 108.8 | 114 (57.6–170.3) | 126.3 (-26.4–279.1) | 132.6 (-85–350.2) | 133.9 (-131.8–399.6) | 61 | 65.6 (34.9–96.3) | 69.1 (-9.7–147.9) | 71.6 (-39.3–182.5) | 71.9 (-62.8–206.6) |
| Non-metropolitan | Cases^*^ | 311 | 405 (319–490) | 426 (246–606) | 453 (197–709) | 477 (153–802) | 195 | 272 (216–328) | 288 (183–393) | 307 (160–454) | 324 (138–510) |
|  | ASIR^*^ | 55.9 | 53.5 (43.4–63.6) | 54.1 (31.7–76.4) | 54.3 (24–84.5) | 54.2 (17.7–90.7) | 35.9 | 38.9 (32.4–45.4) | 39.4 (25.8–53) | 39.7 (21.2–58.1) | 39.6 (17.3–62) |
| Pará | | | | | | | | | | | |
| Belém | Cases | 1089 | 1635 (1429–1842) | 1969 (1362–2575) | 2116 (1103–3128) | 2168 (779–3557) | 649 | 672 (569–776) | 704 (487–920) | 746 (436–1056) | 775 (384–1165) |
|  | ASIR | 93.8 | 141.9 (125.3–158.5) | 170.1 (118.2–222) | 178 (93.1–262.9) | 177.8 (64.1–291.4) | 49.1 | 53.7 (46.5–60.8) | 55.9 (39.2–72.5) | 56.9 (33.6–80.2) | 57 (28.5–85.4) |
| Santarém | Cases^*^ | 57 | 81 (53–109) | 89 (39–139) | 97 (27–167) | 103 (15–191) | 33 | 50 (29–70) | 53 (18–88) | 56 (8–104) | 60 (0–120) |
|  | ASIR^*^ | 38 | 42.8 (31.3–54.4) | 44.2 (21.2–67.2) | 45.1 (13.9–76.2) | 45.1 (7.4–82.8) | 19.7 | 26 (17.9–34.1) | 25.8 (10.1–41.6) | 25.9 (4.9–46.8) | 25.8 (0.8–50.8) |
| Non-metropolitan | Cases^*^ | 844 | 1182 (1022–1343) | 1363 (965–1761) | 1529 (908–2150) | 1633 (810–2456) | 432 | 615 (510–720) | 692 (444–940) | 786 (402–1170) | 850 (340–1359) |
|  | ASIR^*^ | 34.3 | 40.6 (35.6–45.6) | 44.8 (31.9–57.6) | 46.9 (28–65.8) | 47.1 (23.5–70.7) | 18.6 | 22.2 (18.9–25.6) | 23.8 (15.5–32.1) | 25.1 (13–37.2) | 25.4 (10.3–40.5) |
| Rondônia | | | | | | | | | | | |
| Porto Velho | Cases^*^ | 182 | 260 (121–399) | 278 (-75–632) | 273 (-202–747) | 271 (-305–847) | 90 | 76 (50–101) | 75 (32–117) | 76 (17–134) | 79 (5–153) |
|  | ASIR^*^ | 45.8 | 99.5 (47.7–151.2) | 103.4 (-27.4–234.2) | 99.7 (-73.5–272.9) | 97.7 (-109.5–304.9) | 26.1 | 29 (21.9–36.1) | 27.2 (13–41.4) | 26.2 (6.9–45.5) | 26 (2.2–49.9) |
| Non-metropolitan | Cases^*^ | 129 | 131 (103–160) | 126 (84–167) | 124 (64–184) | 128 (49–207) | 50 | 48 (26–70) | 49 (11–87) | 52 (0–104) | 54 (-10–119) |
|  | ASIR^*^ | 22.3 | 21.6 (18.8–24.4) | 20.4 (14.7–26.1) | 19.2 (10.5–27.8) | 18.9 (7.7–30.1) | 8.9 | 8.1 (5.2–11.1) | 8.1 (2.2–14) | 8.1 (0.3–15.9) | 8.1 (-1.3–17.4) |
| Roraima | | | | | | | | | | | |
| Capital | Cases^*^ | 67 | 169 (120–217) | 251 (107–396) | 305 (47–562) | 332 (-37–700) | 32 | 62 (35–89) | 78 (19–137) | 92 (-2–186) | 102 (-28–232) |
|  | ASIR^*^ | 40.9 | 68.6 (51.9–85.3) | 89.1 (38.9–139.2) | 96.5 (15.7–177.3) | 95 (-10–200) | 17.2 | 25.4 (16.6–34.3) | 27.8 (7.7–47.9) | 29.1 (-0.1–58.4) | 29.2 (-7.4–65.8) |
| Central | Cases^*^ | 3 | 5 (0–10) | 6 (0–11) | 6 (0–12) | 7 (0–13) | 1 | 2 (-1–5) | 2 (-1–6) | 3 (-1–6) | 3 (-1–6) |
|  | ASIR^*^ | 22.8 | 32 (21–43) | 32.2 (17.9–46.5) | 32.3 (15.1–49.5) | 32.3 (12.6–52.1) | 8.6 | 15.6 (9.3–21.9) | 15.5 (8.5–22.6) | 15.5 (7.8–23.2) | 15.5 (7.2–23.9) |
| Sul do Estado | Cases^*^ | 4 | 9 (3–15) | 10 (3–16) | 10 (3–18) | 11 (3–19) | 4 | 3 (-1–6) | 3 (-1–7) | 3 (-1–7) | 4 (-1–8) |
|  | ASIR^*^ | 17.6 | 27.4 (20.1–34.8) | 27.4 (18.2–36.5) | 27.4 (16.6–38.3) | 27.4 (15–39.8) | 20.4 | 9.6 (5.8–13.4) | 9.6 (5.3–13.8) | 9.6 (4.8–14.3) | 9.5 (4.3–14.7) |
| Non-metropolitan | Cases^*^ | 10 | 18 (9–28) | 22 (10–34) | 25 (11–40) | 29 (11–47) | 5 | 14 (5–23) | 17 (4–29) | 20 (3–36) | 23 (2–44) |
|  | ASIR^*^ | 56.3 | 60.3 (45.2–75.4) | 60.3 (39.7–81) | 60.3 (34.3–86.2) | 60.2 (29.7–90.8) | 33.2 | 48.3 (29.8–66.8) | 48.7 (20.8–76.6) | 49.1 (14–84.1) | 49.1 (8.1–90.1) |
| Tocantins | | | | | | | | | | | |
| Gurupi | Cases^*^ | 7 | 19 (6–32) | 20 (-3–43) | 21 (-10–52) | 21 (-17–60) | 7 | 6 (-1–12) | 6 (-4–16) | 6 (-6–19) | 7 (-9–22) |
|  | ASIR^*^ | 7.2 | 19 (9.1–28.9) | 19.6 (-1.2–40.5) | 19.5 (-8.7–47.6) | 19.4 (-14.8–53.5) | 7.8 | 6.1 (1.5–10.7) | 6.1 (-2.6–14.7) | 6.1 (-5.2–17.3) | 6 (-7.3–19.3) |
| Palmas | Cases^*^ | 32 | 35 (19–52) | 39 (11–66) | 41 (5–78) | 44 (-2–89) | 12 | 14 (3–25) | 15 (-3–34) | 17 (-8–42) | 18 (-13–50) |
|  | ASIR^*^ | 14.2 | 14.4 (9.6–19.2) | 14.7 (5.5–23.9) | 14.9 (2.5–27.3) | 14.9 (0–29.8) | 5.7 | 5.8 (2.5–9) | 5.7 (-0.4–11.9) | 5.7 (-2.4–13.8) | 5.7 (-3.9–15.3) |
| Non-metropolitan | Cases^*^ | 53 | 63 (44–82) | 70 (41–99) | 77 (36–119) | 80 (27–134) | 25 | 31 (18–44) | 34 (15–53) | 37 (11–64) | 40 (6–73) |
|  | ASIR^*^ | 13.1 | 14.5 (12–17.1) | 16 (10.7–21.4) | 17 (8.7–25.4) | 17.2 (6.4–27.9) | 6.1 | 7.5 (5.7–9.2) | 8 (4.5–11.6) | 8.3 (3.1–13.6) | 8.4 (1.7–15.1) |
| **Northeast** | | | | | | | | | | | |
| Alagoas | | | | | | | | | | | |
| Agreste | Cases^*^ | 45 | 64 (45–84) | 66 (38–94) | 70 (32–108) | 72 (25–119) | 26 | 31 (18–43) | 31 (15–48) | 32 (11–54) | 33 (7–60) |
|  | ASIR^*^ | 17.8 | 22.9 (18.8–27) | 23.2 (15.2–31.1) | 23.5 (12.1–35) | 23.6 (9.3–37.8) | 8.8 | 10.3 (8.2–12.5) | 10.2 (6.2–14.2) | 10.2 (4.4–16) | 10.2 (2.9–17.4) |
| Caetés | Cases^*^ | 17 | 24 (11–37) | 24 (5–43) | 25 (0–50) | 26 (-4–57) | 8 | 11 (3–18) | 11 (0–21) | 11 (-2–25) | 12 (-4–28) |
|  | ASIR^*^ | 35.1 | 45.7 (28.8–62.7) | 46.7 (14.8–78.7) | 47.1 (4.7–89.4) | 47 (-3.6–97.6) | 14.7 | 19 (10.2–27.8) | 18.9 (3.7–34.2) | 18.9 (-0.9–38.6) | 18.9 (-4.5–42.2) |
| Maceió | Cases^*^ | 302 | 315 (269–362) | 344 (264–425) | 376 (255–497) | 395 (238–552) | 169 | 166 (134–198) | 173 (125–221) | 184 (118–250) | 195 (111–278) |
|  | ASIR^*^ | 53.7 | 53.2 (48–58.4) | 56.1 (44.4–67.8) | 58 (40.3–75.6) | 58.2 (35.8–80.6) | 25.4 | 25.1 (22.1–28.2) | 25.3 (19.4–31.2) | 25.6 (17.2–34) | 25.6 (15.2–36) |
| Médio Sertão | Cases^*^ | 6 | 14 (5–23) | 14 (3–25) | 14 (1–28) | 15 (0–30) | 7 | 7 (1–14) | 9 (0–17) | 9 (-1–20) | 9 (-3–22) |
|  | ASIR^*^ | 8.8 | 19.3 (12.4–26.2) | 19.2 (8–30.5) | 19.3 (4.8–33.8) | 19.2 (2.1–36.4) | 10.2 | 10.1 (6.2–14) | 11.4 (3.7–19.1) | 11.8 (0.6–23.1) | 11.8 (-2.3–25.8) |
| Palmeira dos Índios | Cases^*^ | 0 | 2 (-2–6) | 2 (-3–7) | 2 (-4–8) | 2 (-5–9) | 0 | 2 (-1–4) | 2 (-2–5) | 2 (-2–5) | 2 (-2–6) |
|  | ASIR^*^ | 0 | 13.7 (-0.9–28.4) | 13.7 (-11.1–38.5) | 13.7 (-18.1–45.5) | 13.6 (-23.7–50.8) | 0 | 8.8 (0.9–16.7) | 8.8 (-3.2–20.8) | 8.8 (-6.1–23.7) | 8.7 (-8.5–26) |
| Sertão | Cases^*^ | 11 | 15 (6–23) | 14 (4–23) | 14 (2–26) | 14 (0–28) | 8 | 9 (2–16) | 9 (0–18) | 9 (-2–20) | 9 (-4–22) |
|  | ASIR^*^ | 16.5 | 19.6 (14.1–25.1) | 18 (9.2–26.9) | 17.7 (5.5–29.9) | 17.6 (2.7–32.6) | 10.2 | 11.3 (5.9–16.7) | 11.3 (2.1–20.6) | 11.3 (-0.6–23.2) | 11.3 (-2.8–25.3) |
| São Francisco | Cases^*^ | 10 | 18 (8–27) | 18 (7–29) | 18 (5–32) | 18 (3–34) | 7 | 11 (4–18) | 11 (3–18) | 11 (3–19) | 11 (3–19) |
|  | ASIR^*^ | 17.2 | 28.8 (21.3–36.2) | 29.4 (17–41.9) | 29.5 (12.2–46.9) | 29.5 (8.1–50.9) | 10.6 | 16.9 (12.8–21.1) | 17 (11.2–22.7) | 16.9 (9.8–24.1) | 16.9 (8.6–25.2) |
| Vale do Paraíba | Cases^*^ | 23 | 22 (12–33) | 20 (8–32) | 19 (4–34) | 19 (1–36) | 12 | 11 (4–19) | 11 (2–20) | 11 (0–22) | 11 (-2–23) |
|  | ASIR^*^ | 29.7 | 28.9 (22.2–35.6) | 27.6 (15.7–39.5) | 27.4 (9.8–45) | 27.3 (5–49.6) | 14.3 | 14.3 (9.9–18.8) | 14.4 (6.3–22.5) | 14.5 (2.9–26.2) | 14.5 (0–29) |
| Zona da Mata | Cases^*^ | 50 | 52 (34–70) | 50 (26–75) | 53 (22–83) | 54 (18–90) | 22 | 29 (15–44) | 29 (8–50) | 30 (3–57) | 31 (-2–64) |
|  | ASIR^*^ | 35.2 | 34.9 (27.2–42.6) | 34.9 (21.2–48.5) | 35.1 (17–53.1) | 35.1 (13.5–56.6) | 14.4 | 19 (12.5–25.5) | 19.1 (6.9–31.4) | 19.2 (3–35.4) | 19.2 (-0.1–38.5) |
| Non-metropolitan | Cases^*^ | 35 | 46 (30–61) | 44 (25–62) | 43 (20–66) | 44 (17–71) | 19 | 23 (11–35) | 23 (6–39) | 22 (2–43) | 22 (-1–46) |
|  | ASIR^*^ | 21.2 | 26 (21.4–30.6) | 25.2 (17.4–33.1) | 24.8 (13.9–35.7) | 24.7 (11.3–38.1) | 11 | 12.7 (8.5–16.9) | 12.7 (5.1–20.2) | 12.6 (2.6–22.7) | 12.6 (0.6–24.6) |
| Bahia | | | | | | | | | | | |
| Feira de Santana | Cases^*^ | 129 | 144 (95–193) | 163 (50–275) | 164 (7–321) | 164 (-30–358) | 83 | 60 (37–84) | 63 (21–104) | 64 (10–119) | 66 (-1–133) |
|  | ASIR^*^ | 30.5 | 32 (22.5–41.4) | 35.1 (11.5–58.6) | 35 (1.8–68.2) | 34.8 (-6.1–75.7) | 16.6 | 12.1 (8.5–15.8) | 12 (4.7–19.3) | 12 (2.2–21.7) | 11.9 (0.3–23.6) |
| Salvador | Cases^*^ | 1074 | 965 (808–1122) | 971 (619–1323) | 998 (498–1499) | 1029 (397–1660) | 601 | 513 (429–598) | 514 (345–682) | 533 (292–773) | 552 (248–855) |
|  | ASIR^*^ | 59.9 | 56.7 (48.2–65.2) | 57.3 (36.9–77.8) | 56.2 (28.2–84.1) | 55.8 (21.7–89.9) | 28.8 | 26.3 (22.6–30) | 26.2 (17.9–34.6) | 26.1 (14.5–37.6) | 26 (11.8–40.1) |
| Non-metropolitan | Cases^*^ | 1320 | 1452 (1234–1669) | 1576 (1039–2112) | 1636 (863–2409) | 1659 (694–2625) | 638 | 670 (525–815) | 718 (375–1061) | 753 (265–1242) | 770 (163–1377) |
|  | ASIR^*^ | 26.9 | 28.8 (24.8–32.9) | 31 (20.5–41.4) | 31.8 (16.9–46.8) | 31.8 (13.4–50.3) | 12.3 | 13.1 (10.4–15.7) | 13.8 (7.3–20.4) | 14.3 (5.1–23.5) | 14.3 (3.1–25.5) |
| Ceará | | | | | | | | | | | |
| Cariri | Cases^*^ | 80 | 116 (81–151) | 124 (57–190) | 130 (37–223) | 137 (19–254) | 49 | 40 (26–55) | 41 (21–62) | 44 (14–74) | 47 (7–86) |
|  | ASIR^*^ | 29.3 | 37.5 (28.4–46.6) | 38 (18.6–57.4) | 37.7 (11.5–63.9) | 37.6 (5.8–69.3) | 15.1 | 12 (9.7–14.4) | 11.7 (7–16.3) | 11.7 (4.6–18.9) | 11.7 (2.4–21) |
| Fortaleza | Cases^*^ | 1318 | 1464 (1261–1668) | 1596 (1080–2113) | 1663 (878–2448) | 1731 (686–2775) | 561 | 609 (494–723) | 636 (378–893) | 661 (294–1029) | 685 (220–1149) |
|  | ASIR^*^ | 68.1 | 75.7 (65.9–85.4) | 80.5 (54.7–106.3) | 79.9 (42.4–117.4) | 79.3 (31.6–127) | 25.9 | 28.6 (23.7–33.5) | 29.1 (17.5–40.7) | 29.2 (13.1–45.3) | 29.2 (9.5–48.8) |
| Sobral | Cases^*^ | 137 | 147 (110–184) | 152 (86–217) | 157 (69–245) | 162 (55–269) | 74 | 70 (47–93) | 71 (35–107) | 74 (27–121) | 77 (19–134) |
|  | ASIR^*^ | 59.2 | 60.1 (48.3–71.9) | 60.4 (36.1–84.6) | 60.6 (28.1–93.1) | 60.6 (21.5–99.6) | 30.2 | 27.7 (21.2–34.3) | 27.5 (15.1–40) | 27.5 (11.1–44) | 27.5 (7.8–47.2) |
| Non-metropolitan | Cases^*^ | 431 | 444 (375–513) | 481 (339–623) | 516 (303–729) | 534 (261–808) | 219 | 221 (166–277) | 224 (115–334) | 231 (81–381) | 238 (52–423) |
|  | ASIR^*^ | 23 | 23 (20.1–25.9) | 24.5 (17.6–31.5) | 25.4 (15.2–35.7) | 25.6 (12.7–38.5) | 11 | 11.3 (8.9–13.7) | 11.3 (6–16.6) | 11.3 (4.1–18.4) | 11.3 (2.6–19.9) |
| Maranhão | | | | | | | | | | | |
| Grande São Luís | Cases^*^ | 477 | 573 (368–778) | 628 (95–1161) | 625 (-101–1352) | 619 (-257–1495) | 216 | 254 (154–354) | 272 (31–514) | 278 (-54–609) | 281 (-123–685) |
|  | ASIR^*^ | 36 | 72.1 (47–97.3) | 76 (11.7–140.3) | 75.4 (-12–162.7) | 74.3 (-30.7–179.3) | 14.9 | 29 (18.2–39.9) | 29.5 (3.6–55.4) | 29.7 (-5.5–64.9) | 29.7 (-12.8–72.1) |
| Sudoeste Maranhense | Cases^*^ | 44 | 63 (36–89) | 65 (16–113) | 66 (-1–132) | 68 (-15–150) | 26 | 35 (16–54) | 36 (3–70) | 38 (-7–83) | 39 (-16–94) |
|  | ASIR^*^ | 25.4 | 33.7 (22.2–45.2) | 33.7 (9.6–57.8) | 33.3 (0.6–65.9) | 33.1 (-6.7–73) | 14.4 | 18 (10.3–25.7) | 18.2 (2.4–33.9) | 18.3 (-2.8–39.4) | 18.2 (-7–43.5) |
| Non-metropolitan | Cases^*^ | 617 | 775 (640–910) | 775 (486–1065) | 791 (392–1190) | 808 (315–1300) | 368 | 378 (294–462) | 381 (208–554) | 392 (154–631) | 402 (107–696) |
|  | ASIR^*^ | 28.2 | 31.5 (26.4–36.5) | 31.5 (20–43.1) | 31.5 (15.8–47.3) | 31.5 (12.4–50.7) | 16.2 | 15.4 (12.4–18.4) | 15.5 (8.6–22.3) | 15.5 (6.2–24.8) | 15.5 (4.2–26.7) |
| Rio Grande do Norte | | | | | | | | | | | |
| Natal | Cases^*^ | 346 | 575 (487–664) | 730 (480–980) | 793 (353–1233) | 828 (203–1453) | 153 | 164 (127–201) | 174 (108–240) | 185 (91–279) | 195 (74–316) |
|  | ASIR^*^ | 46.6 | 76.4 (66.4–86.4) | 93.9 (62.4–125.3) | 97 (43.6–150.5) | 96.6 (23.9–169.2) | 18.5 | 20 (16.7–23.4) | 20.6 (13.4–27.7) | 20.7 (10.6–30.8) | 20.7 (8.2–33.2) |
| Non-metropolitan | Cases^*^ | 220 | 247 (191–303) | 287 (158–417) | 309 (108–511) | 317 (54–581) | 76 | 91 (60–122) | 92 (37–147) | 95 (21–169) | 97 (7–187) |
|  | ASIR^*^ | 24.2 | 27.2 (22.1–32.4) | 31.5 (17.8–45.3) | 33 (11.8–54.2) | 33.1 (5.9–60.3) | 7.9 | 9.8 (7.2–12.5) | 9.8 (4.3–15.4) | 9.8 (2.4–17.3) | 9.8 (0.9–18.7) |
| Paraíba | | | | | | | | | | | |
| Araruna | Cases^*^ | 6 | 4 (0–8) | 4 (0–8) | 4 (0–8) | 4 (0–8) | 2 | 2 (-1–4) | 2 (-2–5) | 2 (-2–5) | 2 (-2–5) |
|  | ASIR^*^ | 18.6 | 13.2 (9.2–17.3) | 13.1 (8.3–18) | 13.1 (7.6–18.7) | 13.1 (6.9–19.3) | 6.3 | 4.9 (0.9–8.9) | 4.9 (-1.1–10.9) | 4.9 (-2.6–12.4) | 4.9 (-3.7–13.5) |
| Barra de Santa Rosa | Cases^*^ | 3 | 2 (-2–5) | 2 (-4–7) | 2 (-6–9) | 2 (-7–10) | 1 | 1 (-1–4) | 1 (-1–4) | 1 (-1–4) | 1 (-1–4) |
|  | ASIR^*^ | 7.5 | 4.4 (-2.5–11.3) | 4.4 (-9.1–17.9) | 4.4 (-13.4–22.2) | 4.3 (-16.6–25.2) | 2.5 | 3.5 (1.7–5.3) | 3.5 (1.4–5.6) | 3.5 (1.1–5.9) | 3.5 (0.9–6.2) |
| Cajazeiras | Cases^*^ | 21 | 21 (11–31) | 23 (9–37) | 24 (6–42) | 25 (3–46) | 8 | 10 (3–18) | 10 (1–20) | 11 (-1–22) | 11 (-3–24) |
|  | ASIR^*^ | 23.4 | 24.1 (18.2–29.9) | 26.1 (14.6–37.6) | 26.9 (10.1–43.6) | 26.9 (6–47.8) | 8.5 | 11.5 (6.7–16.3) | 11.5 (3.3–19.6) | 11.5 (0.9–22.1) | 11.5 (-1.1–24) |
| Campina Grande | Cases^*^ | 74 | 97 (65–130) | 102 (43–162) | 106 (25–187) | 110 (10–209) | 35 | 38 (19–57) | 40 (7–72) | 42 (-2–85) | 43 (-10–96) |
|  | ASIR^*^ | 23.7 | 30.4 (22.4–38.4) | 30.8 (13.9–47.7) | 30.9 (8.2–53.7) | 30.9 (3.5–58.2) | 10.1 | 11.1 (6.9–15.3) | 11.2 (2.7–19.6) | 11.2 (0–22.5) | 11.2 (-2.2–24.7) |
| Esperança | Cases^*^ | 6 | 14 (5–23) | 14 (3–26) | 15 (1–29) | 15 (-1–31) | 6 | 7 (1–12) | 7 (1–12) | 7 (1–12) | 7 (1–13) |
|  | ASIR^*^ | 8.8 | 20.5 (12.8–28.2) | 20.6 (7.6–33.6) | 20.6 (3.9–37.3) | 20.6 (0.9–40.3) | 8.5 | 9.1 (6.7–11.4) | 9.1 (6.1–12) | 9 (5.6–12.5) | 9 (5.2–12.9) |
| Guarabira | Cases^*^ | 46 | 44 (27–60) | 47 (24–71) | 49 (18–79) | 49 (13–86) | 13 | 15 (5–25) | 15 (1–29) | 15 (-2–32) | 15 (-5–35) |
|  | ASIR^*^ | 37.9 | 35.5 (27.5–43.5) | 38.3 (22.7–54) | 38.9 (17.1–60.7) | 38.9 (12.2–65.6) | 9.7 | 11.5 (6.6–16.5) | 11.5 (2.4–20.7) | 11.5 (-0.5–23.5) | 11.5 (-2.7–25.7) |
| Itabaiana | Cases^*^ | 14 | 20 (10–29) | 20 (10–30) | 20 (9–31) | 20 (9–32) | 5 | 8 (2–14) | 8 (1–16) | 9 (0–18) | 9 (-1–19) |
|  | ASIR^*^ | 21.4 | 29.3 (23.9–34.7) | 29.5 (22–37.1) | 29.6 (20.2–38.9) | 29.5 (18.7–40.4) | 7.3 | 11.2 (7.2–15.2) | 12.1 (5.6–18.5) | 12.5 (3.7–21.3) | 12.5 (1.8–23.2) |
| João Pessoa | Cases^*^ | 290 | 353 (281–426) | 408 (233–582) | 430 (165–695) | 450 (101–799) | 128 | 125 (89–162) | 132 (62–201) | 139 (43–235) | 147 (27–268) |
|  | ASIR^*^ | 47.2 | 56.2 (46.3–66.2) | 61.7 (35.9–87.4) | 62.1 (24.3–99.9) | 61.8 (14.2–109.5) | 18.5 | 18 (13.7–22.3) | 17.9 (9–26.9) | 17.9 (5.9–29.8) | 17.8 (3.5–32.2) |
| Patos | Cases^*^ | 30 | 34 (18–50) | 34 (11–57) | 35 (6–64) | 35 (1–69) | 6 | 10 (3–18) | 10 (2–19) | 11 (0–21) | 11 (-1–23) |
|  | ASIR^*^ | 27.1 | 30.6 (21–40.2) | 30.6 (12.9–48.4) | 30.6 (7.2–54) | 30.6 (2.6–58.5) | 4.6 | 8.8 (5.5–12.1) | 8.7 (3.5–13.9) | 8.7 (2.1–15.3) | 8.7 (0.9–16.5) |
| Sousa | Cases^*^ | 26 | 14 (4–25) | 15 (-2–31) | 15 (-6–36) | 15 (-10–41) | 6 | 5 (0–11) | 5 (-1–12) | 5 (-2–13) | 5 (-3–14) |
|  | ASIR^*^ | 44.5 | 25.2 (12–38.4) | 25.1 (-0.3–50.5) | 25 (-8.4–58.4) | 24.9 (-14.8–64.6) | 9.5 | 8.9 (4.2–13.7) | 8.9 (1.5–16.2) | 8.8 (-0.5–18.1) | 8.8 (-2.1–19.7) |
| Vale do Mamanguape | Cases^*^ | 12 | 16 (7–25) | 16 (6–26) | 17 (5–29) | 18 (4–31) | 4 | 6 (1–11) | 6 (0–12) | 6 (0–13) | 6 (-1–13) |
|  | ASIR^*^ | 22 | 25.9 (19.1–32.7) | 25.6 (15.3–35.9) | 25.6 (12.6–38.5) | 25.5 (10.4–40.7) | 6.7 | 9.5 (5.8–13.2) | 9.2 (4.1–14.3) | 9.1 (2.8–15.4) | 9.1 (1.8–16.3) |
| Vale do Piancó | Cases^*^ | 9 | 13 (5–21) | 13 (5–21) | 13 (4–22) | 13 (3–23) | 6 | 4 (0–8) | 3 (-1–8) | 3 (-2–9) | 3 (-3–10) |
|  | ASIR^*^ | 13.4 | 18.8 (14.2–23.3) | 18.8 (12.3–25.3) | 18.8 (10.6–27.1) | 18.8 (9.1–28.5) | 8.1 | 5.4 (2.8–8) | 5 (0.5–9.5) | 4.9 (-1.7–11.5) | 4.9 (-3.5–13.2) |
| Non-metropolitan | Cases^*^ | 48 | 55 (1–110) | 47 (-62–156) | 45 (-97–187) | 45 (-127–217) | 36 | 23 (8–39) | 24 (-3–51) | 24 (-12–59) | 24 (-18–67) |
|  | ASIR^*^ | 15.9 | 18.1 (0.9–35.2) | 15.3 (-19.9–50.5) | 14.4 (-31–59.7) | 14.2 (-40.2–68.5) | 11.5 | 7.4 (3.5–11.2) | 7.5 (-0.5–15.5) | 7.5 (-3.2–18.2) | 7.5 (-5.3–20.2) |
| Pernambuco | | | | | | | | | | | |
| Recife | Cases^*^ | 1907 | 1821 (1559–2083) | 1998 (1316–2679) | 2054 (1047–3061) | 2106 (803–3409) | 816 | 733 (618–848) | 756 (509–1004) | 786 (438–1134) | 810 (375–1245) |
|  | ASIR^*^ | 99.7 | 96.4 (83.3–109.6) | 104.6 (69.2–140) | 104.1 (53.3–155) | 103.5 (39.6–167.4) | 36.6 | 34.5 (29.6–39.3) | 35.1 (23.9–46.3) | 35.4 (19.9–50.8) | 35.4 (16.5–54.2) |
| Non-metropolitan | Cases^*^ | 901 | 1146 (1027–1264) | 1297 (999–1595) | 1342 (814–1871) | 1378 (617–2139) | 341 | 421 (330–512) | 442 (244–641) | 471 (188–754) | 493 (137–849) |
|  | ASIR^*^ | 36.1 | 43.3 (39.6–47) | 47.9 (37.2–58.6) | 47.8 (29.1–66.4) | 47.3 (21.3–73.3) | 12.5 | 15.2 (12.2–18.1) | 15.6 (8.7–22.4) | 15.8 (6.4–25.2) | 15.9 (4.5–27.3) |
| Piauí | | | | | | | | | | | |
| Non-metropolitan | Cases^*^ | 304 | 378 (162–593) | 407 (-151–964) | 433 (-374–1241) | 447 (-561–1456) | 172 | 181 (132–231) | 190 (89–291) | 199 (57–342) | 205 (27–384) |
|  | ASIR^*^ | 19.3 | 23.1 (10.1–36.1) | 24.5 (-9–58.1) | 25.5 (-22–73) | 25.7 (-32.2–83.6) | 10 | 10.7 (8.2–13.1) | 11 (5.4–16.6) | 11.1 (3.3–19) | 11.1 (1.6–20.7) |
| Sergipe | | | | | | | | | | | |
| Aracaju | Cases^*^ | 192 | 357 (281–434) | 490 (240–739) | 539 (98–981) | 561 (-58–1180) | 85 | 91 (67–114) | 103 (62–144) | 112 (48–177) | 119 (32–207) |
|  | ASIR^*^ | 44.3 | 80.3 (65.3–95.3) | 105.7 (52.6–158.7) | 109.7 (20.4–198.9) | 107.9 (-10.8–226.6) | 16.9 | 18.1 (15.2–21) | 19.6 (12.7–26.5) | 20 (9.1–30.9) | 19.9 (5.7–34.1) |
| Non-metropolitan | Cases^*^ | 178 | 215 (149–280) | 256 (87–425) | 278 (21–536) | 289 (-41–620) | 80 | 86 (53–118) | 90 (27–154) | 96 (7–185) | 101 (-10–212) |
|  | ASIR^*^ | 28.9 | 33.4 (24.2–42.6) | 39.3 (13.8–64.9) | 41.1 (3.4–78.8) | 41.1 (-5.6–87.8) | 12.1 | 12.9 (8.9–17) | 13.3 (4.4–22.3) | 13.5 (1.3–25.7) | 13.5 (-1.1–28.2) |
| **Southeast** | | | | | | | | | | | |
| Minas Gerais | | | | | | | | | | | |
| Belo Horizonte | Cases^*^ | 618 | 597 (453–741) | 633 (285–982) | 666 (158–1174) | 691 (42–1341) | 293 | 238 (171–304) | 241 (102–379) | 252 (59–446) | 264 (21–507) |
|  | ASIR^*^ | 21 | 21 (16.3–25.8) | 21.8 (9.9–33.7) | 22 (5.3–38.7) | 21.9 (1.4–42.4) | 9 | 7.7 (5.8–9.7) | 7.7 (3.4–11.9) | 7.6 (1.9–13.4) | 7.6 (0.7–14.6) |
| Vale do Aço | Cases^*^ | 56 | 66 (2–129) | 66 (-83–216) | 68 (-139–275) | 70 (-185–324) | 33 | 28 (0–56) | 28 (-34–90) | 29 (-57–114) | 29 (-76–134) |
|  | ASIR^*^ | 14.7 | 18.2 (1.1–35.2) | 18.2 (-22.6–59.1) | 18.3 (-37.1–73.6) | 18.2 (-48.1–84.5) | 8 | 7.3 (0.4–14.1) | 7.3 (-8.7–23.2) | 7.3 (-14.3–28.8) | 7.2 (-18.5–32.9) |
| Non-metropolitan | Cases^*^ | 1288 | 1425 (-695–3545) | 1800 (-4749–8349) | 2008 (-7889–11905) | 2078 (-10282–14438) | 513 | 510 (-223–1243) | 557 (-1395–2509) | 595 (-2225–3414) | 614 (-2897–4125) |
|  | ASIR^*^ | 17.2 | 20 (-9.7–49.7) | 24.8 (-65.5–115.1) | 26.9 (-105.5–159.3) | 27 (-133.5–187.4) | 6.6 | 7 (-3–17.1) | 7.5 (-18.8–33.8) | 7.8 (-29.1–44.6) | 7.8 (-36.7–52.2) |
| Rio de Janeiro | | | | | | | | | | | |
| Rio de Janeiro | Cases^*^ | 5519 | 6366 (5904–6828) | 6546 (5264–7829) | 6573 (4291–8855) | 6747 (3413–10082) | 2731 | 2678 (2454–2902) | 2750 (2240–3260) | 2881 (2112–3649) | 2987 (1986–3987) |
|  | ASIR^*^ | 84.5 | 103.3 (96.2–110.3) | 106.5 (85.8–127.2) | 103.4 (67.6–139.3) | 102.9 (52.1–153.6) | 38.2 | 39 (36.1–41.9) | 40.1 (32.8–47.3) | 40.4 (29.7–51.1) | 40.4 (26.9–53.9) |
| Non-metropolitan | Cases^*^ | 960 | 1076 (900–1252) | 1382 (837–1927) | 1579 (633–2526) | 1673 (366–2981) | 356 | 404 (328–480) | 433 (277–589) | 465 (242–688) | 493 (208–777) |
|  | ASIR^*^ | 46.8 | 53 (45–61.1) | 66 (40.2–91.7) | 71.5 (28.8–114.1) | 72.3 (15.9–128.6) | 16.3 | 18.7 (15.7–21.7) | 19.3 (12.6–26) | 19.5 (10.3–28.8) | 19.6 (8.4–30.7) |
| São Paulo | | | | | | | | | | | |
| Baixada Santista | Cases^*^ | 1034 | 1262 (777–1747) | 1421 (79–2763) | 1482 (-451–3415) | 1536 (-913–3985) | 421 | 478 (417–539) | 513 (400–626) | 542 (371–714) | 570 (343–797) |
|  | ASIR^*^ | 118.4 | 143.5 (88.9–198.1) | 158.6 (9.1–308.2) | 158.7 (-48.1–365.6) | 158 (-93.8–409.9) | 45.7 | 49.8 (45.3–54.3) | 52.1 (41.6–62.7) | 52.4 (36.4–68.4) | 52.3 (31.9–72.8) |
| Campinas | Cases^*^ | 527 | 498 (409–586) | 632 (376–888) | 724 (260–1188) | 766 (111–1422) | 166 | 187 (141–233) | 204 (111–298) | 218 (82–355) | 230 (53–407) |
|  | ASIR^*^ | 32.6 | 31.7 (26.7–36.6) | 38.9 (23.4–54.3) | 42.6 (15.5–69.7) | 43.2 (6.4–80.1) | 10.2 | 11.3 (9.1–13.6) | 11.9 (6.7–17.1) | 12.1 (4.7–19.5) | 12.1 (2.9–21.3) |
| Ribeirão Preto | Cases^*^ | 280 | 268 (174–363) | 340 (65–616) | 365 (-48–779) | 376 (-151–902) | 70 | 84 (61–107) | 95 (58–132) | 103 (50–155) | 107 (42–172) |
|  | ASIR^*^ | 33 | 32.7 (21.9–43.6) | 40 (7.9–72.2) | 41.6 (-5.3–88.6) | 41.5 (-16.5–99.5) | 8 | 9.8 (8.1–11.4) | 10.7 (7.1–14.3) | 11.1 (5.9–16.3) | 11.2 (4.7–17.6) |
| Sorocaba | Cases^*^ | 407 | 392 (294–489) | 476 (209–743) | 506 (104–908) | 522 (6–1038) | 98 | 117 (87–147) | 124 (74–174) | 131 (63–199) | 138 (54–223) |
|  | ASIR^*^ | 37.7 | 36.3 (28–44.6) | 42.1 (18.8–65.4) | 43.2 (9.1–77.3) | 43.1 (0.7–85.6) | 9 | 10.6 (8.6–12.6) | 10.6 (6.7–14.4) | 10.6 (5.4–15.7) | 10.5 (4.3–16.7) |
| São Paulo | Cases^*^ | 5508 | 5578 (3269–7886) | 6402 (-98–12902) | 6464 (-2472–15399) | 6477 (-4373–17327) | 2349 | 2493 (1611–3375) | 2756 (387–5126) | 2839 (-472–6150) | 2880 (-1176–6936) |
|  | ASIR^*^ | 52 | 54.3 (31.8–76.7) | 59.3 (-0.9–119.5) | 59.5 (-22.7–141.7) | 59.2 (-40–158.4) | 20.4 | 22.2 (14.4–30.1) | 23.6 (3.3–43.8) | 24 (-4–51.9) | 24 (-9.8–57.8) |
| Vale do Paraíba e Litoral Norte | Cases^*^ | 510 | 532 (434–631) | 565 (318–811) | 580 (185–974) | 601 (66–1136) | 172 | 171 (139–203) | 185 (132–238) | 199 (118–279) | 210 (103–317) |
|  | ASIR^*^ | 40.2 | 42.7 (35.7–49.8) | 44.2 (25.3–63.2) | 43.4 (14.1–72.7) | 43.1 (4.9–81.3) | 13 | 13.2 (11.6–14.7) | 13.8 (10.3–17.2) | 14 (8.7–19.3) | 14 (7.1–20.8) |
| Non-metropolitan | Cases^*^ | 2377 | 2386 (1931–2840) | 2877 (1461–4293) | 3160 (822–5498) | 3307 (142–6473) | 563 | 574 (500–648) | 598 (457–738) | 626 (418–833) | 652 (381–923) |
|  | ASIR^*^ | 37.1 | 38.5 (31.3–45.6) | 45.6 (23.2–67.9) | 48.2 (12.6–83.8) | 48.6 (2.1–95) | 8.7 | 9 (8.1–9.9) | 9.2 (7.1–11.2) | 9.2 (6.2–12.2) | 9.2 (5.4–12.9) |
| Espírito Santo | | | | | | | | | | | |
| Grande Vitória | Cases^*^ | 435 | 475 (359–591) | 501 (221–781) | 515 (110–920) | 533 (13–1053) | 218 | 180 (131–228) | 188 (91–284) | 198 (64–333) | 209 (40–378) |
|  | ASIR^*^ | 45.4 | 50 (38.6–61.4) | 50.6 (22.7–78.5) | 49.7 (10.9–88.6) | 49.3 (1.4–97.3) | 21.4 | 17.7 (13.7–21.7) | 17.7 (9–26.5) | 17.7 (6–29.5) | 17.7 (3.6–31.8) |
| Non-metropolitan | Cases^*^ | 254 | 244 (199–290) | 252 (173–330) | 263 (155–372) | 275 (139–411) | 109 | 104 (77–131) | 106 (65–147) | 112 (58–165) | 117 (51–183) |
|  | ASIR^*^ | 24.5 | 24.1 (20.8–27.5) | 24.2 (17.3–31.1) | 24.2 (14.7–33.7) | 24.2 (12.6–35.8) | 10.5 | 10.1 (8.4–11.9) | 10 (6.7–13.4) | 10 (5.6–14.5) | 10 (4.7–15.4) |
| **South** | | | | | | | | | | | |
| Santa Catarina | | | | | | | | | | | |
| Alto Vale do Itajaí | Cases^*^ | 19 | 14 (6–22) | 16 (5–27) | 17 (3–31) | 18 (1–34) | 8 | 7 (1–12) | 7 (2–13) | 8 (2–14) | 8 (2–15) |
|  | ASIR^*^ | 12.2 | 8.9 (6.4–11.4) | 9.7 (5–14.3) | 9.8 (3.2–16.4) | 9.8 (1.7–17.9) | 5.2 | 4.5 (3.4–5.6) | 4.5 (3.1–5.9) | 4.5 (2.8–6.2) | 4.5 (2.6–6.4) |
| Carbonífera | Cases^*^ | 95 | 101 (77–124) | 113 (76–151) | 122 (71–173) | 129 (67–191) | 43 | 42 (27–56) | 45 (28–63) | 49 (28–69) | 51 (27–75) |
|  | ASIR^*^ | 29.4 | 31.7 (27.4–35.9) | 33.5 (24.3–42.6) | 34.3 (21.4–47.1) | 34.3 (18.9–49.7) | 12.9 | 12.8 (10.8–14.8) | 13 (9.8–16.2) | 13.1 (8.9–17.3) | 13.1 (8.2–18.1) |
| Chapecó | Cases^*^ | 30 | 30 (18–41) | 32 (19–46) | 34 (19–49) | 36 (19–53) | 9 | 13 (5–20) | 14 (5–22) | 15 (5–24) | 16 (5–26) |
|  | ASIR^*^ | 11.5 | 11.2 (9.4–13) | 11.3 (8.7–13.9) | 11.3 (8.1–14.5) | 11.3 (7.5–15.1) | 3.4 | 4.8 (3.7–5.9) | 4.8 (3.3–6.3) | 4.8 (2.9–6.7) | 4.8 (2.6–7) |
| Contestado | Cases^*^ | 29 | 27 (15–39) | 27 (12–43) | 29 (10–47) | 30 (8–51) | 17 | 14 (5–23) | 14 (2–26) | 15 (0–29) | 15 (-2–33) |
|  | ASIR^*^ | 10.4 | 9.8 (7.4–12.2) | 9.7 (5.8–13.6) | 9.7 (4.6–14.7) | 9.7 (3.6–15.7) | 6.2 | 5 (3.1–7) | 5 (1.7–8.2) | 4.9 (0.7–9.2) | 4.9 (0–9.9) |
| Extremo Oeste | Cases^*^ | 12 | 13 (6–21) | 13 (5–22) | 14 (5–23) | 14 (5–24) | 4 | 6 (1–11) | 6 (1–12) | 6 (1–12) | 7 (1–12) |
|  | ASIR^*^ | 6.4 | 7.4 (5.9–8.9) | 7.4 (5.3–9.4) | 7.4 (4.9–9.9) | 7.4 (4.5–10.3) | 2.3 | 3.4 (2.5–4.4) | 3.4 (2.3–4.6) | 3.5 (2.1–4.9) | 3.5 (1.9–5) |
| Florianópolis | Cases^*^ | 265 | 242 (165–318) | 279 (90–468) | 294 (27–560) | 307 (-28–642) | 108 | 115 (90–140) | 128 (92–163) | 134 (89–179) | 141 (87–195) |
|  | ASIR^*^ | 42.1 | 37.3 (26.5–48.1) | 39.1 (13–65.1) | 39.4 (3.9–74.9) | 39.4 (-3.4–82.2) | 16.3 | 16.9 (14.8–19) | 17 (13.3–20.7) | 17 (12.1–21.9) | 17 (11.1–22.8) |
| Foz do Rio Itajaí | Cases^*^ | 169 | 180 (141–219) | 212 (132–292) | 238 (112–365) | 265 (89–440) | 82 | 104 (75–133) | 122 (69–176) | 138 (61–216) | 154 (52–256) |
|  | ASIR^*^ | 50.3 | 48.8 (41.1–56.6) | 49.4 (32.1–66.7) | 49.2 (23.8–74.6) | 49 (17.1–81) | 23.4 | 27.2 (21.6–32.7) | 27.5 (16.5–38.5) | 27.6 (12.8–42.4) | 27.6 (9.8–45.3) |
| Lages | Cases^*^ | 23 | 28 (16–39) | 28 (15–42) | 29 (13–45) | 30 (12–48) | 12 | 11 (2–20) | 11 (-2–25) | 12 (-5–28) | 12 (-8–32) |
|  | ASIR^*^ | 12.3 | 15.1 (12.2–18) | 15.2 (10.6–19.8) | 15.3 (9.1–21.4) | 15.3 (7.8–22.7) | 6 | 6 (2.7–9.3) | 6 (-0.2–12.2) | 6 (-2.1–14.1) | 6 (-3.7–15.6) |
| Norte/Nordeste Catarinense | Cases^*^ | 209 | 204 (167–241) | 227 (165–290) | 245 (152–338) | 260 (137–384) | 86 | 86 (59–113) | 93 (46–141) | 101 (34–167) | 107 (24–191) |
|  | ASIR^*^ | 28.9 | 27.8 (24.5–31) | 28.8 (21.9–35.8) | 29.2 (18.8–39.6) | 29.2 (15.8–42.5) | 11.7 | 11.6 (8.9–14.3) | 11.7 (6.2–17.2) | 11.7 (4.4–19.1) | 11.7 (2.9–20.6) |
| Tubarão | Cases^*^ | 52 | 51 (35–66) | 53 (35–72) | 56 (35–78) | 59 (34–83) | 23 | 22 (12–32) | 23 (11–34) | 24 (11–37) | 25 (11–40) |
|  | ASIR^*^ | 25.3 | 25.2 (21.6–28.8) | 25.3 (19.7–30.8) | 25.3 (18.2–32.3) | 25.3 (17–33.6) | 10.9 | 10.5 (8.5–12.5) | 10.5 (7.6–13.5) | 10.5 (6.8–14.2) | 10.5 (6.2–14.9) |
| Vale do Itajaí | Cases^*^ | 142 | 114 (79–149) | 138 (62–214) | 149 (34–264) | 157 (7–308) | 49 | 48 (33–63) | 52 (34–70) | 56 (34–78) | 59 (33–85) |
|  | ASIR^*^ | 35.2 | 27.6 (20.9–34.3) | 31 (14.7–47.3) | 31.5 (7.8–55.2) | 31.3 (1.7–60.8) | 11.5 | 11.4 (9.7–13.1) | 11.4 (8.8–14) | 11.4 (8–14.8) | 11.4 (7.4–15.4) |
| Paraná | | | | | | | | | | | |
| Apucarana | Cases^*^ | 29 | 31 (18–44) | 31 (13–50) | 31 (6–56) | 31 (1–62) | 7 | 9 (2–17) | 9 (-1–20) | 10 (-3–22) | 10 (-5–25) |
|  | ASIR^*^ | 18.8 | 20.7 (15.6–25.8) | 21 (10.9–31.1) | 20.7 (5.8–35.6) | 20.6 (1.7–39.5) | 4.8 | 6 (2.9–9.1) | 6 (0.6–11.5) | 6 (-1.1–13.1) | 6 (-2.4–14.4) |
| Campo Mourão | Cases^*^ | 39 | 43 (27–59) | 46 (22–70) | 47 (14–79) | 47 (7–86) | 16 | 15 (6–23) | 15 (5–25) | 15 (3–27) | 15 (2–29) |
|  | ASIR^*^ | 22.2 | 25.2 (19.6–30.8) | 27.1 (15.5–38.8) | 27.2 (10.1–44.2) | 27 (5.6–48.5) | 8 | 8.3 (5.8–10.8) | 8.4 (4.4–12.4) | 8.4 (3.2–13.6) | 8.4 (2.3–14.5) |
| Cascavel | Cases^*^ | 57 | 56 (39–73) | 59 (38–81) | 62 (36–89) | 65 (33–96) | 15 | 23 (12–34) | 26 (10–41) | 28 (7–49) | 29 (4–55) |
|  | ASIR^*^ | 19.6 | 20.4 (17.4–23.5) | 20.8 (15.5–26) | 20.9 (13.7–28.1) | 20.9 (12.1–29.7) | 5.3 | 8 (6–10.1) | 8.6 (4.5–12.6) | 8.8 (3.1–14.5) | 8.9 (1.8–15.9) |
| Curitiba | Cases^*^ | 391 | 332 (269–395) | 339 (219–459) | 356 (189–524) | 374 (163–586) | 170 | 166 (121–212) | 172 (82–262) | 181 (55–308) | 191 (32–350) |
|  | ASIR^*^ | 22.1 | 18.9 (16–21.8) | 18.7 (12.4–25.1) | 18.7 (10.1–27.3) | 18.7 (8.3–29.1) | 8.9 | 8.9 (6.9–11) | 8.9 (4.5–13.4) | 8.9 (2.9–15) | 8.9 (1.6–16.2) |
| Londrina | Cases^*^ | 129 | 196 (151–240) | 266 (145–387) | 306 (98–514) | 323 (40–606) | 46 | 54 (34–73) | 59 (28–91) | 64 (21–107) | 67 (14–121) |
|  | ASIR^*^ | 23.7 | 36.7 (30.1–43.2) | 48.6 (27.2–69.9) | 53.2 (17.5–89) | 53.6 (7–100.3) | 8.1 | 9.4 (7.2–11.7) | 10.1 (5.5–14.7) | 10.4 (3.9–16.8) | 10.4 (2.5–18.3) |
| Maringá | Cases^*^ | 57 | 82 (59–104) | 94 (57–130) | 101 (48–153) | 106 (39–173) | 29 | 25 (12–38) | 26 (8–44) | 27 (5–50) | 29 (2–56) |
|  | ASIR^*^ | 13.7 | 19.9 (16.5–23.3) | 21.5 (14.3–28.8) | 22 (11.4–32.6) | 22 (8.7–35.3) | 6.5 | 5.7 (3.9–7.6) | 5.6 (2.5–8.8) | 5.6 (1.5–9.7) | 5.6 (0.7–10.4) |
| Toledo | Cases^*^ | 32 | 43 (28–59) | 48 (23–72) | 51 (13–88) | 53 (3–103) | 19 | 14 (6–22) | 14 (5–24) | 15 (5–26) | 16 (4–28) |
|  | ASIR^*^ | 15.8 | 21.1 (16.8–25.3) | 22.1 (12.5–31.7) | 22.3 (7–37.7) | 22.3 (2.3–42.3) | 9 | 6.5 (4.9–8.2) | 6.5 (4.1–8.9) | 6.5 (3.4–9.5) | 6.5 (2.9–10) |
| Umuarama | Cases^*^ | 32 | 34 (7–61) | 35 (-23–92) | 35 (-42–112) | 35 (-58–129) | 13 | 22 (9–35) | 23 (2–43) | 23 (-3–49) | 24 (-7–55) |
|  | ASIR^*^ | 18.9 | 21 (5.9–36.1) | 20.9 (-13.1–55) | 20.7 (-24.7–66.2) | 20.6 (-33.7–74.9) | 7.1 | 13.4 (7.9–18.9) | 13.2 (2.7–23.6) | 13.1 (-0.6–26.8) | 13 (-3.2–29.3) |
| Non-metropolitan | Cases^*^ | 518 | 539 (465–614) | 602 (433–772) | 629 (350–909) | 646 (263–1029) | 205 | 215 (162–269) | 224 (118–329) | 234 (88–380) | 243 (61–425) |
|  | ASIR^*^ | 25.7 | 26.9 (23.9–29.9) | 29.4 (21.5–37.4) | 29.7 (16.7–42.7) | 29.5 (12.2–46.8) | 10 | 10.6 (8.4–12.8) | 10.7 (5.9–15.5) | 10.7 (4.2–17.2) | 10.7 (2.8–18.6) |
| Rio Grande do Sul | | | | | | | | | | | |
| Porto Alegre | Cases^*^ | 1624 | 1721 (1530–1912) | 1935 (1434–2437) | 2025 (1232–2817) | 2056 (1010–3103) | 795 | 726 (624–829) | 731 (526–936) | 747 (466–1029) | 761 (415–1106) |
|  | ASIR^*^ | 75.8 | 85.6 (77–94.2) | 96.7 (72–121.4) | 99.2 (60.6–137.8) | 98.8 (48.7–148.9) | 34.5 | 33.2 (29.2–37.2) | 33.5 (24.4–42.6) | 33.7 (21.3–46.2) | 33.8 (18.6–48.9) |
| Serra Gaúcha | Cases^*^ | 115 | 162 (109–216) | 218 (60–377) | 243 (-16–502) | 248 (-95–592) | 46 | 53 (35–71) | 59 (32–86) | 63 (27–100) | 66 (20–112) |
|  | ASIR^*^ | 27.2 | 40.8 (28.9–52.7) | 52.9 (15.1–90.6) | 56.6 (-3.3–116.5) | 56 (-21–132.9) | 10.5 | 12.7 (10.1–15.4) | 13.7 (8.5–18.8) | 14 (6.6–21.3) | 14 (4.8–23.1) |
| Non-metropolitan | Cases^*^ | 961 | 1246 (1085–1407) | 1649 (1145–2154) | 1809 (944–2673) | 1843 (662–3023) | 443 | 443 (373–514) | 496 (341–650) | 524 (292–756) | 535 (237–833) |
|  | ASIR^*^ | 29.8 | 41 (36.2–45.7) | 53.9 (37.6–70.2) | 58.6 (30.7–86.5) | 58.9 (21.3–96.5) | 13.3 | 14 (12.2–15.8) | 15.5 (10.9–20.2) | 16.1 (9.1–23.1) | 16.1 (7.3–25) |
| **Central-West** | | | | | | | | | | | |
| Distrito Federal | | | | | | | | | | | |
| Non-metropolitan | Cases^*^ | 179 | 198 (149–248) | 207 (108–306) | 215 (75–356) | 224 (45–403) | 83 | 79 (54–104) | 82 (41–122) | 85 (30–140) | 88 (19–157) |
|  | ASIR^*^ | 13.4 | 14.2 (11.2–17.1) | 14.3 (7.7–20.8) | 14 (5.1–23) | 13.9 (2.9–24.8) | 5.6 | 5.2 (4–6.3) | 5.2 (2.9–7.5) | 5.1 (2–8.3) | 5.1 (1.2–8.9) |
| Goiás | | | | | | | | | | | |
| Goiânia | Cases^*^ | 342 | 373 (304–443) | 460 (282–638) | 500 (210–790) | 522 (128–916) | 111 | 103 (76–129) | 108 (68–147) | 115 (62–167) | 122 (57–187) |
|  | ASIR^*^ | 27.5 | 29.5 (24.9–34.1) | 34.4 (21.4–47.3) | 35.4 (15.1–55.7) | 35.2 (8.8–61.5) | 8.2 | 7.7 (6.4–8.9) | 7.6 (5.2–9.9) | 7.5 (4.4–10.7) | 7.5 (3.7–11.3) |
| Non-metropolitan | Cases^*^ | 291 | 307 (261–353) | 343 (251–435) | 372 (208–535) | 392 (157–627) | 127 | 112 (82–142) | 120 (67–172) | 127 (50–204) | 134 (33–235) |
|  | ASIR^*^ | 13.5 | 13.8 (12.4–15.2) | 14.7 (11–18.3) | 15 (8.6–21.4) | 15 (6.1–23.8) | 5.8 | 5 (4.1–6) | 5.1 (3–7.1) | 5.1 (2.1–8) | 5 (1.4–8.7) |
| Mato Grosso | | | | | | | | | | | |
| Vale do Rio Cuiabá | Cases^*^ | 308 | 406 (157–654) | 432 (-206–1070) | 428 (-431–1286) | 429 (-613–1472) | 126 | 192 (66–318) | 202 (-111–516) | 209 (-229–647) | 216 (-329–762) |
|  | ASIR^*^ | 59.9 | 75.4 (29.7–121.1) | 76.6 (-36.4–189.7) | 74.6 (-75–224.2) | 73.6 (-105–252.1) | 23.3 | 34.7 (12.4–57.1) | 34.4 (-18.7–87.4) | 34.3 (-37.3–105.8) | 34.2 (-51.9–120.3) |
| Non-metropolitan | Cases^*^ | 419 | 385 (309–462) | 450 (266–633) | 473 (194–751) | 495 (117–873) | 227 | 192 (140–244) | 225 (107–342) | 251 (74–427) | 269 (37–501) |
|  | ASIR^*^ | 36.6 | 29.9 (24.8–35) | 32.4 (19.5–45.3) | 32.1 (13.4–50.8) | 31.7 (7.6–55.8) | 20.8 | 15.6 (12–19.2) | 16.7 (8.3–25.2) | 17.4 (5.3–29.5) | 17.4 (2.5–32.3) |
| Mato Grosso do Sul | | | | | | | | | | | |
| Non-metropolitan | Cases^*^ | 560 | 775 (616–933) | 799 (415–1182) | 778 (233–1323) | 795 (82–1508) | 178 | 220 (171–269) | 234 (140–329) | 247 (113–381) | 260 (88–431) |
|  | ASIR^*^ | 41.8 | 55.6 (45–66.3) | 54.9 (28.8–81.1) | 50.9 (15.4–86.3) | 49.6 (5.3–93.9) | 12.9 | 15.6 (12.8–18.4) | 15.8 (9.8–21.9) | 15.8 (7.5–24.2) | 15.8 (5.5–26) |

*Abbreviations:* ASIR, age-standardized incidence rate; CrI, credible interval.

^*^ The point estimates (95% CrIs) were derived from the median values (2.5th and 97.5th percentiles).
